# Photoredox-catalyzed multicomponent Petasis reaction in batch and continuous flow with alkyl boronic acids

**DOI:** 10.1016/j.isci.2021.103134

**Published:** 2021-09-15

**Authors:** Monica Oliva, Prabhat Ranjan, Serena Pillitteri, Guglielmo Attilio Coppola, Monica Messina, Erik V. Van der Eycken, Upendra Kumar Sharma

**Affiliations:** 1 Laboratory for Organic and Microwave-Assisted Chemistry (LOMAC), Department of Chemistry, KU Leuven, Celestijnenlaan 200F, Leuven 3001, Belgium; 2 Peoples' Friendship University of Russia (RUDN University), Miklukho-Maklaya Street 6, Moscow 117198, Russia

**Keywords:** Catalysis, Chemistry, Engineering, Green chemistry

## Abstract

Multicomponent reactions (MCRs) are ideal platforms for the generation of a wide variety of organic scaffolds in a convergent and atom-economical manner. Many strategies for the generation of highly substituted and diverse structures have been developed and among these, the Petasis reaction represents a viable reaction manifold for the synthesis of substituted amines *via* coupling of an amine, an aldehyde and a boronic acid (BA). Despite its synthetic utility, the inherent drawbacks associated with the traditional two-electron Petasis reaction have stimulated continuous research towards more facile and tolerant methodologies. In this regard, we present the use of free alkyl BAs as effective radical precursors in this MCR through a single-electron transfer mechanism under mild reaction conditions. We have further demonstrated its applicability to photo-flow reactors, facilitating the reaction scale-up for the rapid assembly of complex molecular structures.

## Highlights

• Multicomponent photocatalyzed Petasis reaction
• Boronic acids as alkyl radical precursors under mild reaction conditions
• Photo-flow chemistry application for Petasis reaction

## Graphical abstract

**Figure:**
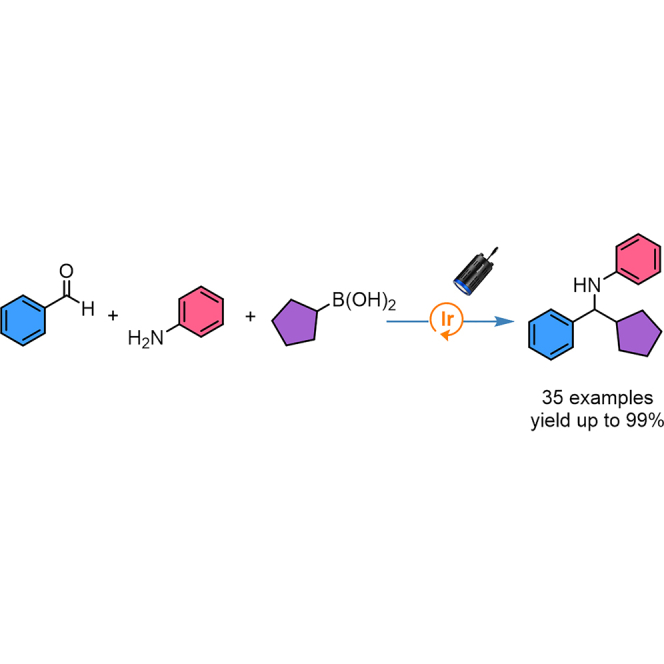

## Introduction

The discovery of multicomponent reactions (De Graaff et al., 2012; Cioc et al., 2014; Wu and Nielsen, 2018; Tan and Yudin, 2018; Abdelraheem et al., 2018) has had a tremendous impact on syntheses design. Starting from a handful of famous name-reactions (*viz*. Biginelli, Hantzsch, Mannich, Passerini, Strecker, and Ugi), a myriad of variants and niche applications branched out covering alternative coupling partners, reactivity pathways, and design strategies (Dömling et al., 2012; Gu, 2012; Touré and Hall, 2009). Atom economic and step-efficient processes coupled with selective sequential reactions have soon delineated MCRs as elegant routes toward complex structures in a diversity-oriented manner. Hence novel enabling techniques in organic synthesis have been promptly applied to MCRs. This has already been the case for chiral auxiliaries (Nunes et al., 2020; Ramón and Yus, 2005), microwave irradiation (Fairoosa et al., 2020), flow chemistry (Sharma and Van der Eycken, 2018), electrochemistry (Jiang et al., 2018), and photoredox catalysis (Qiu et al., 2018; Lan et al., 2017; Garbarino et al., 2016). Photoredox catalysis has recently gained new popularity as it allows us to explore novel radical processes in a mild and selective fashion. Low temperatures and irradiation energies minimize the occurrence of side reactions while expanding the scope of tolerated functional groups. Several reports demonstrated the potential of merging photocatalysis and MCRs opening the way to new cascade radical processes or shining new light on conventional coupling partners.

**Figure 1:**
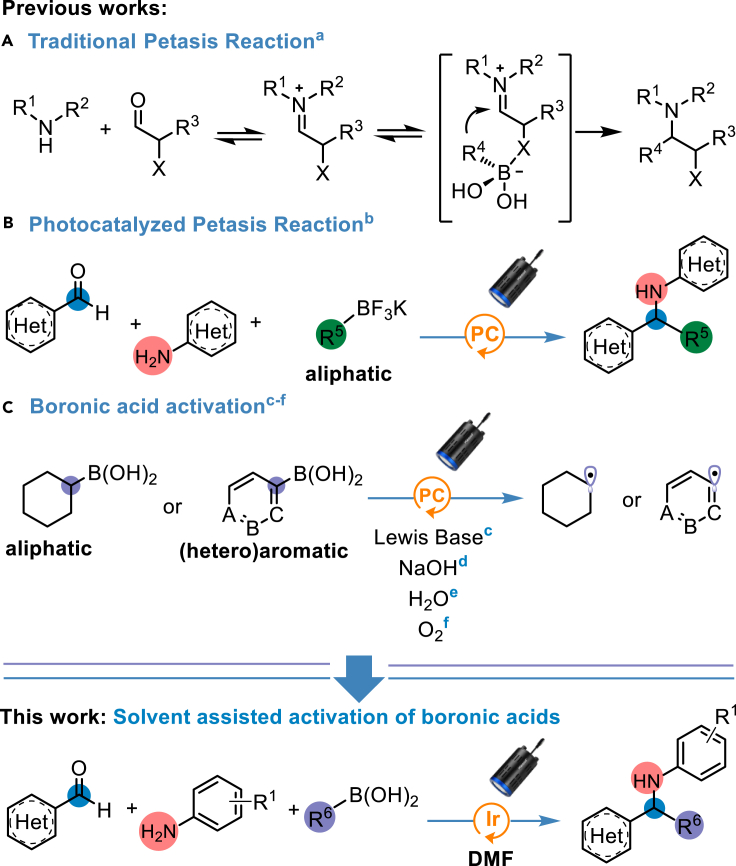
Background to Petasis Reaction and activation of boronic acids as radical precursors a. Petasis and Akritopoulou (1993), b. Yi et al. (2019), c. Lima et al. (2017), d. Iwata et al. (2018), e. Chilamari et al. (2020), f. Dong et al. (2020).

In this regard, traditional three-component Petasis reaction has been widely exploited in the generation of complex amines, important scaffolds in drug discovery and agrochemical industry (Wu et al., 2019). The key step involved in the traditional Petasis reaction (Petasis and Akritopoulou, 1993) is the generation of an active boron “ate” intermediate followed by nucleophilic addition to an imine or iminium ion, derived from a condensation reaction of an amine and an aldehyde (Figure 1A). However, the inherent requirement of a directing group to form the borate intermediate and the necessity to stabilize the negative charge on the migrating group severely restrict the broad utilization of this reaction. Moreover, being water the byproduct of imine formation, activation strategies which require dry conditions, as in the case of several photocatalyzed processes, are usually limited to *ex situ* prepared imines (Garrido-Castro et al., 2020).

In this context, Molander and co-workers reported an efficient single-electron transfer mechanism to perform traditional Petasis reaction utilizing trifluroborate salts to generate a wide range of secondary amines (Figure 1B) (Yi et al., 2019). The generation of alkyl radicals from trifluoroborate salts and its application in the functionalization of C(*sp)*^*2*^ atoms has been widely explored under both photoredox and electro-catalysis by several groups (Tellis et al., 2014; Koike and Akita, 2016; Stache et al., 2017; Primer and Molander, 2017; Niu et al., 2020). Nonetheless, the use of free boronic acids (BAs) as an alkyl radical source has been overlooked due to their high oxidation potential (Figure 1C) (Li et al., 2016). Recently, our group has reported the generation of alkyl radicals from BAs by modulating their oxidation potential through hydrogen bonding and facile generation of electron rich borate species with an amide-based solvent (Ranjan et al., 2021). Interestingly, the reaction efficiency was not majorly affected by the presence of water, an added value that assures the feasibility of the multicomponent Petasis reaction in our presented method.

Due to the long-standing interest of our group in MCRs and their application to generate complex scaffolds, we herein report the successful photoredox-catalyzed multicomponent Petasis reaction using BAs as an alkyl radical source. We also present for the first time the successful implementation of our method under continuous photo-flow conditions for the rapid generation of complex secondary amines.

## Results and discussion

### Reaction optimization

Based on our previous report (Ranjan et al., 2021), we commenced our investigation using *p*-anisaldehyde **1a**, aniline **2a,** and cyclopentyl BA **3a** as coupling partners in the presence of sodium bisulfate and 4-CzIPN as a photocatalyst in DMA. After irradiating the mixture with blue light for 24 h, the desired product **6a** was isolated in 45% yield (entry 1, Table 1). Encouraged by this result, we screened different amide-based solvents and photocatalysts under our reaction conditions. To our delight, [Ir{dF(CF_3_)ppy}_2_(dtbbpy)]PF_6_ as photocatalyst and DMF as a solvent afforded the desired product in 76% isolated yield (entry 4, Table 1). Interestingly, the reaction was also amenable, although in lower yield, in 1,4-dioxane, as a result of the possible activation of the BA by means of Lewis acid-base interactions with the *in situ* formed imine. Finally, control experiments established the necessity of photocatalyst, visible light, and the inert environment for the successful generation of the desired product (entries 16-18).

**Table 1:** Optimization table


Entry	Photocatalyst	PC amount	Solvent	Yield%^a^
1	4-CzIPN	5 mol%	DMA	45
2	4-CzIPN	5 mol%	DMF	59
3	4-CzIPN	5 mol%	1,4-dioxane	25
4	[Ir{dF(CF_3_)ppy}_2_(dtbbpy)]PF_6_	3.5 mol%	DMF	76
5	[Ir{dF(CF_3_)ppy}_2_(dtbbpy)]PF_6_	3.5 mol%	DMSO	37
6	[Ir{dF(CF_3_)ppy}_2_(dtbbpy)]PF_6_	3.5 mol%	ACN	51
7	[Ir{dF(CF_3_)ppy}_2_(dtbbpy)]PF_6_	3.5 mol%	DMF/ACN 3:1	53
8	4CzIPN	3.5 mol%	DMF	47
9	3-ClCzIPN	3.5 mol%	DMF	38
10	Mes-Acr^+^ClO_4_^-^	3.5 mol%	DMF	ND
11	Eosin-Y	3.5 mol%	DMF	ND
12	[Ru(bpy)_3_]Cl_2_^**.**^ 6H_2_O	3.5 mol%	DMF	20
13	[Ir(dtbbpy) (ppy)_2_]PF_6_	3.5 mol%	DMF	58
14	Ir(ppy)_3_	3.5 mol%	DMF	ND
15^b^	[Ir{dF(CF_3_)ppy}_2_(dtbbpy)]PF_6_	3.5 mol%	DMF	55
16	–	–	DMF	ND
17^c^	[Ir{dF(CF_3_)ppy}_2_(dtbbpy)]PF_6_	3.5 mol%	DMF	ND
18^d^	[Ir{dF(CF_3_)ppy}_2_(dtbbpy)]PF_6_	3.5 mol%	DMF	ND
19	[Ir{dF(CF_3_)ppy}_2_(dtbbpy)]PF_6_	3.5 mol%	Acetone/Methanol	ND

a Reaction conditions: a solution of **1a** (0.2 mmol), **2a** (0.3 mmol), **3a** (0.4 mmol) in DMF (2 mL) in the presence of the appropriate photocatalyst and NaHSO_4_ (0.2 mmol) was irradiated with blue light for 24h under Ar atmosphere. The yields were based on isolated products.

b No NaHSO_4_.

c No light.

d No inert atmosphere.

### Substrate scope

**Figure 2:**
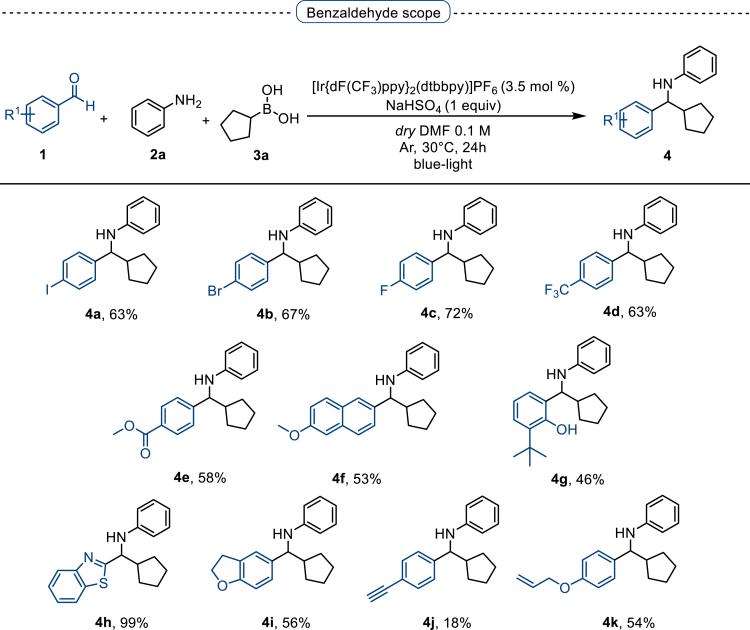
Benzaldehyde scope Reaction conditions: a solution of **1** (0.4 mmol), **2a** (0.6 mmol), **3a** (0.8 mmol) in DMF (4 mL) in the presence of [Ir{dF(CF_3_)ppy}_2_(dtbbpy)]PF_6_ (3.5 mol %) and NaHSO_4_ (0.4 mmol) was irradiated with blue light for 24h under Ar atmosphere. The yields were based on isolated products.

With the optimized conditions in hands, we first started to access the scope of aldehyde. Considering the possibility of late-stage functionalization of halo-substituted aldehyde, different halo-substituted benzaldehyde derivatives afforded good yields (Figure 2, **4a**-**4c**). Electron-withdrawing and electron donating substituents on the aromatic ring were equally well tolerated, delivering the desired products in good to moderate yields (Figure 2, **4d**-**4g)**. The presence of a free hydroxyl group at the *ortho* position of benzaldehyde, as in the case of **4g**, also resulted in product formation albeit in moderate yield. Interestingly, benzo[d]thiazole-2-carbaldehyde (**4h**) afforded quantitative yield and 2,3-dihydrobenzofuran (**4i**) showed moderate yield under our optimized reaction conditions. Considering the omnipresent nature of heterocyclic motifs in pharmaceutically relevant molecules, these results show the possibility of promising applications in medicinal chemistry.

**Figure 3:**
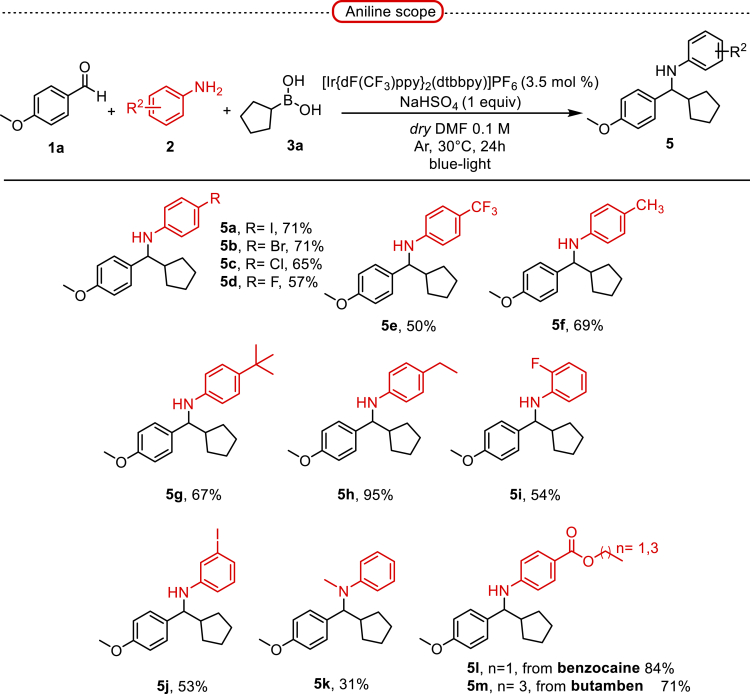
Aniline scope Reaction conditions: a solution of **1a** (0.4 mmol), **2** (0.6 mmol), **3a** (0.8 mmol) in DMF (4 mL) in the presence of [Ir{dF(CF_3_)ppy}_2_(dtbbpy)]PF_6_ (3.5 mol %) and NaHSO_4_ (0.4 mmol) was irradiated with blue light for 24h under Ar atmosphere. The yields were based on isolated products.

Next, we screened various aniline derivatives containing a diverse set of functional groups (Figure 3). Interestingly, the electronic effect of halo-groups on the aniline moiety had a minor influence on the outcome of the reaction in comparison with the halo-substituted benzaldehyde derivatives (**4c** *vs* **5d**). Moreover, there was no significant impact of steric hindrance on the reaction yield (**5i**). Aniline containing electron-donating groups also delivered the desired products in good yield (**5g**, **5h**). In addition, the reaction conditions were amenable to deliver derivatives of two local anesthetics (benzocaine **5l** and butamben **5m)** in 84% and 71% yield, respectively.

**Figure 4:**
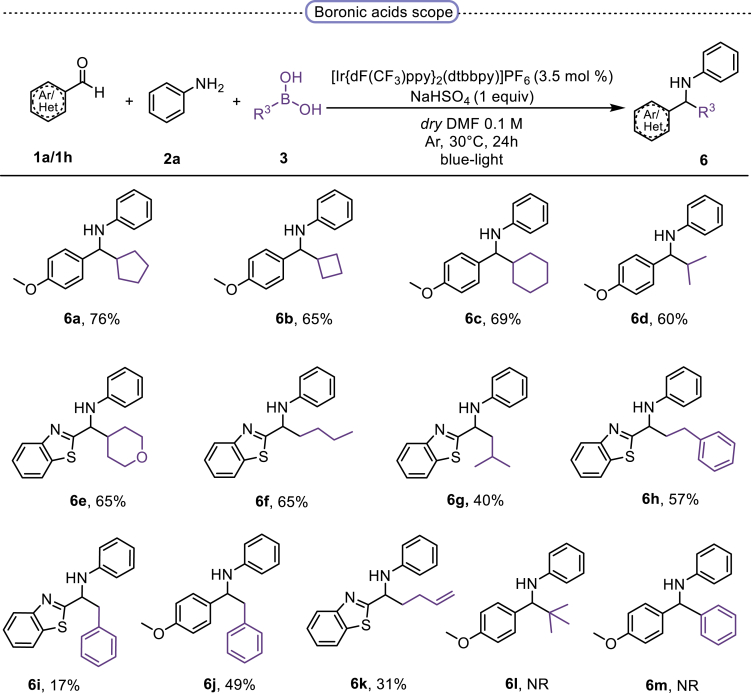
Boronic acids scope Reaction conditions: a solution of **1a** or **1h** (0.4 mmol), **2a** (0.6 mmol), **3** (0.8 mmol) in DMF (4 mL) in the presence of [Ir{dF(CF_3_)ppy}_2_(dtbbpy)]PF_6_ (3.5 mol %) and NaHSO_4_ (0.4 mmol) was irradiated with blue light for 24h under Ar atmosphere. The yields were based on isolated products.

Finally, we evaluated the scope of BAs under our optimized reaction conditions (Figure 4). Non-activated secondary BAs, including a heterocyclic unit, successfully afforded the desired products in good yield (**6a**-**6e**). Pleasingly, non-stabilized primary BAs were also successfully coupled to deliver the desired products in moderate to good yield (**6f**-**6j**). In the case of benzyl BA, the use of the electron-poor benzo[d]thiazole-2-carbaldehyde resulted in a low yield but given the less nucleophilic nature of the benzyl radical, the use the electron-rich *p*-anisaldehyde was beneficial and afforded the desired product in 49% yield. Notably, but-3-en-1-yl BA underwent the reaction as well to afford the desired product **6k** containing an alkene moiety. Phenyl BA and tertiary alkyl BA failed in delivering the desired products.

### Flow application

Encouraged by the results obtained in batch, we evaluated the feasibility of the Petasis reaction under continuous flow conditions. In recent years, photo-flow chemistry has gained ample attention to scale-up the chemical process (Noël, 2016), thanks to good mass transfer and better light-penetration effect. We started the optimization of our reaction using a Vapourtec E-150 module equipped with standard blue LEDs. Unfortunately, we observed very low conversion, which could be due to poor solubility of NaHSO_4_ and therefore less iminium ion formation (Table 2, entry 3). To overcome this drawback, we employed an excess of BA to facilitate the formation of iminium ion through the interaction with BA itself and favor the formation of the desired product. To further improve the yield, we screened the combination of two solvents, which was beneficial in our previous report (Ranjan et al., 2021). To our delight, we obtained the desired product in 78% yield using a mixture of DMF and ACN (3:1) (Table 2, entry 2). Finally, by increasing the power of the blue light from 60 W to 150 W, we could achieve 85% yield within 50 min, as a result of the increased photon-flux on the reaction mixture (Table 2, entry 1). In order to make a comparison between batch and flow reactor, a batch reaction without stirring and a stop-flow experiment were performed (entries 6 and 7, Table 2). We observed trace amount of desired product in the case of batch reactor and moderate yield under stop-flow conditions. The higher yield observed in the flow reactor under similar reaction conditions clearly shows the improved light-penetration effect and better mixing due to the smaller reactor diameter. From the scope of this continuous-flow protocol, it became clear that a continuous-flow approach was particularly beneficial in case of **6a**, **6j,** and **6k**, as a result of the shorter reaction time and hence improved efficiency (Table 2).

**Table 2:** Optimization and scope for flow application


Entry	Deviations from standard conditions^a^	Yield^b^
1	None	85%
2	DMF/ACN 3:1, 60W	78%
3	DMF, NaHSO_4_ (1 equiv)	traces
4	45°C	49%
5	Catalyst loading = 2.5 mol%	71%
6^c^	In batch under blue-lights (2 × 40W)	traces
7	Stop-flow	37%
Scope:		

a Reaction conditions: solution A: **1a** (0.2 mmol), **3a** (0.6 mmol) and iridium photocatalyst (3.5 mol%) in DMF/ACN (2 mL). Solution B: **2a** (0.3 mmol) in DMF/ACN (2 mL). Solution A and B were pumped at 0.1 mL/min.

b The yields were based on isolated products.

c No stirring.

### Mechanistic investigations

In order to shed some light on the reaction mechanism, cyclic voltammetry experiments were performed. When DMF was added to a solution of phenyl ethyl BA, a new oxidation peak appeared at 0.62 V *vs.* SCE (Figure S2). This result supported our assumption regarding the formation of activated species through the interaction between substrate and solvent, with concomitant decrease in the oxidation potential. Nonetheless, a concomitant activation through the interaction between BA and imine cannot be excluded as well (Lima et al., 2017). Additionally, fluorescence quenching experiments revealed that the exited state of the photocatalyst could undergo SET with the transient species formed by the mixture of BA and DMF (Figure S1). We also observed that quenching of the photocatalyst also happens in the presence of imine. This result can explain the necessity of a higher loading of the photocatalyst (3.5 mol %) in our optimized conditions. Indeed, the presence of free imine, in constant equilibrium with iminium ion, can be involved in the reductive quenching of the photocatalyst (Leitch et al., 2020). An experiment of radical trapping *via* TEMPO confirmed the formation of alkyl (R) radical (detected by GC-MS) from BA precursor (Scheme S1). Finally, in the absence of light no product formation was detected, suggesting the fundamental role of light in the reaction mechanism (Figure S3).

**Figure 5:**
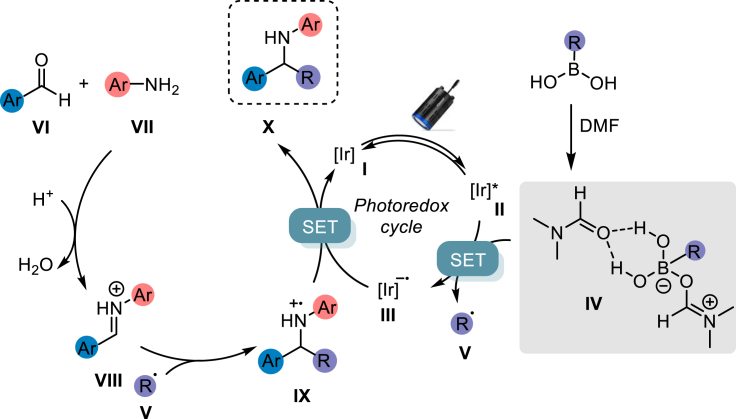
Proposed mechanism

In light of these results, the following mechanism has been proposed (Figure 5). First, the iridium photocatalyst undergoes excitation to **II** upon irradiation with blue light. A single electron transfer between **II** and the complex between BA and DMF **IV** leads to the formation of a radical species **V**. The latter can directly attack the iminium ion **VIII**, formed by condensation between aldehyde **VI** and aniline **VII**. The resulting species **IX** can undergo SET with the reduced form of the photocatalyst **III**, closing the photoredox cycle by regenerating the iridium species **I** and delivering the desired product **X**.

### Conclusions

In conclusion, a photoredox-catalyzed reaction manifold has been developed for the traditional multicomponent Petasis reaction, using BAs as an alkyl radical source. The oxidation potential of alkyl BAs was tuned by means of a hydrogen bond-assisted activation with DMF as solvent. The developed process was also successfully coupled with a continuous-flow reactor for easy scale up while decreasing the longer reaction times with improved yields. Further, the method shows good functional group tolerance and can deliver complex secondary amines starting from simple precursors, and it is amenable to the late-stage functionalization of biologically relevant molecules.

### Limitation of the study

The study is limited to primary and secondary alkyl BAs. ^13^CNMR spectra of compounds **4c** and **5e** contain traces in the aromatic region of an impurity derived from the benzaldehyde used as starting material. This could not be separated from the desired compound because of their close polarity. ^13^CNMR spectra of compounds **6c** and **6h** contain traces of grease resulting from hexane, used as solvent during the purification.

## STAR★Methods

### Key resources table

**Table:** 

REAGENT or RESOURCE	SOURCE	IDENTIFIER
**Chemicals, Peptides, and Recombinant Proteins**		

Cyclopentylboronic acid	Fluorochem	Cat#011025
p-Anisaldehyde	ACROS Organics	Cat#10010680
Aniline	ACROS Organics	Cat#10667512
N,N-Dimethylformamide	ACROS Organics	Cat#10534341
Acetonitrile	ACROS Organics	Cat#10222052
4CzIPN	Synthetized in our lab	https://doi.org/10.1039/C8CC02169D
[Ir{dF(CF_3_)ppy}_2_(dtbbpy)]PF_6_	Synthetized in our lab	https://doi.org/10.1038/nprot.2016.176

**Other**		

PR160 blue LED lamps (40W, peak wavelength of 456 nm)	Kessil	https://kessil.com/
3 pump easy-Photochem LED	Vapourtec	https://www.vapourtec.com/
Thin layer chromatography using TLC-Plates ALUGRAM Xtra SIL G/UV254	MACHEREY-NAGEL	https://www.mn-net.com/
Silica gel for chromatography, 0.060-0.200 mm, 60A	ACROS Organics	Cat#240370300
AV-300 (300 MHZ) spectrometer	Bruker	https://www.bruker.com/en.html
AV-III HD 400 (400 MHZ) spectrometer	Bruker	https://www.bruker.com/en.html
AV-II+600 (600 MHZ) spectrometer	Bruker	https://www.bruker.com/en.html
Fluorolog, HORIBA Instruments spectrophotometer	HORIBA	https://www.horiba.com/usa/
Metrohm PGSTAT204 potentiostat/galvanostat	Metrohm	https://www.metrohm.com/en

### Resource availability

#### Lead contact

Further information and requests for resources and reagents should be directed to and will be fulfilled by the lead contact, Upendra Kumar Sharma (upendrakumar.sharma@kuleuven.be).

#### Materials availability

All other data supporting the finding of this study are available within the article and the supplemental information or from the lead contact upon reasonable request.

### Method details

#### General optimization procedure

**Figure:**
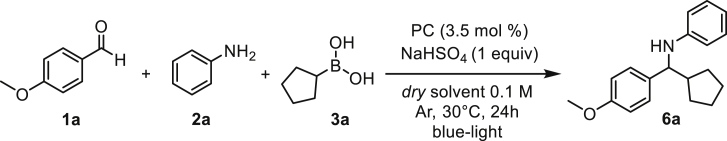

In a 4mL screw cap vial equipped with a stirring bar were added cyclopentylboronic acid (45.6 mg, 0.4 mmol, 2 equiv), NaHSO_4_ (27.6 mg, 0.2 mmol, 1 equiv) and the appropriate photocatalyst. The vial was sealed with a screw cap with silicone septum, and three cycles vacuum/argon were performed. Dry solvent was degassed for 10 minutes before being added to the vial. Aniline (0.027 mL, 0.3 mmol, 1.5 equiv) and *p*-anisaldehyde (0.024 mL, 0.2 mmol, 1 equiv) were added with a Hamilton glass syringe. The vial was then irradiated with blue light (2 × 40 W) under fan cooling to maintain a temperature of 30-35°C. After 24 hours, the reaction mixture was diluted with ethyl acetate (10 mL), transferred to a separatory funnel, and washed with water (10 mL) three times. Finally, the aqueous phase was extracted with ethyl acetate. The combined organic extracts were washed with brine, dried over Na_2_SO_4_ and concentrated in vacuo. Purification of the crude product was performed through flash chromatography column on silica gel using the indicated solvent system.

#### General procedure for Petasis reaction

To a 4ml screw cap vial equipped with a stirring bar were added boronic acid (0.8 mmol, 2 equiv), NaHSO_4_ (48 mg, 0.4 mmol, 1 equiv), [Ir{dF(CF_3_)ppy}_2_(dtbbpy)]PF_6_ (3.5 mol%, 13.2 mg), the appropriate amine (0.6 mmol, 1.5 equiv) and aldehyde (0.4 mmol, 1 equiv) if solid. The vial was sealed with a screw cap with silicone septum, and three cycles vacuum/argon were performed. Dry DMF was degassed for 10 minutes before being added to the vial. Amine and aldehyde were added if liquid with a Hamilton glass syringe at this point. The vial was then irradiated by blue-light under fan cooling to maintain a temperature of 30-35°C. After 24 hours, the reaction mixture was diluted with ethyl acetate (10 mL) and washed with water (10 mL) three times in a separatory funnel. Finally, the aqueous phase was extracted with ethyl acetate. The combined organic extracts were washed with brine, dried over Na_2_SO_4_ and concentrated in vacuo. Purification of the crude product was performed through flash chromatography column on silica gel using the indicated solvent system.

#### General procedure for Petasis reaction in flow

An oven-dried 10 mL glass vial was charged with alkyl boronic acid (3 equiv), photoredox catalyst ([Ir{dF(CF_3_)ppy}_2_(dtbbpy)]PF_6_, 3.5 mol%), *p*-anisaldehyde (1 equiv) and a mixture of ACN and DMF (1:3, 0.1 M). A second oven-dried 10 mL glass vial was equipped with aniline (1.5 equiv) and a mixture of ACN and DMF (1:3, 0.1 M). The vials were closed with a silicon septum and purged with argon three times. The resulting clear solutions were then pumped through a 10 mL volume reactor (Vapourtec E-series) irradiated with blu-light (450 nm) at 0.100 mL/min, keeping the temperature set at 30°C. Once the solutions had been fully taken up by the pumps, the input was changed to ACN/DMF solvent to push the reaction. The crude reaction mixture was collected in a round bottom flask and purified by chromatography column.

#### Characterization of compounds 4a- 4k

##### *N*-(cyclopentyl(4-iodophenyl)methyl)aniline (4a)

**Figure:**
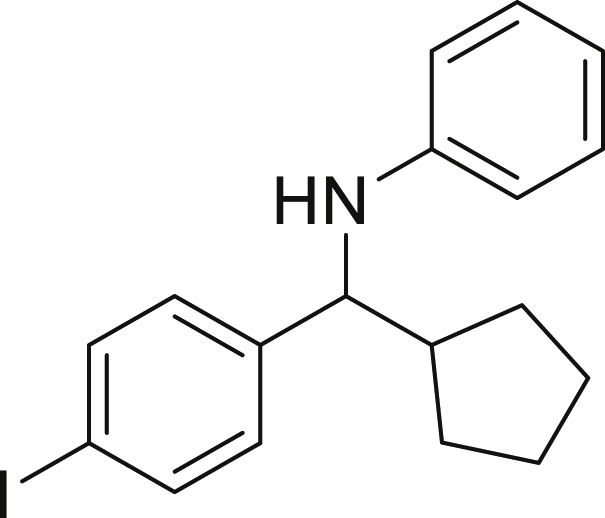

**4a** (95 mg, 63% yield) was prepared according to the general procedure. The desired amine 4a was isolated through flash column chromatography as a dark yellow liquid (eluent: Hept/EtOAc, 98:2).

^**1**^**H NMR** (300 MHz, Chloroform-d) δ 7.67 (d, J = 7.9 Hz, 2H), 7.19–7.08 (m, 4H), 6.68 (t, J = 7.4 Hz, 1H), 6.52 (d, J = 8.0 Hz, 2H), 4.18 (s, 1H), 4.09 (d, J = 8.3 Hz, 1H), 2.15 (m, 1H), 1.99–1.85 (m, 1H), 1.74–1.60 (m, 3H), 1.57–1.43 (m, 3H), 1.32 (m, 1H).

^**13**^**C NMR** (75 MHz, Chloroform-d) δ 147.31, 143.84, 137.43, 129.13, 129.08, 117.37, 113.30, 92.07, 62.65, 47.62, 30.11, 29.91, 25.24, 25.22.

**HRMS** (ESI^+^): [M + H] calculated for C_18_H_20_IN: 378.0715, found: 378.0701.

**IR** (neat, ν/cm^−1^) 3414, 3048, 2949, 2864, 1600, 1500, 746, 690.

##### *N*-((4-bromophenyl)(cyclopentyl)methyl)aniline (4b)

**Figure:**
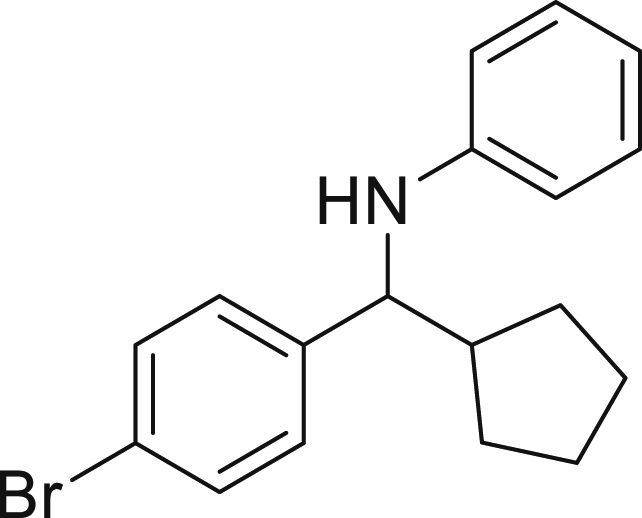

**4b** (88 mg, 67% yield) was prepared according to the general procedure. The desired amine **4b** was isolated through flash column chromatography as an orange liquid (eluent: Hept/EtOAc, 98:2).

^1^H NMR (300 MHz, Chloroform-*d*) δ 7.49–7.41 (m, 2H), 7.28–7.24 (m, 2H), 7.10 (t, *J* = 7.9 Hz, 1H), 6.66 (t, *J* = 7.3 Hz, 1H), 6.51 (d, *J* = 7.9 Hz, 2H), 4.18 (s, 1H), 4.09 (d, *J* = 8.3 Hz, 1H), 2.14 (m, 1H), 1.97–1.87 (m, 1H), 1.74–1.59 (m, 4H), 1.53–1.44 (m, 3H), 1.33-1.31 (m, 1H).

^13^C NMR (101 MHz, Chloroform-d) δ 147.39, 143.18, 131.55, 129.19, 128.81, 120.56, 117.43, 113.37, 62.65, 47.72, 30.16, 29.98, 25.30, 25.28.

**HRMS** (ESI^+^): [M-Br] calculated for C_18_H_20_BrN: 251.1674, found: 251.1530.

**IR (neat,** ν**/**cm^−1^**)** 3419, 3050, 2950, 2865, 1600, 1501, 1008, 867, 822, 690.

##### *N*-(cyclopentyl(4-fluorophenyl)methyl)aniline (4c)

**Figure:**
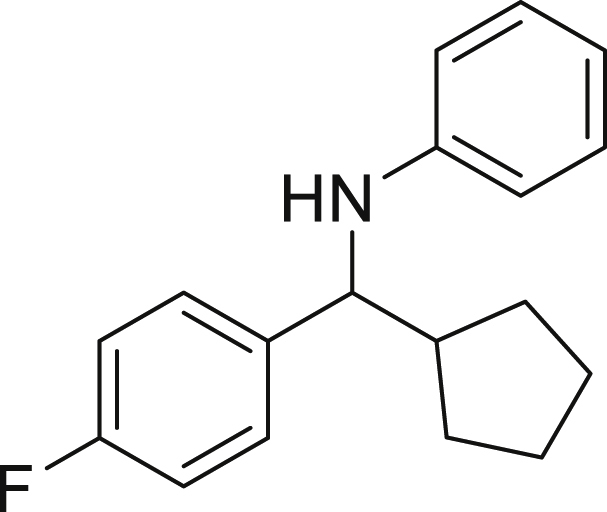

**4c** (78 mg, 72% yield) was prepared according to the general procedure. The desired amine **4c** was isolated through flash column chromatography as a dark yellow liquid (eluent: n-Hex/EtOAc, 97:3).

^1^H NMR (300 MHz, Chloroform-*d*) δ 7.32 (dd, *J* = 8.5, 5.7 Hz, 2H), 7.08 (t, *J* = 7.9 Hz, 2H), 7.00 (t, *J* = 8.7 Hz, 2H), 6.64 (t, *J* = 7.3 Hz, 1H), 6.50 (d, *J* = 7.9 Hz, 2H), 4.17 (s, 1H), 4.08 (d, *J* = 8.4 Hz, 1H), 2.24-2.04 (m, 1H), 1.98-1.82 (m, 1H), 1.68-1.58 (m, 3H), 1.55-1.42 (m, 3H), 1.34-1.24 (m, 1H).

^13^C NMR (101 MHz, Chloroform-d) δ 158.75, 150.12, 135.03, 127.94, 126.49 (d, J = 3.9 Hz), 113.24 (d, J = 145.1 Hz), 77.93–75.99 (m), 62.30, 55.31, 47.85, 30.17, 30.14, 25.34, 25.24. Traces of impurities at δ 132, 123, 118.

**HRMS** (ESI^+^): [M-H] calculated for C_18_H_20_FN: 268.1507, found: 268.1483.

**IR (neat,** ν**/**cm^−1^**)** 3417, 2951, 2866, 1600, 1501, 1218, 835, 746, 690, 508.

##### *N*-(cyclopentyl(4-(trifluoromethyl)phenyl)methyl)aniline (4d)

**Figure:**
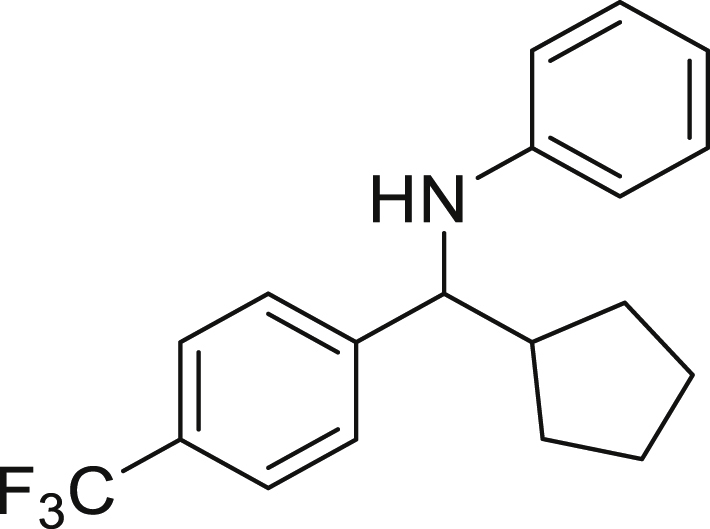

**4d** (80 mg, 63% yield) was prepared according to the general procedure. The desired amine **4d** was isolated through flash column chromatography as a dark yellow liquid (eluent: Hept/EtOAc, 98:2).

^1^H NMR (300 MHz, Chloroform-*d*) δ 7.58 (d, *J* = 8.1 Hz, 2H), 7.49 (d, *J* = 8.0 Hz, 2H), 7.09 (t, *J* = 7.7 Hz, 2H), 6.66 (t, *J* = 7.4 Hz, 1H), 6.49 (d, *J* = 7.9 Hz, 2H), 4.23–4.14 (m, 2H), 2.24-2.04 (m, 1H), 1.98-1.82 (m, 1H), 1.77–1.57 (m, 3H), 1.55-1.42 (m, 3H), 1.39–1.22 (m, 1H).

^13^C NMR (75 MHz, Chloroform-*d*) δ 148.39, 147.29, 129.27, 127.37, 125.52, 125.47, 117.60, 113.34, 62.87, 47.71, 30.21, 29.96, 25.29.

**HRMS** (ESI^+^): [M + H] calculated for C_19_H_20_F_3_N: 320.1620, found: 320.1625.

**IR (neat,** ν**/**cm^−1^**)** 3421, 2954, 2869, 1602, 1502, 1322, 1116, 1064, 749, 690, 506.

##### Methyl 4-(Cyclopentyl(phenylamino)methyl)benzoate (4e)

**Figure:**
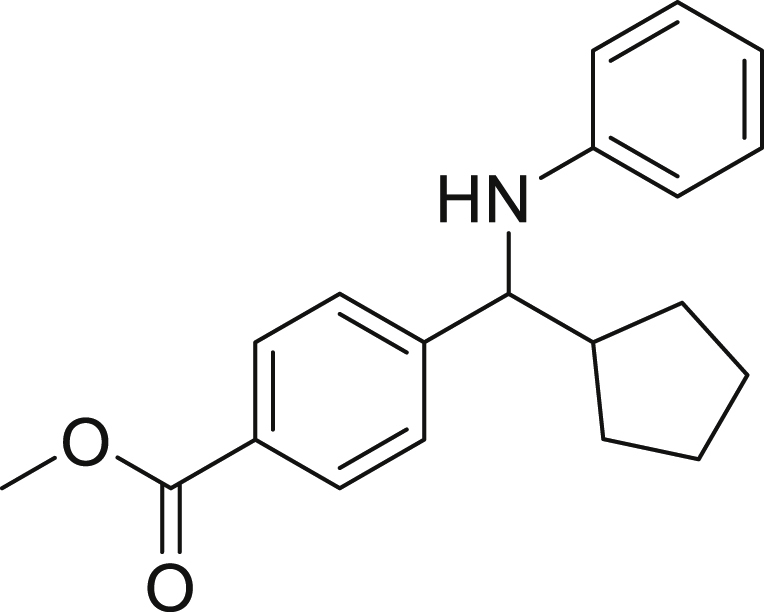

**4e** (72 mg, 58% yield) was prepared according to the general procedure. The desired amine **4e** was isolated through flash column chromatography (eluent: Hept/EtOAc, 98:2) as a dark yellow solid, m.p. 97.4°C.

^1^H NMR (300 MHz, Chloroform-d) δ 8.05–7.92 (m, 2H), 7.49–7.37 (m, 2H), 7.13–7.01 (m, 2H), 6.63 (t, J = 7.3 Hz, 1H), 6.48 (d, J = 7.9 Hz, 2H), 4.15 (d, J = 8.4 Hz, 1H), 3.90 (s, 3H), 2.27–2.08 (m, 1H), 1.98-1.82 (m, 1H), 1.73–1.58 (m, 3H), 1.51–1.39 (m, 3H), 1.33–1.25 (m, 1H).

^13^C NMR (75 MHz, Chloroform-*d*) δ 167.14, 149.68, 147.37, 129.86, 129.86, 129.18, 127.10, 117.47, 113.35, 63.02, 52.09, 47.60, 30.14, 29.98, 25.27.

**IR (neat,** ν**/**cm^−1^**)** 3353, 2948, 2869, 1701, 1280, 1112, 771, 691.

The spectral data is consistent with the literature data (Yi et al., 2019)**.**

##### *N*-(Cyclopentyl(6-methoxynaphthalen-2-yl)methyl)aniline (4f)

**Figure:**
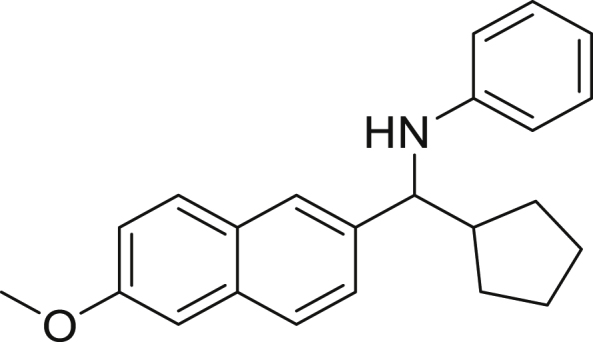

**4f** (70 mg, 53% yield) was prepared according to the general procedure. The desired amine **4f** was isolated through flash column chromatography (eluent: heptane/EtOAc, 97:3) as a crystalline yellow solid, m.p. 123°C.

^1^H NMR (300 MHz, Chloroform-*d*) δ 7.77–7.67 (m, 3H), 7.48 (dd, *J* = 8.4, 1.8 Hz, 1H), 7.18–7.11 (m, 2H), 7.06 (t, *J* = 7.7 Hz, 2H), 6.65–6.53 (m, 3H), 4.28 (s, 1H), 4.23 (d, *J* = 8.5 Hz, 1H), 3.93 (s, 3H), 2.32–2.20 (m,1H), 1.97–1.92 (m, 1H), 1.69–1.61 (m, 2H), 1.61–1.41 (m, 4H), 1.40–1.32 (m, 1H).

^13^C NMR (101 MHz, Chloroform-d) δ 157.49, 147.85, 139.33, 133.92, 129.40, 129.14, 128.97, 127.07, 125.83, 125.68, 118.78, 117.14, 113.42, 105.80, 63.31, 55.41, 47.88, 30.21 (d, J = 15.6 Hz), 25.36 (d, J = 6.7 Hz).

**HRMS** (ESI^+^): [M-H] calculated for C_23_H_25_NO: 330.1863, found: 330.1848.

**IR (neat,** ν**/**cm^−1^**)** 2924, 2854, 1723, 1620, 1503, 1264, 1170, 1030, 895, 853.

##### 2-(*tert*-butyl)-6-(cyclopentyl(phenylamino)methyl)phenol (4g)

**Figure:**
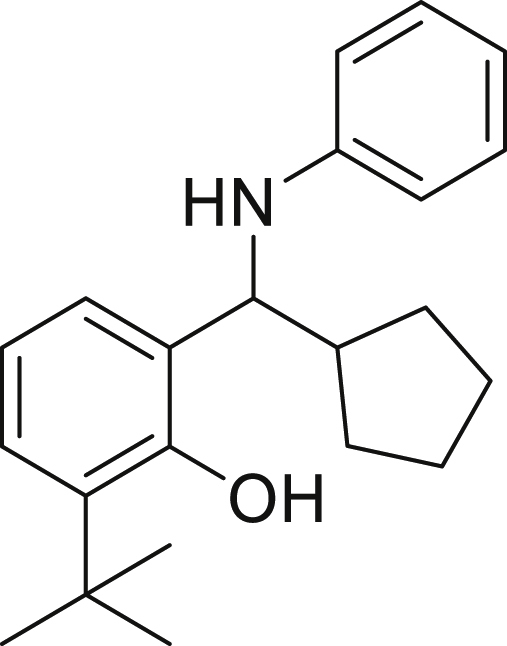

**4g** (60 mg, 46% yield) was prepared according to the general procedure. The desired ammine **4g** was isolated through flash column chromatography as a yellow liquid (eluent: Hept/EtOAc, 98:2).

^1^H NMR (300 MHz, Chloroform-*d*) δ 10.05 (s, 1H), 7.23–7.12 (m, 3H), 6.97–6.94 (m, 1H), 6.90–6.85 (m, 1H), 6.81–6.77 (m, 3H), 4.06–3.91 (m, 2H), 2.56–2.41 (m, 1H), 2.01–1.93 (m, 1H), 1.77–1.59 (m, 3H), 1.60–1.49 (m, 3H), 1.37 (s, 9H), 1.34–1.31 (m, 1H).

^13^C NMR (101 MHz, Chloroform-d) δ 156.15, 147.45, 137.31, 129.33, 127.20, 126.09, 125.67, 121.34, 118.54, 117.15, 68.22, 45.40, 34.87, 30.63, 30.23, 29.70, 25.21, 25.09.

**HRMS** (ESI^+^): [M + H] calculated for C_22_H_29_NO: 324.2321, found: 324.2314.

**IR (neat,** ν**/**cm^−1^**)** 2952, 2868, 1602, 1436, 1236, 749, 690, 492.

##### *N*-(benzo[d]thiazol-2-yl(cyclopentyl)methyl)aniline (4h)

**Figure:**
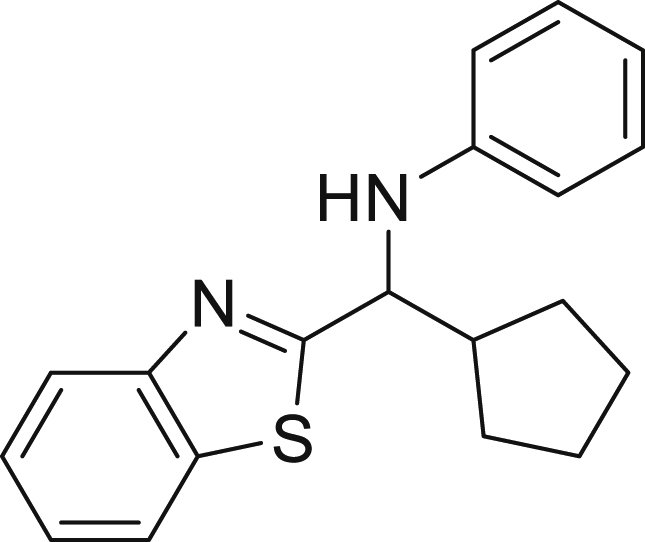

**4h** (122 mg, 99% yield) was prepared according to the general procedure. The desired amine **4h** was isolated through flash column chromatography (eluent: Hept/EtOAc, 97:3) as a dark yellow solid, m.p. 143.2°C.

^1^H NMR (300 MHz, Chloroform-*d*) δ 8.02 (d, *J* = 8.1 Hz, 1H), 7.81 (d, *J* = 7.9 Hz, 1H), 7.46 (t, *J* = 7.7 Hz, 1H), 7.34 (t, *J* = 7.6 Hz, 1H), 7.13 (t, *J* = 7.7 Hz, 2H), 6.74–6.65 (m, 3H), 4.64 (d, *J* = 8.1 Hz, 1H), 4.37 (s, 1H), 2.52–2.44 (m, 1H), 2.03–1.87 (m, 1H), 1.75–1.50 (m, 6H), 1.30–1.27 (m, 1H).

^13^C NMR (75 MHz, Chloroform-*d*) δ 178.04, 153.49, 146.92, 135.07, 129.38, 125.91, 124.89, 122.94, 121.95, 118.56, 113.54, 78.90–76.05 (m), 61.76, 46.53, 29.68 (d, *J* = 12.9 Hz), 25.51 (d, *J* = 4.5 Hz).

**HRMS** (ESI^+^): [M + H] calculated for C_19_H_20_N_2_S: 309.4510, found: 309.1422.

**IR (neat,** ν**/**cm^−1^**)** 3274, 2950, 2863, 1598, 1495, 1311, 757, 691.

##### *N*-(cyclopentyl(2,3-dihydrobenzofuran-5-yl)methyl)aniline (4i)

**Figure:**
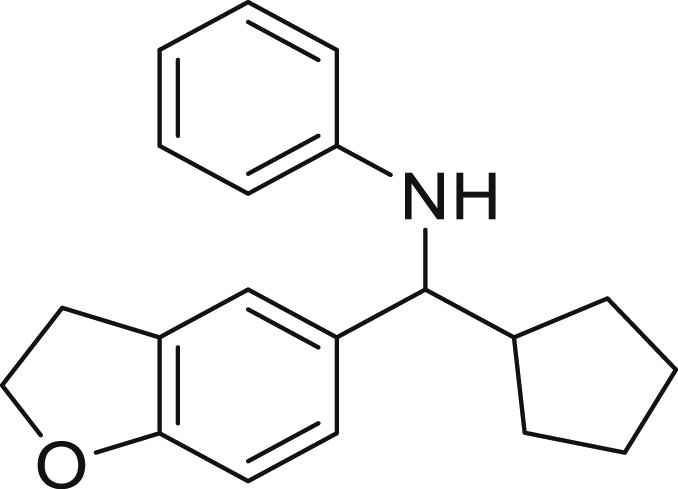

**4i** (66 mg, 56% yield) was prepared according to the general procedure. The desired amine **4i** was isolated through flash column chromatography (eluent: Hept/EtOAc, 97:3) as a yellow solid, m.p. 68.3°C.

^1^H NMR (300 MHz, Chloroform-*d*) δ 7.19 (s, 1H), 7.14–7.03 (m, 3H), 6.73 (d, *J* = 8.2 Hz, 1H), 6.63 (t, *J* = 7.3 Hz, 1H), 6.54 (d, *J* = 7.9 Hz, 2H), 4.54 (t, *J* = 8.7 Hz, 2H), 4.16 (s, 1H), 4.03 (d, *J* = 8.5 Hz, 1H), 3.17 (t, *J* = 8.7 Hz, 2H), 2.122–1.94 (m, 1H), 1.92–1.88 (m, 1H), 1.86–1.58 (m, 3H), 1.56–1.42 (m, 3H), 1.38–1.22 (m, 1H).

^13^C NMR (75 MHz, Chloroform-*d*) δ 159.09, 147.91, 136.17, 129.11, 127.09, 126.86, 123.23, 117.01, 113.37, 108.83, 71.27, 62.91, 48.16, 30.28, 30.13, 29.93, 25.38, 25.28.

**HRMS** (ESI^+^): [M-H] calculated for C_20_H_23_NO: 292.1707, found: 292.1706.

**IR (neat,** ν**/**cm^−1^**)** 3414, 2949, 2864, 1608, 1489, 1240, 1172, 1057, 1031, 806, 527.

##### *N*-(cyclopentyl(4-ethynylphenyl)methyl)aniline (4j)

**Figure:**
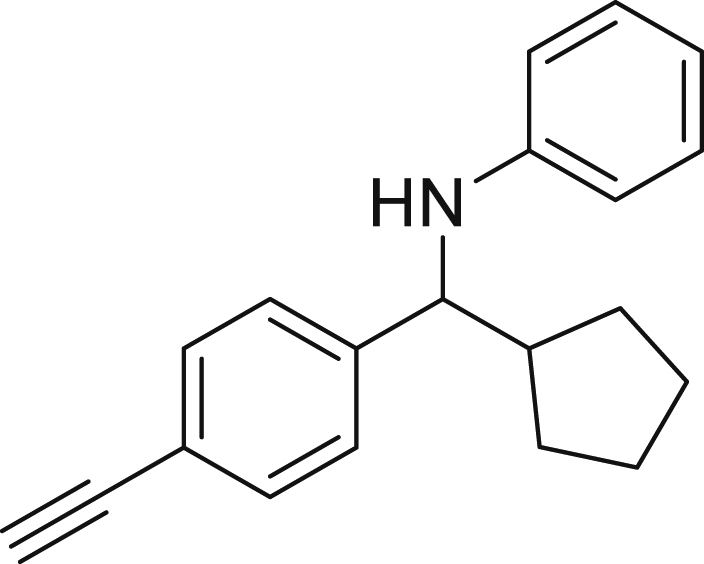

**4j** (20 mg, 18% yield) was prepared according to the general procedure. The desired amine **4j** was isolated through flash column chromatography (eluent: Hept/EtOAc, 98:2) as a white crystalline solid, m.p. 96°C.

^1^H NMR (400 MHz, Chloroform-d) δ 7.48–7.37 (m, 2H), 7.37–7.27 (m, 2H), 7.15–6.98 (m, 2H), 6.71–6.55 (m, 1H), 6.54–6.44 (m, 2H), 4.08 (d, J = 8.3 Hz, 1H), 3.03 (s, 1H), 2.22–2.09 (m, 1H), 1.99–1.86 (m, 1H), 1.70–1.54 (m, 4H), 1.48–1.39 (m, 2H), 1.30–1.22 (m, 1H).

^13^C NMR (151 MHz, Chloroform-d) δ 147.48, 145.18, 132.36, 129.21, 127.10, 120.63, 117.42, 113.39, 83.85, 63.01, 47.71, 30.18, 30.01, 25.32.

**IR (neat,** ν**/**cm^−1^**)** 3413, 3294, 2952, 2867, 1600, 1427, 1318, 1296, 1260, 908, 839, 732, 691.

##### *N*-(cyclopentyl(4-(ethenyloxy)phenyl)methyl)aniline (4k)

**Figure:**
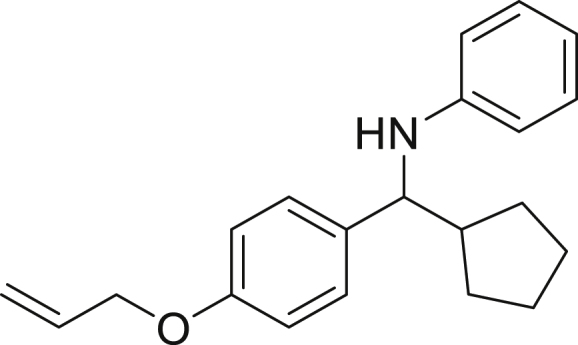

**4k** (66 mg, 54% yield) was prepared according to the general procedure. The desired amine **4k** was isolated through flash column chromatography (eluent: Hept/EtOAc, 98:2) as a yellow oil.

^1^H NMR (400 MHz, Chloroform-d) δ 7.28–7.22 (m, 2H), 7.11–7.02 (m, 2H), 6.88–6.82 (m, 2H), 6.65–6.57 (m, 1H), 6.53–6.48 (m, 2H), 6.12–5.98 (m, 1H), 5.41 (dq, J = 17.2, 1.6 Hz, 1H), 5.28 (dq, J = 10.5, 1.4 Hz, 1H), 4.53–4.45 (m, 2H), 4.17–4.13 (m, 1H), 4.04 (d, J = 8.5 Hz, 0H), 2.22–2.09 (m, 1H), 1.96–1.82 (m, 1H), 1.72–1.57 (m, 3H), 1.53–1.36 (m, 3H), 1.34–1.20 (m, 1H).

^13^C NMR (101 MHz, Chloroform-d) δ 157.59, 147.83, 136.28, 133.60, 129.13, 128.01, 117.69, 117.05, 114.60, 113.39, 68.94, 62.59, 48.01, 30.22, 30.10, 25.38, 25.31.

**IR (neat,** ν**/**cm^−1^**)** 3407, 3054, 2956, 2944, 2936, 2866, 1601, 1505, 1230, 1011, 830, 743, 509.

#### Characterization of compounds 5a-5m

##### *N*-(cyclopentyl(4-methoxyphenyl)methyl)-4-iodoaniline (5a)

**Figure:**
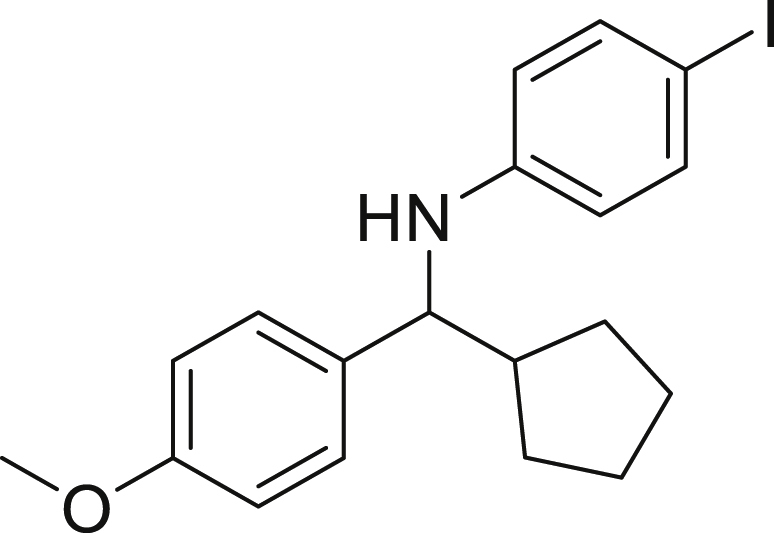

**5a** (117 mg, 71% yield) was prepared according to the general procedure. The desired amine **5a** was isolated through flash column chromatography as a yellow oil (eluent: heptane/EtOAc, 98:2).

^1^H NMR ^1^H NMR (400 MHz, Chloroform-*d*) δ 7.34–7.29 (m, 1H), 7.26–7.20 (m, 1H), 6.89–6.82 (m, 1H), 6.34–6.28 (m, 1H), 4.22 (s, 1H), 4.00 (d, *J* = 8.4 Hz, 1H), 3.80 (s, 3H), 2.22–2.09 (m, 1H), 1.95–1.84 (m, 1H), 1.72–1.60 (m, 3H), 1.54–1.37 (m, 3H), 1.33–1.25 (m, 1H).

^13^C NMR (101 MHz, Chloroform-*d*) δ 158.64, 147.26, 137.66, 135.34, 127.95, 115.68, 113.89, 62.47, 55.33, 47.89, 30.17, 30.12, 25.36, 25.27.

**HRMS** (ESI^+^): [M-I] calculated for C_19_H_22_INO: 280.1701, found: 280.1698.

**IR (neat,** ν**/**cm^−1^**)** 3414, 2949, 2864, 1608, 1489, 1240, 1172, 1057, 1031, 806, 527.

##### *N*-(cyclopentyl(4-methoxyphenyl)methyl)-4-bromoaniline (5b)

**Figure:**
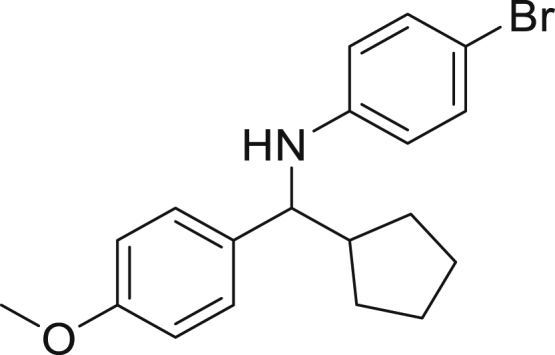

**5b** (102 mg, 71% yield) was prepared according to the general procedure. The desired amine **5b** was isolated through flash column chromatography as a dark yellow oil (eluent: heptane/EtOAc, 98:2).

^1^H NMR (300 MHz, Chloroform-*d*) δ 7.23 (d, *J* = 8.6 Hz, 2H), 7.14 (d, *J* = 8.7 Hz, 2H), 6.85 (d, *J* = 8.5 Hz, 2H), 6.45–6.33 (m, 2H), 4.20 (s, 1H), 4.00 (d, *J* = 8.4 Hz, 1H), 3.79 (s, 3H), 2.21–2.07 (m, 1H), 1.93–1.84 (m, 1H), 1.71–1.56 (m, 3H), 1.53–1.38 (m, 3H), 1.30–1.26 (m, 1H).

^13^C NMR (75 MHz, Chloroform-*d*) δ 158.63, 146.70, 135.37, 131.77, 127.95, 115.00, 113.88, 108.63, 62.62, 55.29, 47.89, 30.15, 30.11, 25.35, 25.25.

**HRMS** (ESI^+^): [M + H] calculated for C_19_H_22_BrNO: 360.0958, found: 360.0768.

**IR (neat,** ν**/**cm^−1^**)** 3418, 2950, 2865, 1592, 1508, 1241, 1173, 1031, 809.

##### *N*-(cyclopentyl(4-methoxyphenyl)methyl)-4-chloroaniline (5c)

**Figure:**
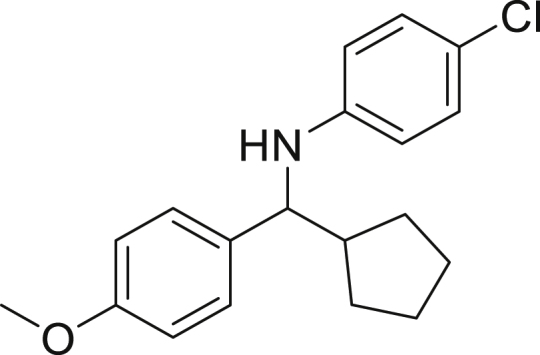

**5c** (83 mg, 65%), was prepared according to the general procedure. The desired amine **5c** was isolated through flash column chromatography as an orange oil (eluent: heptane/EtOAc, 98:2).

^1^H NMR (400 MHz, Chloroform-*d*) δ 7.28–7.21 (m, 2H), 7.05–6.99 (m, 2H), 6.89–6.83 (m, 2H), 6.48–6.38 (m, 2H), 4.19 (s, 1H), 4.01 (d, *J* = 8.5 Hz, 1H), 3.80 (s, 1H), 2.15 (dtd, *J* = 16.1, 8.9, 7.4 Hz, 1H), 1.97–1.84 (m, 1H), 1.73–1.60 (m, 3H), 1.55–1.39 (m, 3H), 1.35–1.23 (m, 1H).

^13^C NMR (101 MHz, Chloroform-*d*) δ 158.52, 146.19, 135.36, 128.82, 127.86, 121.51, 114.37, 113.77, 62.63, 55.21, 47.82, 30.1, 30.0, 25.2, 25.1.

**HRMS** (ESI^+^): [M + H] calculated for C_19_H_22_ClNO: 316.1462, found: 316.1290.

**IR (neat,** ν**/**cm^−1^**)** 3419, 2951, 2866, 1598, 1494, 1242, 1173, 1032, 811.

##### *N*-(cyclopentyl(4-methoxyphenyl)methyl)-4-fluoroaniline (5d)

**Figure:**
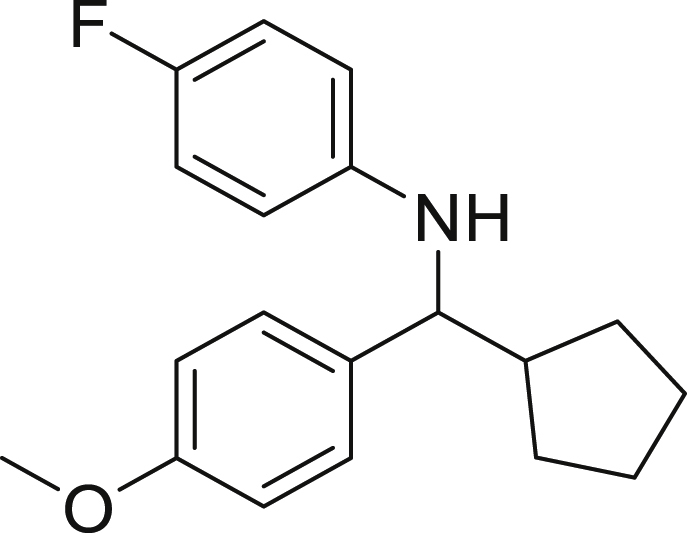

**5d** (68 mg, 57%), was prepared according to the general procedure. The desired amine **5d** was isolated through flash column chromatography as a yellow oil (eluent: heptane/EtOAc, 98:2).

^1^H NMR (300 MHz, Chloroform-*d*) δ 7.26 (m, 2H), 6.90–6.83 (m, 2H), 6.78 (t, *J* = 8.8 Hz, 2H), 6.44 (m, 2H), 4.06 (s, 1H), 3.98 (d, *J* = 8.5 Hz, 1H), 3.80 (s, 3H), 2.24–2.04 (m, 1H), 1.97–1.85 (m, 1H), 1.71–1.58 (m, 3H), 1.55–1.38 (m, 3H), 1.34–1.22 (m, 1H).

^13^C NMR (75 MHz, Chloroform-*d*) δ 158.46, 144.05, 135.70, 127.90, 115.53, 115.24, 114.04, 113.72, 63.19, 55.19, 47.90, 30.1, 30.0, 25.2, 25.1.

**HRMS** (ESI^+^): [M-H] calculated for C_19_H_22_FNO: 298.1612, found: 298.1598.

**IR (neat,** ν**/**cm^−1^**)** 3420, 2951, 2866, 1609, 1505, 1243, 816, 538, 508.

##### *N*-(cyclopentyl(4-methoxyphenyl)methyl)-4-(trifluoromethyl)-aniline (5e)

**Figure:**
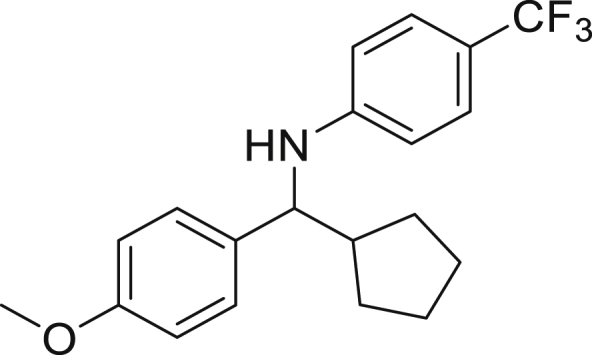

**5e** (70 mg, 50%), was prepared according to the general procedure. The desired amine **5e** was isolated through flash column chromatography as a yellow oil (eluent: heptane/EtOAc, 98:2).

^1^H NMR (400 MHz, Chloroform-*d*) 7.31–7.27 (m, 2H), 7.25–7.21 (m, 2H), 6.88–6.82 (m, 2H), 6.54–6.48 (m, 2H), 4.50 (s, 1H), 4.07 (d, *J* = 8.5 Hz, 1H), 3.79 (s, 3H), 2.21–2.08 (m, 1H), 1.96–1.85 (m, 1H), 1.71–1.58 (m, 4H), 1.52–1.39 (m, 3H).

^13^C NMR (101 MHz, Chloroform-d) δ 158.75, 150.12, 135.03, 127.94, 126.47, 113.96, 112.52, 62.30, 55.31, 47.85, 30.17, 30.14, 25.34, 25.24. Traces of impurities at δ 132, 123, 118.

**HRMS** (ESI^+^): [M-H] calculated for C_20_H_22_F_3_NO: 348.1580, found: 348.1567.

**IR (neat,** ν**/**cm^−1^**)** 3420, 2953, 2867, 1665, 1318, 1104, 822.

##### *N*-(cyclopentyl(4-methoxyphenyl)methyl)-4-methylaniline (5f)

**Figure:**
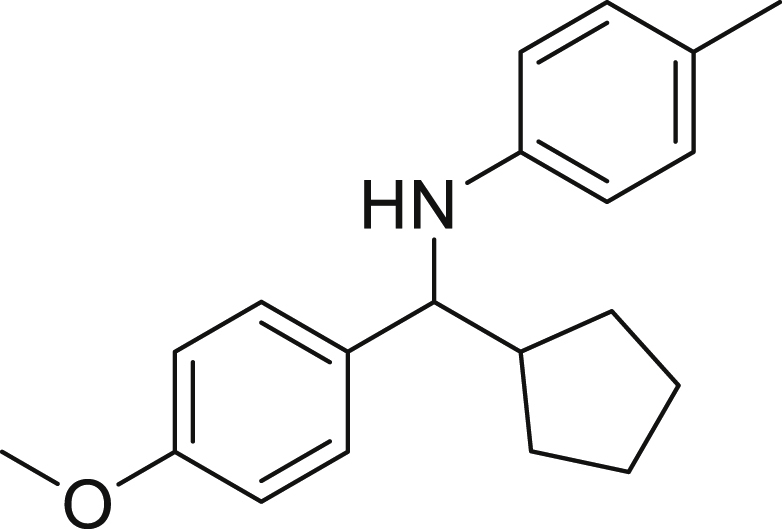

**5f** (81 mg, 69%), was prepared according to the general procedure. The desired amine **5f** was isolated through flash column chromatography as a yellow oil (eluent: heptane/EtOAc, 98:2)

^1^H NMR (300 MHz, Chloroform-*d*) δ 7.38–7.26 (m, 2H), 7.03–6.84 (m, 4H), 6.49 (d, *J* = 8.0 Hz, 2H), 4.07 (d, *J* = 8.3 Hz, 1H), 3.82 (s, 3H), 2.23–2.15 (m, 4H), 1.98–1.90 (m, 1H), 1.72–1.64 (m, 3H), 1.55–1.47 (m, 3H), 1.36–1.34 (m, 1H).

^13^C NMR (75 MHz, Chloroform-*d*) δ 158.45, 145.58, 136.26, 129.60, 128.00, 126.12, 113.76, 113.51, 62.83, 55.25, 48.00, 30.20, 30.08, 25.38, 25.30, 20.41.

**HRMS** (ESI^+^): [M-H] calculated for C_20_H_25_NO: 294.1863, found: 294.1851.

**IR (neat,** ν**/**cm^−1^**)** 3416, 2950, 2864, 1612, 1509, 1241, 1032.

##### 4-(*tert*-butyl)-N-(cyclopentyl(4-methoxyphenyl)methyl)aniline (5g)

**Figure:**
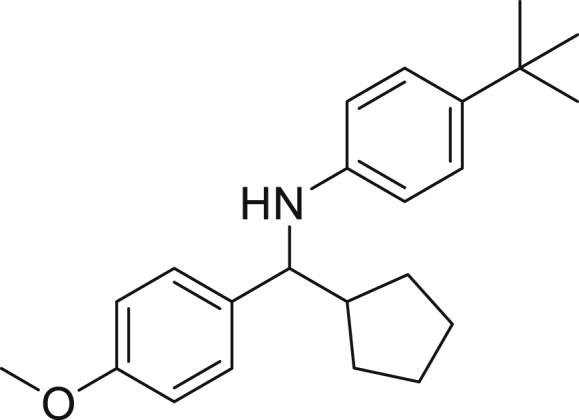

**5g** (174 mg, 67%), was prepared according to the general procedure. The desired amine **5g** was isolated through flash column chromatography (eluent: heptane/EtOAc, 98:2) as a yellow solid, m.p. 92.5°C.

^1^H NMR (300 MHz, Chloroform-d) δ 7.30 (d, J = 8.6 Hz, 2H), 7.12 (d, J = 8.5 Hz, 2H), 6.91–6.81 (m, 2H), 6.49 (d, J = 8.5 Hz, 2H), 4.03 (d, J = 8.4 Hz, 1H), 3.80 (s, 3H), 2.20–2.09 (m, 1H), 1.93–1.85 (m, 1H), 1.67–1.57 (m, 3H), 1.52–1.42 (m, 3H), 1.32–1.29 (m, 1H), 1.25 (s, 9H).

^13^C NMR (75 MHz, Chloroform-*d*) δ 158.50, 145.52, 139.71, 136.45, 128.06, 125.91, 113.78, 113.03, 62.90, 55.29, 48.08, 33.87, 31.64, 30.032, 30.09, 25.38, 25.29.

**HRMS** (ESI^+^): [M-H] calculated for C_23_H_31_NO: 336.2332, found: 336.2318.

**IR (neat,** ν**/**cm^−1^**)** 3402, 2955, 2864, 1609, 1509, 1241, 817, 545.

##### *N*-(cyclopentyl(4-methoxyphenyl)methyl)-4-ethylaniline (5h)

**Figure:**
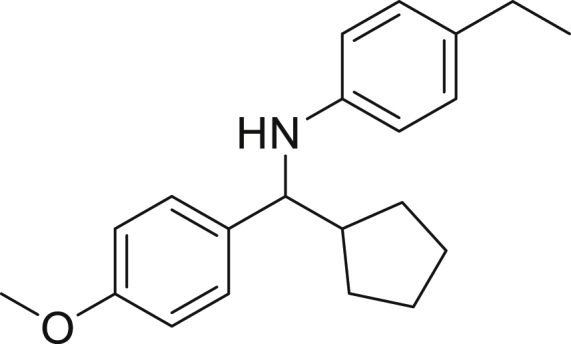

**5h** (118 mg, 95%), was prepared according to the general procedure. The desired amine **5h** was isolated through flash column chromatography as a yellow oil (eluent: heptane/EtOAc, 98:2).

^1^H NMR (300 MHz, Chloroform-d) δ 7.39–7.25 (m, 2H), 6.97 (d, J = 8.1 Hz, 2H), 6.90 (d, J = 8.2 Hz, 2H), 6.53 (d, J = 8.0 Hz, 2H), 4.08 (d, J = 8.5 Hz, 1H), 3.82 (s, 3H), 2.55 (q, J = 7.6 Hz, 2H), 2.24–2.13 (m, 1H), 1.71–1.64 (m, 1H), 1.57–1.47 (m, 3H), 1.57–1.47 (m, 3H), 1.37–1.31 (m, 3H), 1.21 (t, J = 7.6 Hz, 3H).

^13^C NMR (75 MHz, Chloroform-d) δ 158.46, 145.72, 136.25, 132.81, 128.10, 128.01, 113.74, 113.50, 62.88, 55.22, 47.99, 30.20, 30.07, 27.95, 25.36, 25.28, 15.95.

**HRMS** (ESI^+^): [M-H] calculated for C_21_H_27_NO: 308.2020, found: 308.2014.

**IR (neat,** ν**/**cm^−1^**)** 3402, 2954, 2866, 1612, 1509, 1241, 1032, 817, 546.

##### *N*-(cyclopentyl(4-methoxyphenyl)methyl)-2-fluoroaniline (5i)

**Figure:**
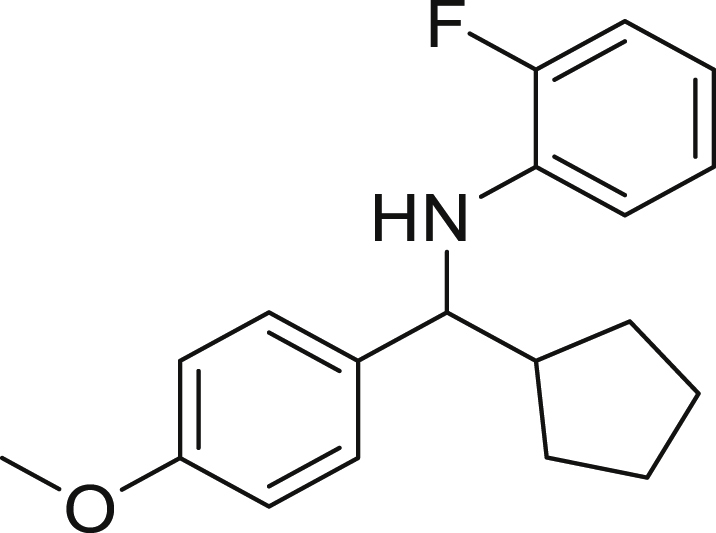

**5i** (65 mg, 54%), was prepared according to the general procedure. The desired amine **5i** was isolated through flash column chromatography as a yellow oil (eluent: heptane/EtOAc, 98:2).

^1^H NMR (300 MHz, Chloroform-d) δ 7.38–7.21 (m, 2H), 7.06–6.73 (m, 4H), 6.64–6.41 (m, 2H), 4.45 (s, 1H), 4.08 (d, J = 8.5 Hz, 1H), 3.81 (d, J = 2.1 Hz, 3H), 2.26–2.15 (m, 1H), 2.00–1.87 (m, 1H), 1.78–1.59 (m, 3H), 1.54–1.45 (m, 3H), 1.35–1.29 (m, 1H).

^13^C NMR (75 MHz, Chloroform-*d*) δ 158.64, 136.34, 136.19, 135.65, 127.96, 124.47, 116.30, 116.20, 114.32, 114.08, 113.86, 113.25, 62.45, 55.30, 47.99, 30.24, 30.14, 25.38, 25.28.

**HRMS** (ESI^+^): [M-H] calculated for C_19_H_22_FNO: 298.1612, found: 298.1607.

**IR (neat,** ν**/**cm^−1^**)** 3437, 2951, 2867, 1618, 1508, 1243, 1033, 826, 737, 552.

##### *N*-(cyclopentyl(4-methoxyphenyl)methyl)-3-iodoaniline (5j)

**Figure:**
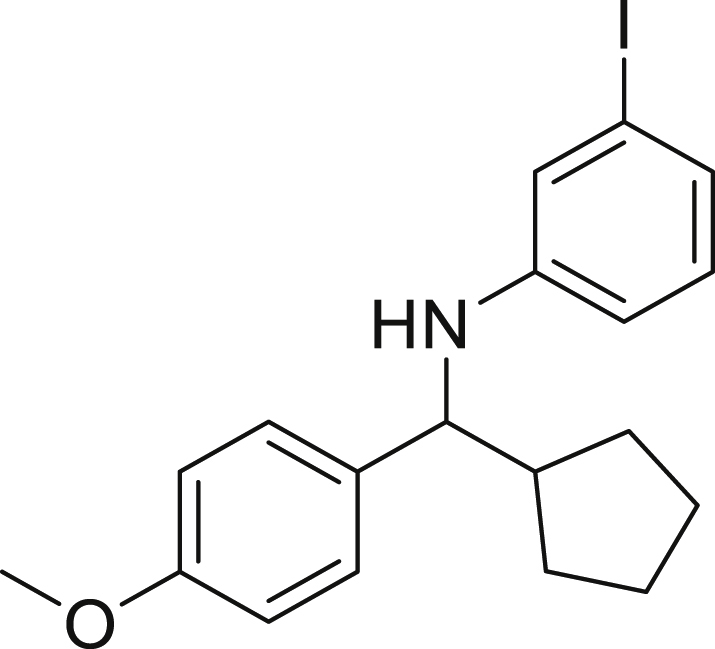

**5j** (86 mg, 53%), was prepared according to the general procedure. The desired amine **5j** was isolated through flash column chromatography as a yellow oil (eluent: heptane/EtOAc, 98:2).

^1^H NMR (300 MHz, Chloroform-*d*) δ 7.22 (d, *J* = 8.3 Hz, 2H), 6.97–6.81 (m, 4H), 6.75 (t, *J* = 7.9 Hz, 1H), 6.45–6.41 (m, 1H), 4.18 (s, 1H), 3.99 (d, *J* = 8.5 Hz, 1H), 3.79 (s, 3H), 2.17–2.06 (m, 1H), 1.92–1.82 (m, 1H), 1.72–1.55 (m, 3H), 1.52–1.38 (m, 3H), 1.35–1.21 (m, 1H).

^13^C NMR (75 MHz, Chloroform-d) δ 158.65, 148.95, 135.31, 130.58, 127.94, 125.91, 122.24, 113.91, 112.39, 95.11, 62.35, 55.31, 47.86, 30.19, 30.11, 25.37, 25.27.

**HRMS** (ESI^+^): [M-H] calculated for C_19_H_22_INO: 406.0675, found: 406.0662.

**IR (neat,** ν**/**cm^−1^**)** 3416, 2951, 2865, 1587, 1492, 1241, 728.

##### Methyl 4-(cyclopentyl(methyl(phenyl)amino)methyl)benzoate (5k)

**Figure:**
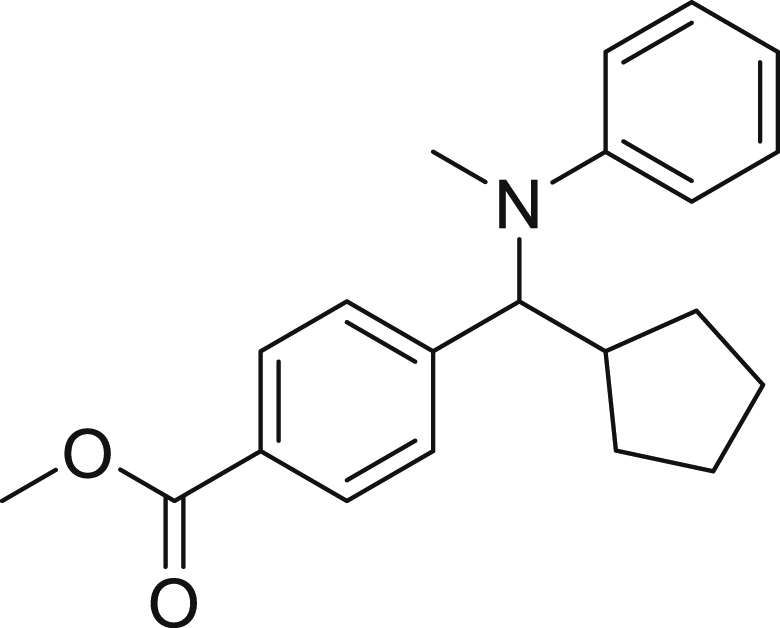

**5k** (37 mg, 31%), was prepared according to the general procedure. The desired amine **5k** was isolated through flash column chromatography (eluent: heptane/EtOAc, 98:2) as a yellow oil.

^1^H NMR (400 MHz, Chloroform-d) δ 7.31–7.19 (m, 4H), 6.93–6.87 (m, 2H), 6.87–6.81 (m, 2H), 6.76–6.69 (m, 1H), 4.64 (d, J = 11.0 Hz, 1H), 3.81 (s, 3H), 2.67 (s, 3H), 1.90–1.79 (m, 1H), 1.78–1.55 (m, 1H), 1.54–1.43 (m, 1H), 1.26–1.14 (m, 1H).

^13^C NMR (101 MHz, Chloroform-d) δ 158.53, 150.89, 133.19, 129.23, 129.05, 116.48, 113.49, 113.36, 66.95, 55.28, 41.35, 31.53, 31.44, 30.90, 25.99, 25.79.

**IR (neat,** ν**/**cm^−1^**)** 2950, 2864, 1596, 1502, 1452, 1305, 1245, 1177, 1177, 1033, 926, 781, 745, 690, 563.

##### Ethyl 4-((cyclopentyl(4-methoxyphenyl)methyl)amino)benzoate (5l)

**Figure:**
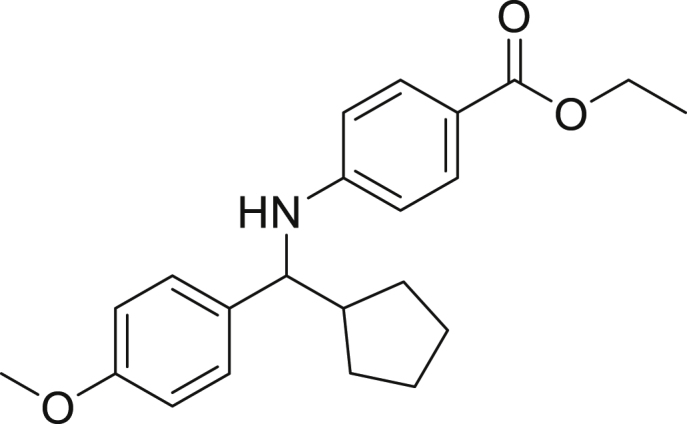

**5l** (119 mg, 84%), was prepared according to the general procedure. The desired amine **5l** was isolated through flash column chromatography as a yellow oil (eluent: heptane/EtOAc, 97:3).

^1^H NMR (300 MHz, Chloroform-*d*) δ 7.83–7.69 (m, 2H), 7.25–7.16 (m, 2H), 6.92–6.75 (m, 2H), 6.59–6.41 (m, 2H), 4.63 (s, 1H), 4.27 (q, J = 7.1 Hz, 2H), 4.10 (d, J = 8.0 Hz, 1H), 3.77 (s, 3H), 2.16 (q, J = 8.1 Hz, 1H), 1.92–1.85 (m, 1H), 1.66–1.56 (m, 3H), 1.49–1.40 (m, 3H), 1.30–1.21 (m, 4H).

^13^C NMR (75 MHz, Chloroform-*d*) δ 166.93, 158.70, 151.34, 135.02, 131.36, 127.93, 118.56, 113.93, 112.26, 62.12, 60.16, 55.29, 47.77, 30.15, 30.12, 25.33, 25.23.

**HRMS** (ESI^+^): [M + H] calculated for C_22_H_27_NO_3_: 354.2063, found: 354.2059.

**IR (neat,** ν**/**cm^−1^**)** 3375, 2953, 2867, 1683, 1600, 1268, 1169, 832, 769.

##### Butyl 4-((cyclopentyl(4-methoxyphenyl)methyl)amino)benzoato (5m)

**Figure:**
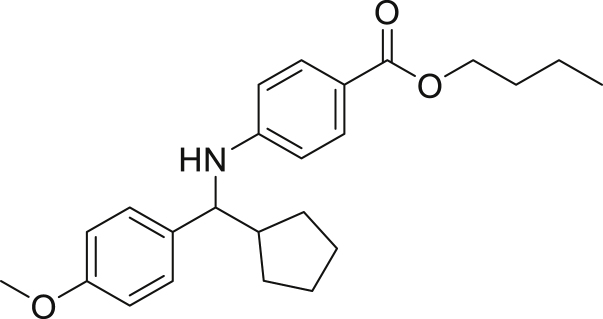

**5m** (109 mg, 71%), was prepared according to the general procedure. The desired amine **5m** was isolated through flash column chromatography as a yellow oil (eluent: heptane/EtOAc, 97:3).

^1^H NMR (300 MHz, Chloroform-d) δ 7.79–7.74 (m, 2H), 7.24–7.19 (m, 2H), 6.88–6.76 (m, 2H), 6.53–6.41 (m, 2H), 4.63 (s, 1H), 4.22 (t, J = 6.5 Hz, 2H), 4.15–4.06 (m, 1H), 3.77 (s, 3H), 2.20–2.15 (m, 1H), 1.92–1.84 (m, 1H), 1.73–1.56 (m, 5H), 1.50–1.40 (m, 5H), 0.92 (m, 4H).

^13^C NMR (75 MHz, Chloroform-d) δ 167.00, 158.70, 151.34, 135.02, 131.37, 127.93, 118.59, 113.93, 112.25, 64.08, 62.14, 55.29, 47.77, 32.00, 31.03, 30.15, 30.13, 25.28, 22.81, 19.41, 14.23, 13.88.

**HRMS** (ESI^+^): [M + H] calculated for C_24_H_31_NO_3_: 382.2376, found: 382.2373.

**IR (neat,** ν**/**cm^−1^**)** 3370, 2961, 2873, 1683, 1303, 834, 530.

#### Characterization of compounds 6a-6k

##### *N*-(cyclopentyl(4-methoxyphenyl)methyl)aniline (6a)

**Figure:**
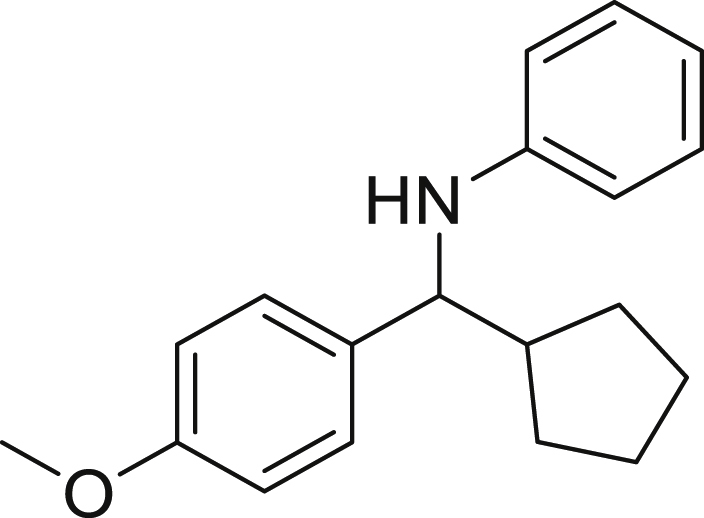

**6a** (85.5 mg, 76%), was prepared according to the general procedure. The desired amine **6a** was isolated through flash column chromatography (eluent: heptane/EtOAc, 98:2) as a yellow oil.

^1^H NMR (400 MHz, Chloroform-d) δ 7.29–7.23 (m, 2H), 7.10–7.03 (m, 2H), 6.87–6.81 (m, 2H), 6.65–6.58 (m, 1H), 6.54–6.47 (m, 2H), 4.15 (s, 1H), 4.04 (d, J = 8.4 Hz, 1H), 3.78 (s, 3H), 2.21–2.08 (m, 1H), 1.89 (dtd, J = 11.9, 7.4, 3.4 Hz, 1H), 1.70–1.56 (m, 3H), 1.52–1.40 (m, 3H), 1.32–1.24 (m, 1H).

^13^C NMR (101 MHz, Chloroform-d) δ 158.52, 147.84, 136.10, 129.13, 128.02, 117.06, 113.81, 113.41, 62.59, 55.32, 48.03, 30.23, 30.11, 25.39, 25.31.

**HRMS** (ESI^+^): [M-H] calculated for C_19_H_23_NO: 280.1707, found: 280.1702.

**IR (neat,** ν**/**cm^−1^**)** 3401, 2955, 2864, 1601, 1503, 1239, 1061, 749, 510.

##### *N*-(cyclobutyl(4-methoxyphenyl)methyl)aniline (6b)

**Figure:**
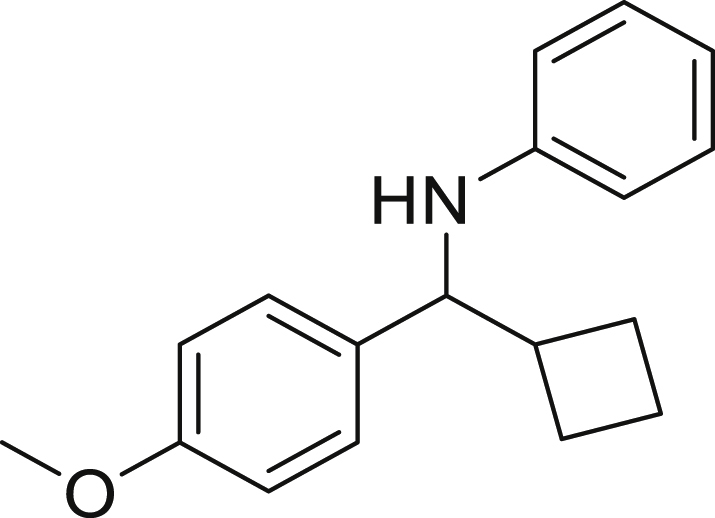

**6b** (69 mg, 76%), was prepared according to the general procedure. The desired amine **6b** was isolated through flash column chromatography (eluent: heptane/EtOAc, 98:2) as a yellow solid, m.p. 27°C.

^1^H NMR (300 MHz, Chloroform-d) δ 7.35–7.22 (m, 2H), 7.09 (t, J = 7.6 Hz, 2H), 6.95–6.81 (m, 2H), 6.65 (t, J = 7.3 Hz, 1H), 6.53 (d, J = 8.0 Hz, 1H), 4.15 (d, J = 9.1 Hz, 1H), 4.00 (s, 1H), 3.81 (s, 3H), 2.59–2.48 (m, 1H), 2.25–2.08 (m, 1H), 2.01–1.73 (m, 5H).

^13^C NMR (75 MHz, Chloroform-d) δ 158.63, 147.90, 134.66, 129.13, 127.66, 117.21, 113.91, 113.53, 63.29, 55.31, 42.78, 26.21, 25.59, 17.64.

**HRMS** (ESI^+^): [M-H] calculated for C_18_H_21_NO: 266.1550, found: 266.1541.

**IR (neat,** ν**/**cm^−1^**)** 3416, 3011, 2966, 1581, 1172, 752, 505.

##### *N*-(cyclohexyl(4-methoxyphenyl)methyl)aniline 6c

**Figure:**
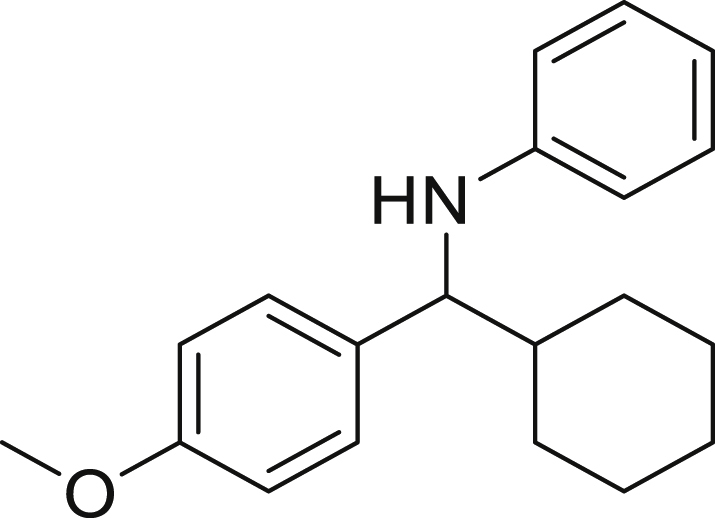

**6c** (71 mg, 60%), was prepared according to the general procedure. The desired amine **6c** was isolated through flash column chromatography as a yellow oil (eluent: hexane/EtOAc, 98:2).

^1^H NMR ^1^H NMR (400 MHz, Chloroform-*d*) δ 7.23–7.17 (m, 2H), 7.12–7.03 (m, 2H), 6.87–6.80 (m, 2H), 6.63–6.58 (m, 1H), 6.54–6.48 (m, 2H), 4.11 (s, 1H), 4.07 (d, *J* = 6.2 Hz, 1H), 3.78 (s, 3H), 1.92–1.87 (m, 1H), 1.78–1.74 (m, 2H), 1.70–1.52 (m, 3H), 1.34–0.99 (m, 5H).

^13^C NMR (101 MHz, Chloroform-d) δ 158.49, 147.97, 134.74, 129.15, 128.29, 116.97, 113.69, 113.30, 62.91, 55.31, 45.13, 30.26, 29.71, 27.58, 26.53, 26.48. Traces of hexane at δ 31.64, 22.70, 14.14.

**HRMS** (ESI^+^): [M-H] calculated for C_19_H_23_NO: 280.1707, found: 280.1702.

**IR (neat,** ν**/**cm^−1^**)** 3416, 2923, 2850, 1600, 1501, 1241, 1171, 830, 519.

##### *N*-(1-(4-methoxyphenyl)-2-methylpropyl)aniline (6d)

**Figure:**
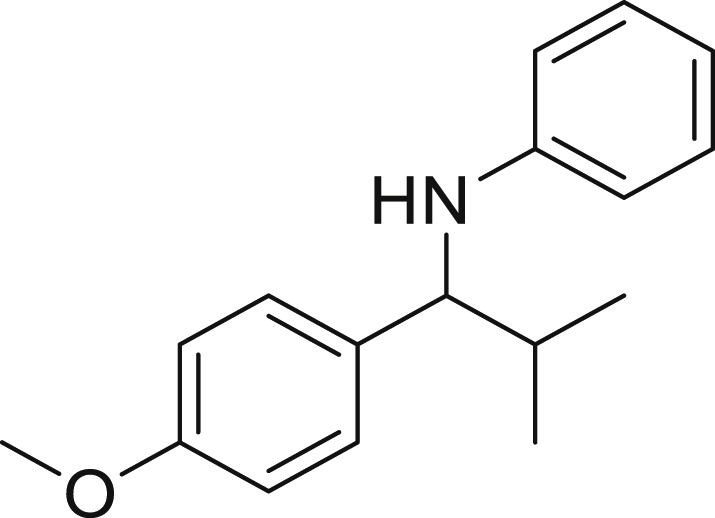

**6d** (61 mg, 60%), was prepared according to the general procedure. The desired amine **6d** was isolated through flash column chromatography (eluent: heptane/EtOAc, 98:2) as a green solid, m.p. 27°C.

^1^H NMR (400 MHz, Chloroform-*d*) δ 7.24–7.19 (m, 2H), 7.13–7.03 (m, 2H), 6.89–6.82 (m, 2H), 6.66–6.57 (m, 1H), 6.55–6.47 (m, 2H), 4.13–4.05 (m, 2H), 3.79 (s, 3H), 2.07–1.96 (m, 1H), 1.33–1.25 (m, 1H), 0.99 (d, *J* = 6.8 Hz, 3H), 0.92 (d, *J* = 6.7 Hz, 3H).

^13^C NMR (101 MHz, Chloroform-d) δ 158.52, 147.92, 134.65, 129.16, 128.27, 117.05, 113.71, 113.36, 63.32, 55.31, 35.07, 19.72, 18.87.

**HRMS** (ESI^+^): [M-H] calculated for C_17_H_21_NO: 254.1550, found: 254.1536.

**IR (neat,** ν**/**cm^−1^**)** 3404, 2998, 2955, 2870, 1585, 1464, 1170, 1026, 748, 592.

##### *N*-(benzo[d]thiazol-2-yl(tetrahydro-2H-pyran-4-yl)methyl)aniline (6e)

**Figure:**
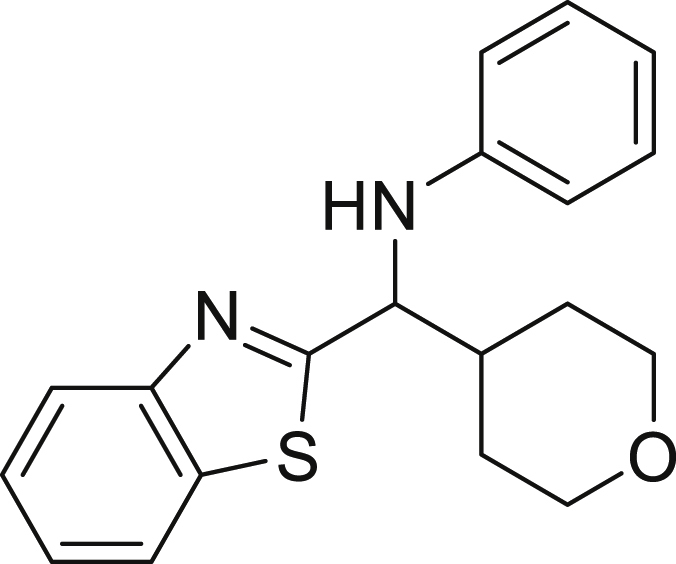

**6e** (36 mg, 65%), was prepared according to the general procedure, but using 0.17 mmol of 2-benzothiazolecarbozaldehyde limiting reagent. The desired amine **6e** was isolated through flash column chromatography as a yellow oil (eluent: heptane/EtOAc, 98:2).

^1^H NMR (400 MHz, Chloroform-d) δ 8.04–7.98 (m, 1H), 7.83–7.78 (m, 1H), 7.52–7.44 (m, 1H), 7.40–7.32 (m, 1H), 7.18–7.10 (m, 2H), 6.78–6.70 (m, 1H), 6.70–6.64 (m, 2H), 4.69 (t, J = 5.6 Hz, 1H), 4.34 (s, 1H), 4.10–3.92 (m, 2H), 3.46–3.33 (m, 2H), 2.39–2.24 (m, 1H), 1.85–1.77 (m, 1H), 1.74–1.63 (m, 2H), 1.60–1.51 (m, 1H).

^13^C NMR (101 MHz, Chloroform-*d*) δ 176.17, 153.68, 146.82, 134.97, 129.48, 126.11, 125.07, 122.99, 121.94, 118.85, 113.65, 67.87, 67.80, 61.76, 41.65, 30.02, 29.10.

**HRMS** (ESI^+^): [M + H] calculated for C_19_H_20_N_2_OS: 325.1370, found: 325.1373.

**IR (neat,** ν**/**cm^−1^**)** 3354, 3053, 2918, 2845, 1600, 1499, 1312, 1088, 749, 727, 691.

##### *N*-(1-(benzo[d]thiazol-2-yl)pentyl)aniline (6f)

**Figure:**
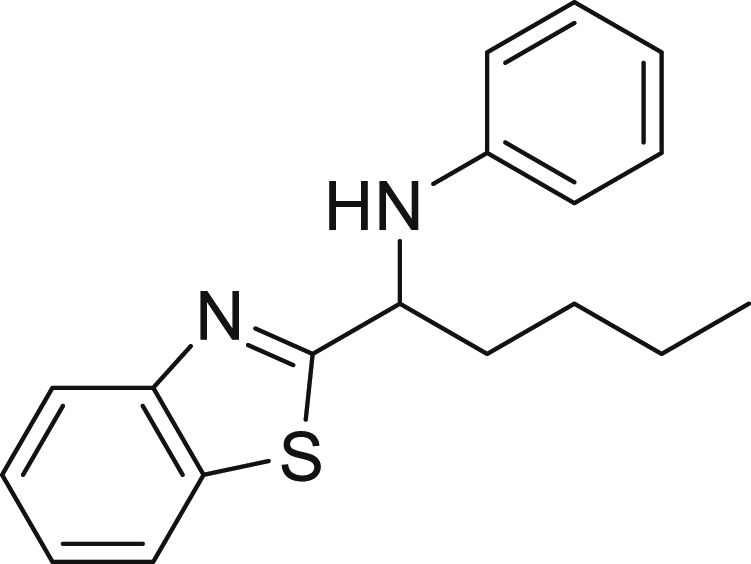

**6f** (77 mg, 65%), was prepared according to the general procedure. The desired amine **6f** was isolated through flash column chromatography (eluent: heptane/EtOAc, 98:2) as a yellow solid, m.p. 79°C.

^1^H NMR (300 MHz, Chloroform-d) δ 8.01 (d, J = 8.1 Hz, 1H), 7.81 (d, J = 7.9 Hz, 1H), 7.47 (t, J = 7.7 Hz, 1H), 7.34 (t, J = 7.6 Hz, 1H), 7.15 (t, J = 7.7 Hz, 2H), 6.80–6.58 (m, 3H), 4.85–4.75 (m, 1H), 4.29 (s, 1H), 2.16–1.89 (m, 2H), 1.58–1.36 (m, 4H), 0.99–0.88 (m, 3H).

^13^C NMR (75 MHz, Chloroform-d) δ 178.59, 153.81, 146.82, 135.09, 129.42, 125.96, 124.87, 122.89, 121.99, 118.65, 113.55, 57.64, 37.31, 28.25, 22.60, 14.04.

**HRMS** (ESI^+^): [M-H] calculated for C_18_H_20_N_2_S: 295.1274, found: 295.1267.

**IR (neat,** ν**/**cm^−1^**)** 3349, 2925, 2856, 1673, 1600, 1497, 1311, 749, 729.

##### *N*-(1-(benzo[d]thiazol-2-yl)-3-methylbutyl)aniline (6g)

**Figure:**
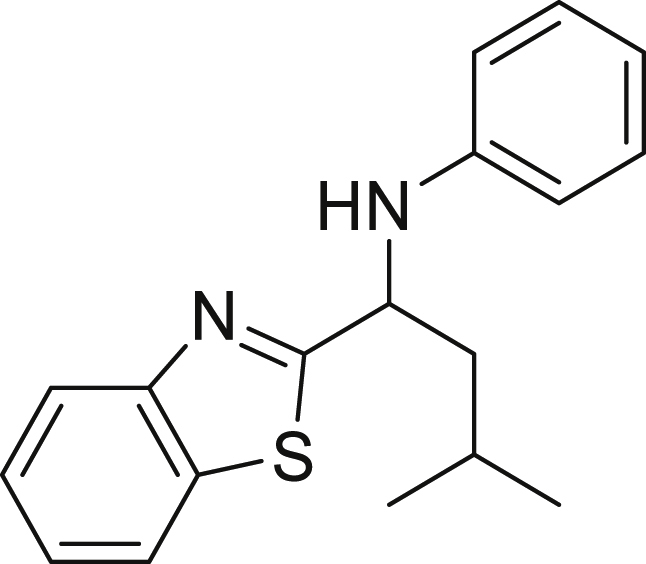

**6g** (40 mg, 40%), was prepared according to the general procedure. The desired amine **6g** was isolated through flash column chromatography (eluent: heptane/EtOAc, 98:2) as a yellow solid, m.p. 91°C.

^1^H NMR (300 MHz, Chloroform-d) δ 8.01 (d, *J* = 8.2 Hz, 1H), 7.80 (d, *J* = 7.9 Hz, 1H), 7.46 (t, *J* = 7.7 Hz, 1H), 7.33 (t, *J* = 7.6 Hz, 1H), 7.14 (t, *J* = 8.0 Hz, 2H), 6.80–6.61 (m, 3H), 4.88 (dd, *J* = 8.6, 5.1 Hz, 1H), 4.23 (s, 1H), 2.04–1.76 (m, 3H), 1.05 (d, *J* = 5.7 Hz, 3H), 1.00 (d, *J* = 6.0 Hz, 3H).

^13^C NMR (75 MHz, Chloroform-d) δ 179.10, 153.84, 146.76, 135.07, 129.44, 125.97, 124.87, 122.88, 121.99, 118.66, 113.49, 55.86, 46.70, 25.18, 23.19, 22.11.

**HRMS** (ESI^+^): [M-H] calculated for C_18_H_20_N_2_S: 295.1274, found: 295.1259.

**IR (neat,** ν**/**cm^−1^**)** 3310, 3027, 2958, 2923, 2867, 1495, 1313, 806, 760, 705, 512.

##### *N*-(1-(benzo[d]thiazol-2-yl)-3-phenylpropyl)aniline (6h)

**Figure:**
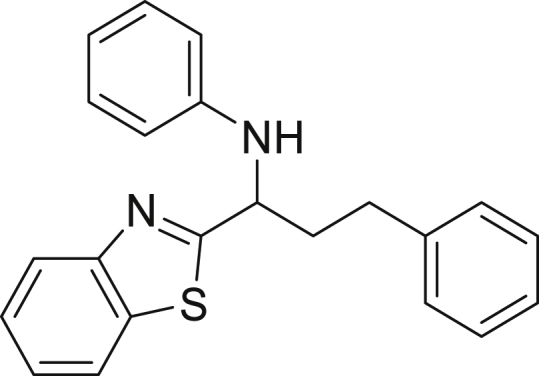

**6h** (78 mg, 57%), was prepared according to the general procedure. The desired amine **6h** was isolated through flash column chromatography (eluent: hexane/EtOAc, 96:4) as a yellow oil.

^1^H NMR (400 MHz, Chloroform-d) δ 8.07–8.01 (m, 1H), 7.87–7.79 (m, 1H), 7.54–7.45 (m, 1H), 7.39–7.28 (m, 3H), 7.25–7.21 (m, 3H), 7.20–7.12 (m, 2H), 6.80–6.73 (m, 1H), 6.67–6.61 (m, 2H), 4.94–4.81 (m, 1H), 4.37–4.25 (m, 1H), 2.95–2.85 (m, 2H), 2.50 (dddd, J = 14.2, 8.8, 7.0, 5.5 Hz, 1H), 2.32 (dtd, J = 13.9, 8.3, 6.6 Hz, 1H).

^13^C NMR (75 MHz, Chloroform-*d*) δ 177.96, 153.71, 146.56, 140.85, 135.02, 129.39, 128.69, 128.62, 126.34, 126.03, 124.96, 122.90, 121.96, 118.72, 113.62, 57.11, 38.80, 32.43. Traces of hexane at δ 31.64, 22.70, 14.14.

**HRMS** (ESI^+^): [M-H] calculated for C_22_H_20_N_2_S: 343.1274, found: 343.1259.

**IR (neat,** ν**/**cm^−1^**)** 3405, 3025, 2922, 2854, 1601, 1498, 1311, 747, 691, 507.

##### *N*-(1-(benzo[d]thiazol-2-yl)2-phenylethyl)aniline (6i)

**Figure:**
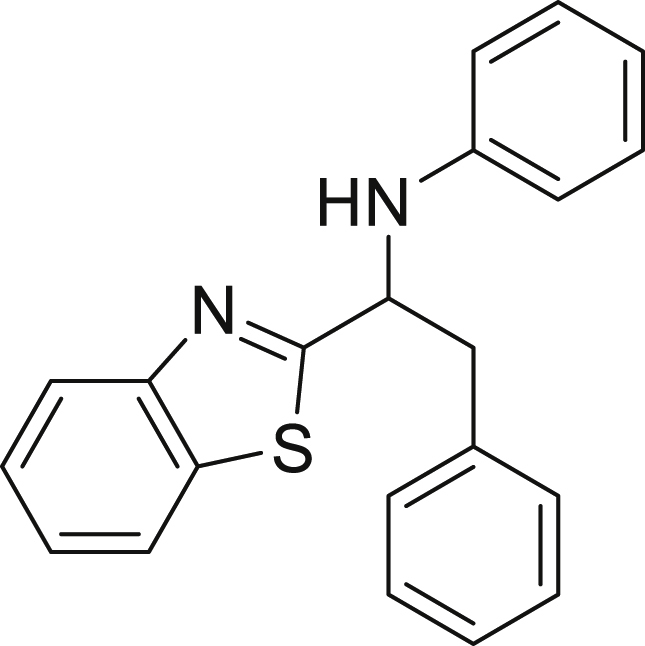

**6i** (22 mg, 17%) was prepared according to the general procedure. The desired amine **6i** was isolated through flash column chromatography as a yellow oil (eluent: heptane/EtOAc, 97:3).

^1^H NMR (300 MHz, Chloroform-d) δ 8.04 (d, J = 8.1 Hz, 1H), 7.83 (d, J = 8.0 Hz, 1H), 7.57–7.45 (m, 1H), 7.40–7.36 (m, 1H), 7.33–7.22 (m, 5H), 7.17–7.06 (m, 2H), 6.80–6.67 (m, 1H), 6.60 (d, J = 8.0 Hz, 2H), 5.16–5.05 (m, 1H), 3.62–3.48 (m, 1H), 3.31–3.18 (m, 1H).

^13^C NMR (101 MHz, Chloroform-d) δ 177.84, 153.87, 146.60, 136.43, 135.19, 129.37, 129.03, 127.40, 126.10, 125.01, 122.96, 122.08, 119.03, 114.02, 58.32, 42.98.

**HRMS** (ESI^+^): [M + H] calculated for C_21_H_18_N_2_S: 331.1263, found: 331.1263.

**IR (neat,** ν**/**cm^−1^**)** 3401, 3026, 2922, 2852, 1678, 1498, 1312, 750, 728, 691, 507.

##### *N*-(benzyl(4-methoxyphenyl)methyl)aniline (6j)

**Figure:**
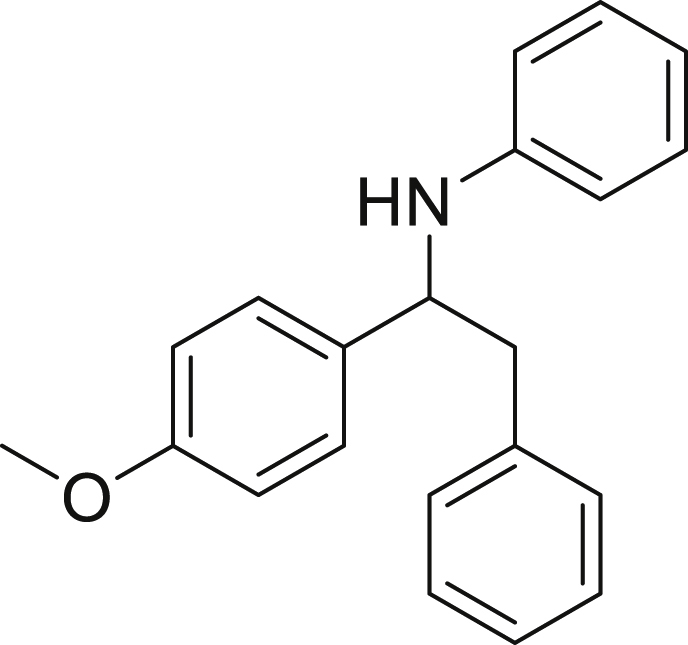

**6j** (59 mg, 49%) was prepared according to the general procedure. The desired amine **6j** was isolated through flash column chromatography as a yellow oil (eluent: heptane/EtOAc, 98:2).

^1^H NMR (300 MHz, Chloroform-*d*) δ 7.37–7.23 (m, 5H), 7.21–7.03 (m, 4H), 6.89 (d, *J* = 8.1 Hz, 2H), 6.68 (t, *J* = 7.3 Hz, 1H), 6.52 (d, *J* = 7.9 Hz, 2H), 4.60 (t, *J* = 6.9 Hz, 1H), 4.14 (s, 1H), 3.83 (s, 3H), 3.22–2.97 (m, 2H).

^13^C NMR (101 MHz, Chloroform-d) δ 158.73, 147.47, 137.93, 135.52, 129.38, 129.14, 128.64, 127.63, 126.78, 117.55, 114.07, 113.78, 58.76, 55.37, 45.36.

**IR (neat,** ν**/**cm^−1^**)** 3408, 3025, 2926, 2834, 1600, 1503, 1242, 748, 538.

##### *N*-(benzo[d]thiazol-2-yl(but-3-en-1-yl)methyl)aniline (6k)

**Figure:**
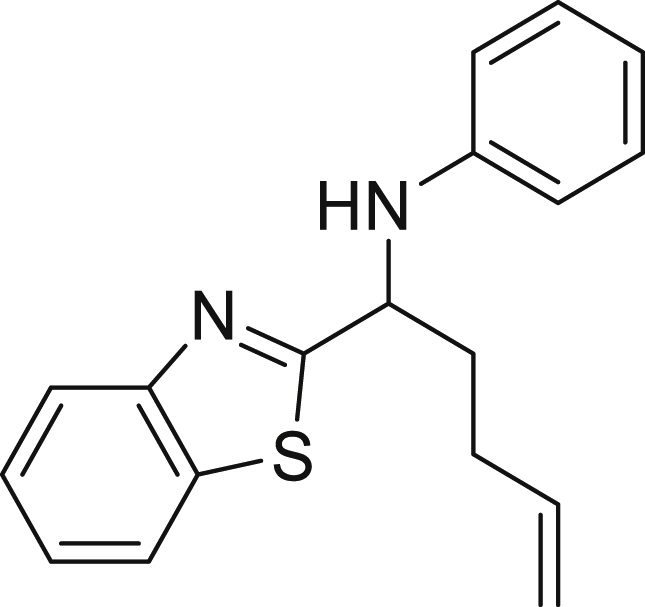

**6k** (37 mg, 31%) was prepared according to the general procedure. The desired amine **6k** was isolated through flash column chromatography as a yellow oil (eluent: heptane/EtOAc, 97:3).

^1^H NMR (300 MHz, Chloroform-*d*) δ 8.02 (d, J = 8.2 Hz, 1H), 7.83 (d, J = 7.9 Hz, 1H), 7.49 (t, J = 7.7 Hz, 1H), 7.36 (t, J = 7.5 Hz, 1H), 7.22–7.08 (m, 2H), 6.83–6.71 (m, 1H), 6.68 (d, J = 7.9 Hz, 2H), 6.00–5.74 (m, 1H), 5.19–4.99 (m, 2H), 4.95–4.79 (m, 1H), 4.33 (s, 1H), 2.45–2.22 (m, 3H), 2.18–2.01 (m, 1H).

^13^C NMR (101 MHz, Chloroform-d) δ 178.12, 153.82, 146.67, 137.16, 135.08, 129.45, 126.04, 124.95, 122.93, 122.01, 118.75, 116.17, 113.60, 57.11, 36.49, 30.28.

**HRMS** (ESI^+^): [M + H] calculated for C_18_H_18_N_2_S: 295,1263 found: 295.1266.

**IR (neat,** ν**/**cm^−1^**)** 3403, 3055, 2923, 2853, 1640, 1601, 1312, 749, 691, 509.

#### Mechanistic investigation

**Stern-Volmer quenching experiment procedure:** the experiment was performed on a fluorescence spectrophotometer (Fluorolog, HORIBA Instruments, Photonic division). A 3 μM solution in acetonitrile of [Ir{dF(CF_3_)ppy}_2_(dtbbpy)]PF_6_ was portioned and mixed with appropriate amounts of quencher. The newly prepared solutions were degassed and kept under nitrogen atmosphere. Upon measuring, the solutions were transferred to a 1.0 cm quartz cuvette (Hellma 111-Qs) capped with a homemade silicone septum and purged with nitrogen. The solutions were irradiated at 400 nm and emission was measured at 500 nm. The relative intensity I0/I (Figure S1) was calculated as a function of quencher concentration, where I0 is the luminescence intensity in the absence of quencher, whilst I is the intensity in the presence of the quencher.

**Cyclic voltammetry measurements**: the experiments were conducted using a cyclic potentiometer (Metrohm PGSTAT204 potentiostat/galvanostat) with a glassy carbon working electrode, a Pt counter electrode and an Ag/AgCl reference electrode [referenced to SCE using ferrocene (Fc) as an internal standard (0.42 V *vs*. SCE)]. In the standard procedure, 0.02 mmol of substrate were dissolved in 10 mL of a 0.1 M [N(Bu)_4_]PF_6_ electrolyte solution in degassed MeCN. The reactor was sealed with a rubber septum and purged with nitrogen. Each measurement was conducted at 100 mV/s at room temperature under nitrogen atmosphere without stirring.

The new local maximum at 0.62 V *vs.* SCE was calculated using equation: f(Ep/2)=Cmax/2 (Figure S2).

**Light-Dark experiment:** the experiment excludes a radical-chain mechanism, since in the absence of light no product formation was detected (Figure S3).

**Radical inhibition experiment**: adding TEMPO as radical quencher to the reaction mixture, the adduct between cyclopentyl and TEMPO itself has been detected by GC-MS analysis. This result support a radical based mechanism (Scheme S1).

## Data Availability

• All data reported in this paper will be shared by the lead contact upon request. • This paper does not report original code. • Any additional information required to reanalyze the data reported in this paper is available from the lead contact upon request.

## Supplemental information

Document S1. Figures S1–S3 and Scheme S1
