# Supplementary material for: Photoredox-catalyzed multicomponent Petasis reaction in batch and continuous flow with alkyl boronic acids
Source: iScience. 2021 Sep 15;24(10):103134. doi: 10.1016/j.isci.2021.103134 (PMC8487034; doi:10.1016/j.isci.2021.103134)

ISCI, Volume *24*

Supplemental information

Photoredox-catalyzed multicomponent Petasis reaction in batch and continuous flow with alkyl boronic acids

Monica Oliva, Prabhat Ranjan, Serena Pillitteri, Guglielmo Attilio Coppola, Monica Messina, Erik V. Van der Eycken, and Upendra Kumar Sharma

Figure S1. Stern-Volmer quenching experiment, related to STAR Methods

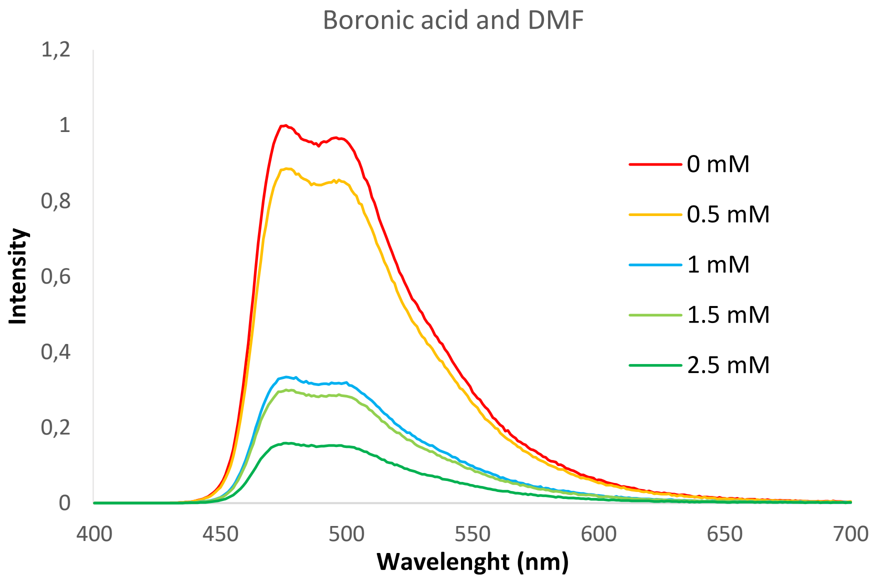

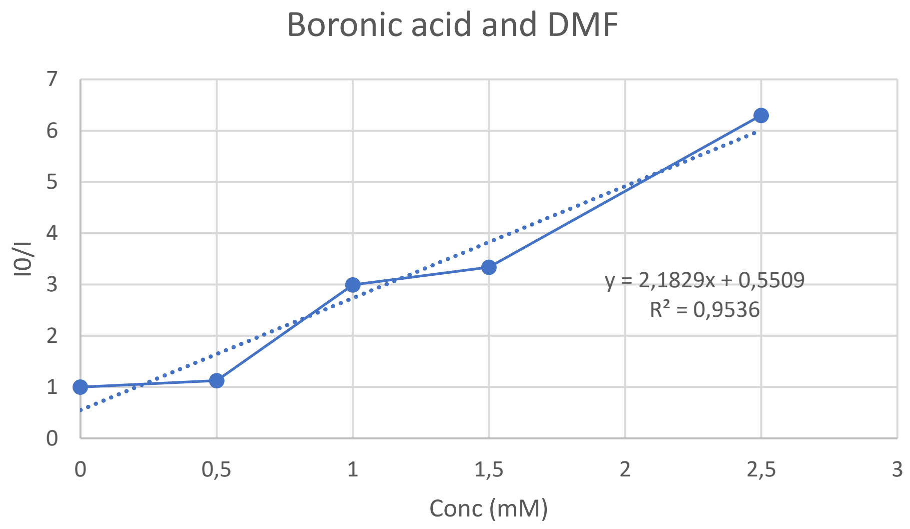

Figure S2. Cyclic voltammetry measurements of phenyl ethyl boronic acid in the presence of DMF, related to STAR Methods

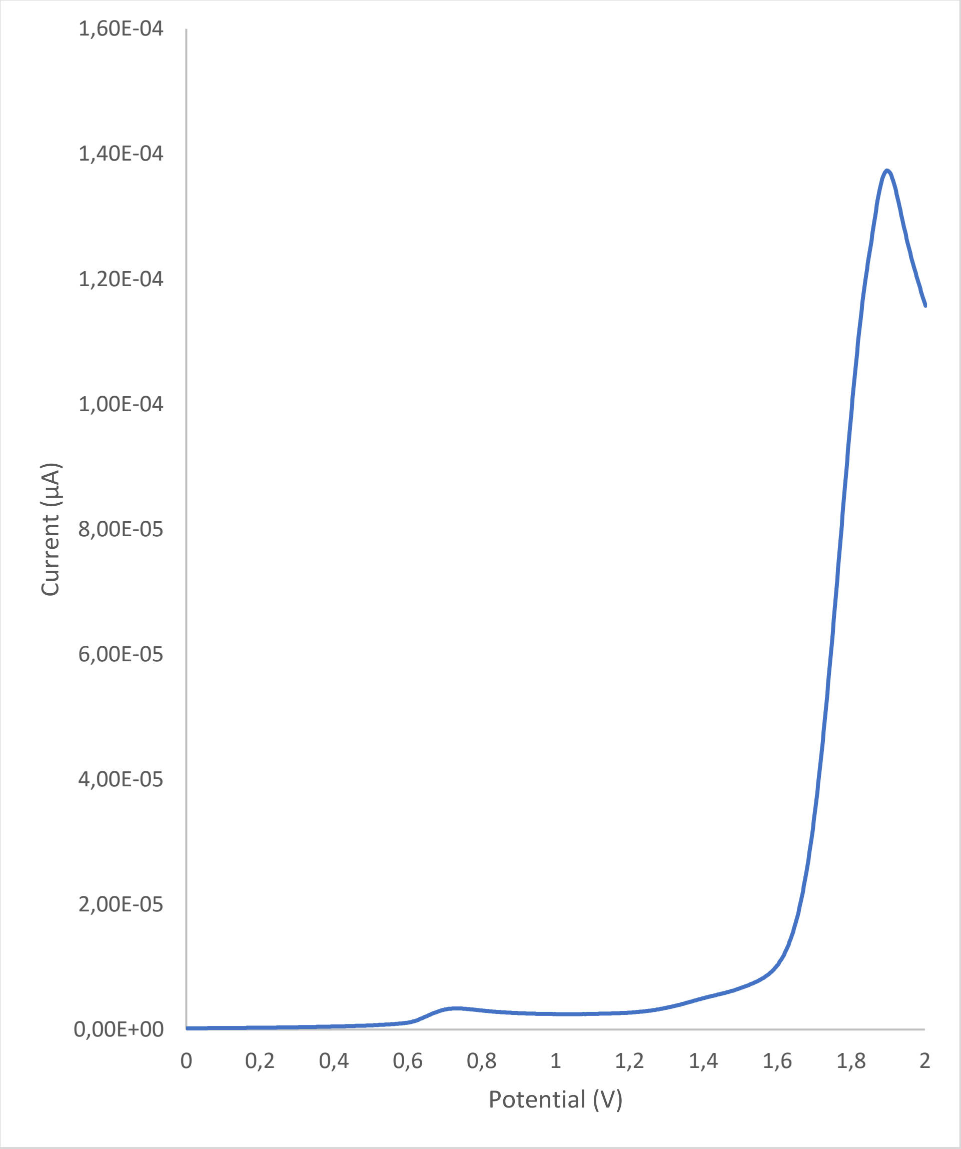

Figure S3. Light-dark experiment, related to STAR Methods

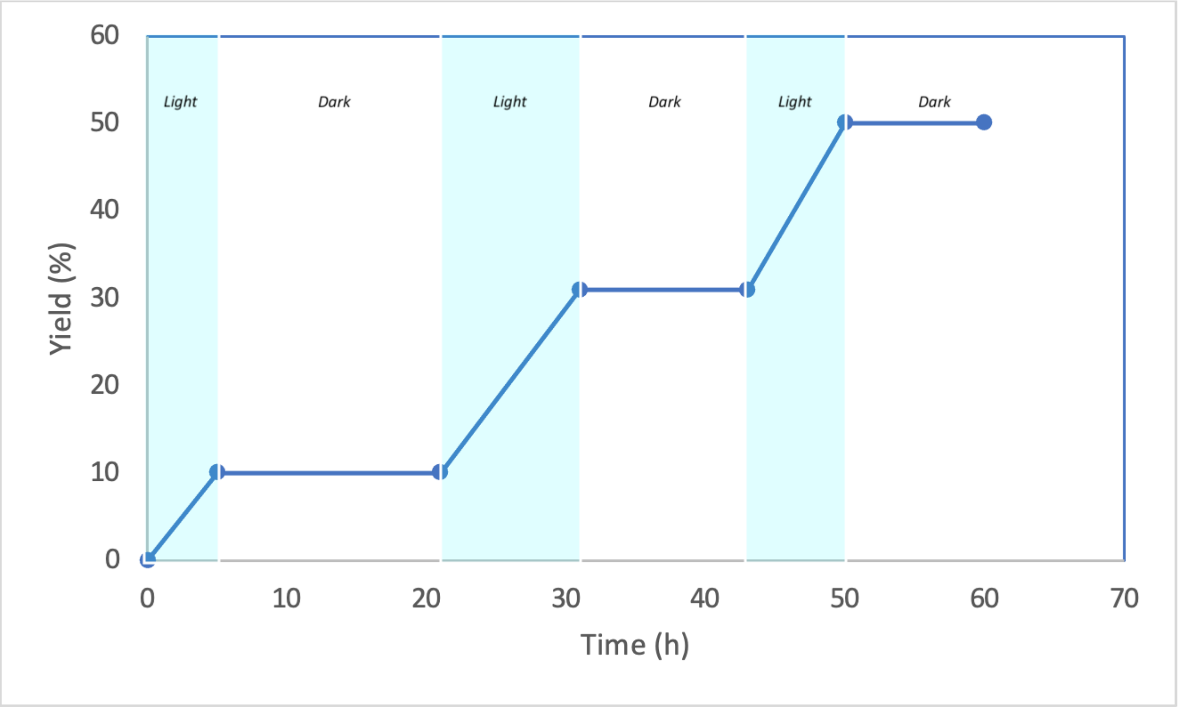

Scheme S1. Radical inhibition experiment, related to STAR Methods

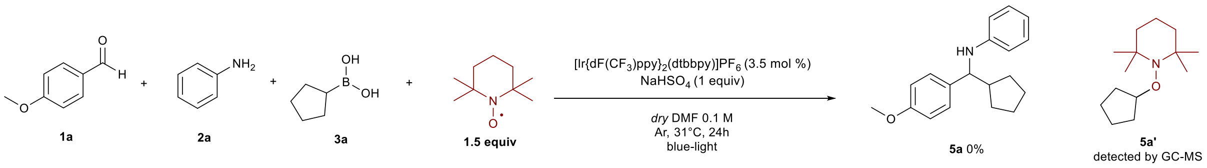

Spectra of synthesized compounds

^{1}H NMR spectrum of compound **4a** ($CDCl_3$, 300 MHz).

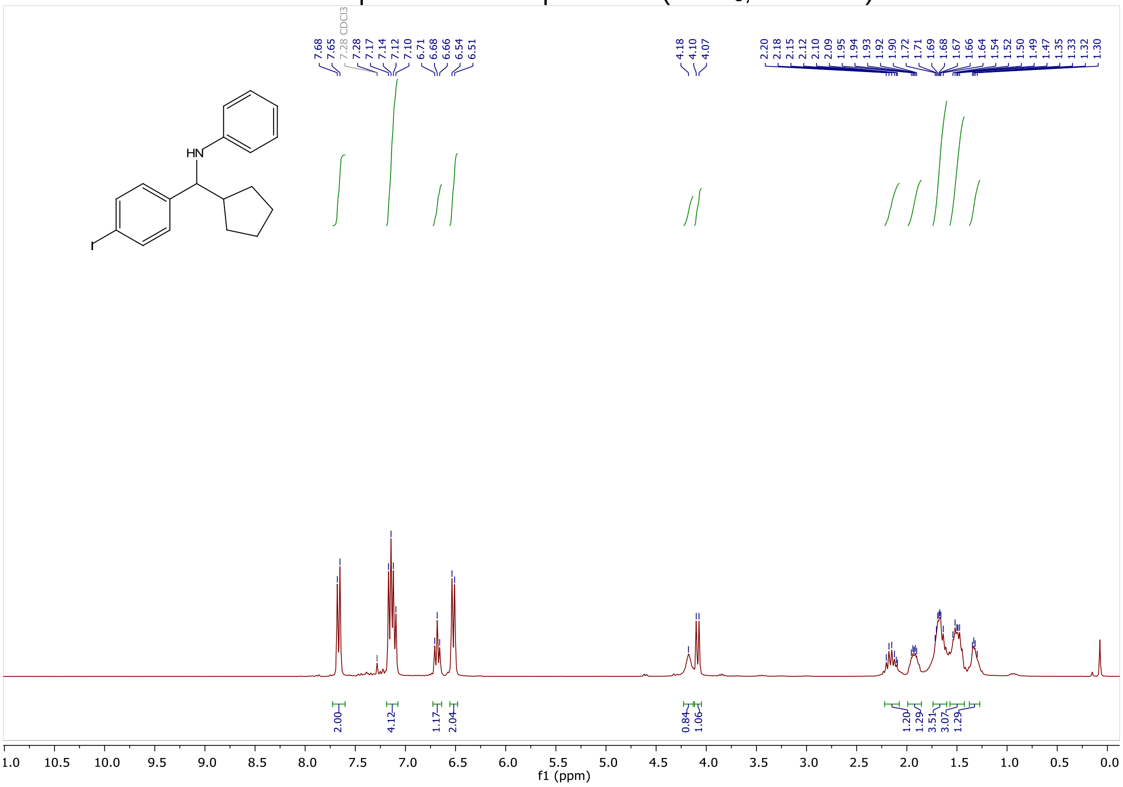

^{13}C NMR spectrum of compound **4a** ($CDCl_3$, 75 MHz).

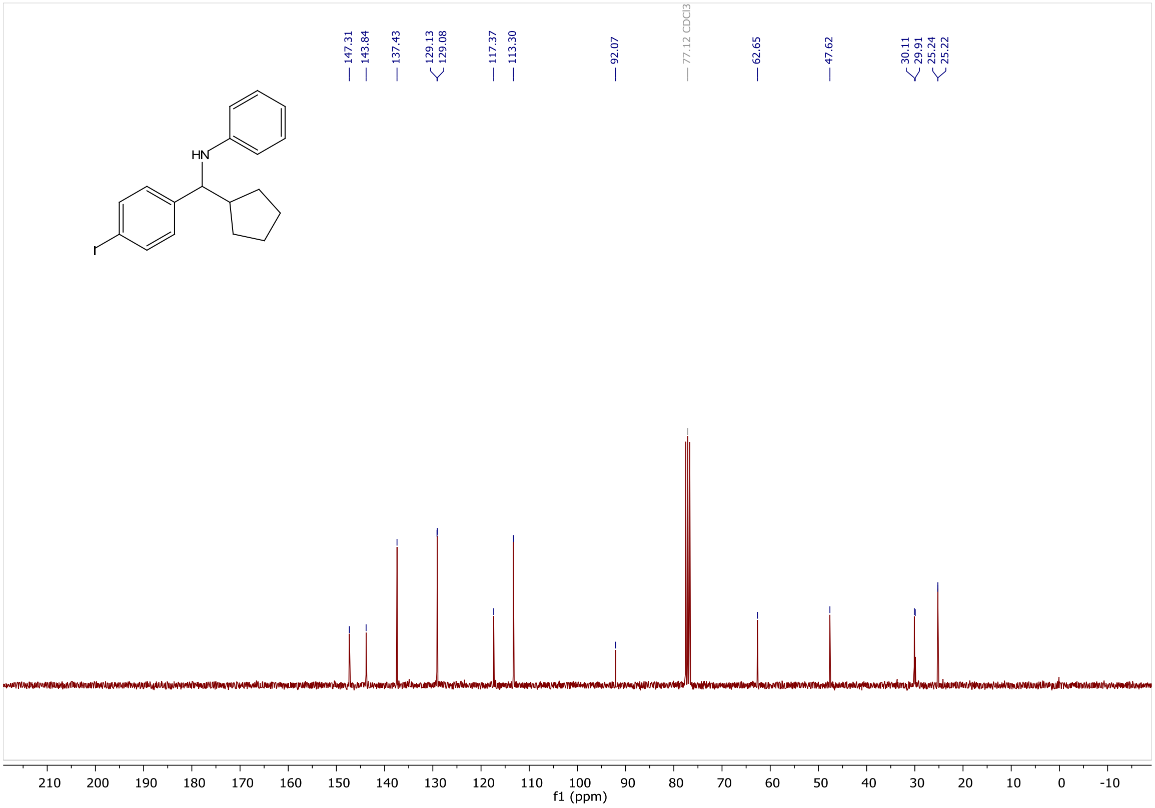

^{1}H NMR spectrum of compound **4b** ($CDCl_3$, 300 MHz).

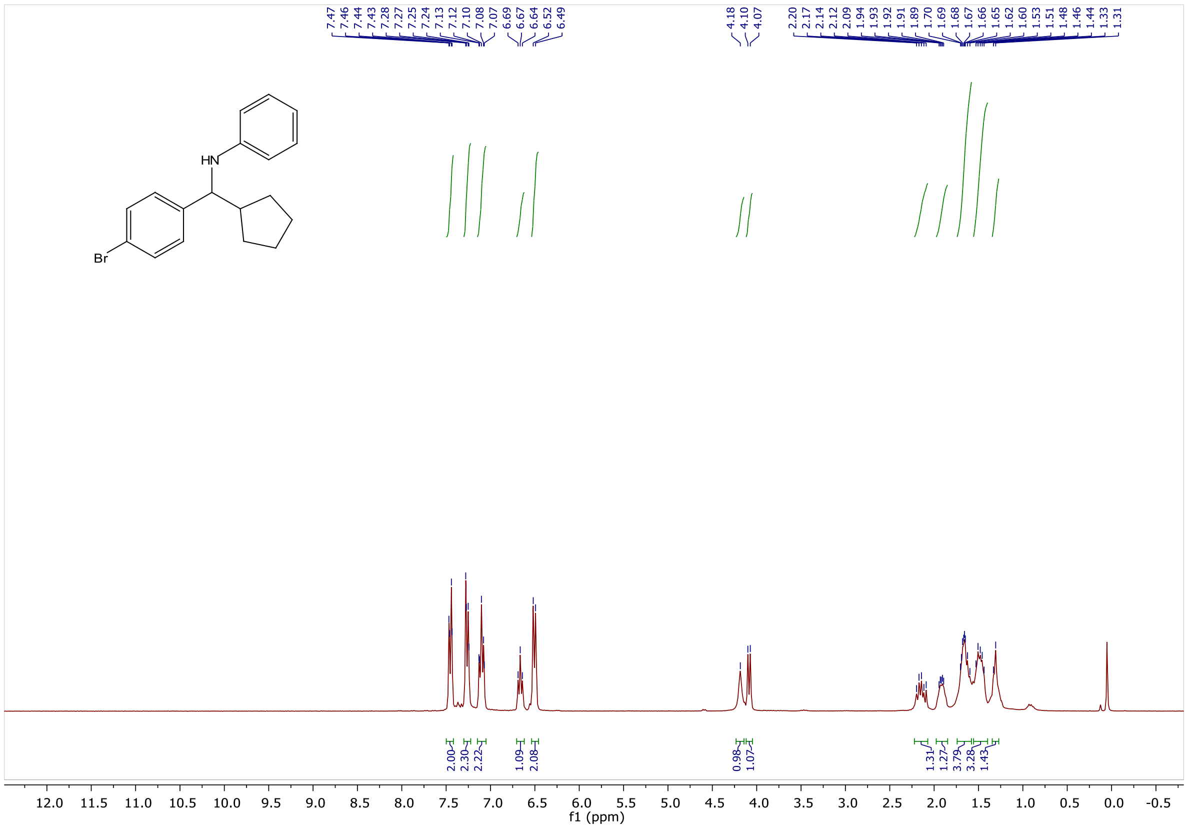

^{13}C NMR spectrum of compound **4b** ($CDCl_3$, 101 MHz).

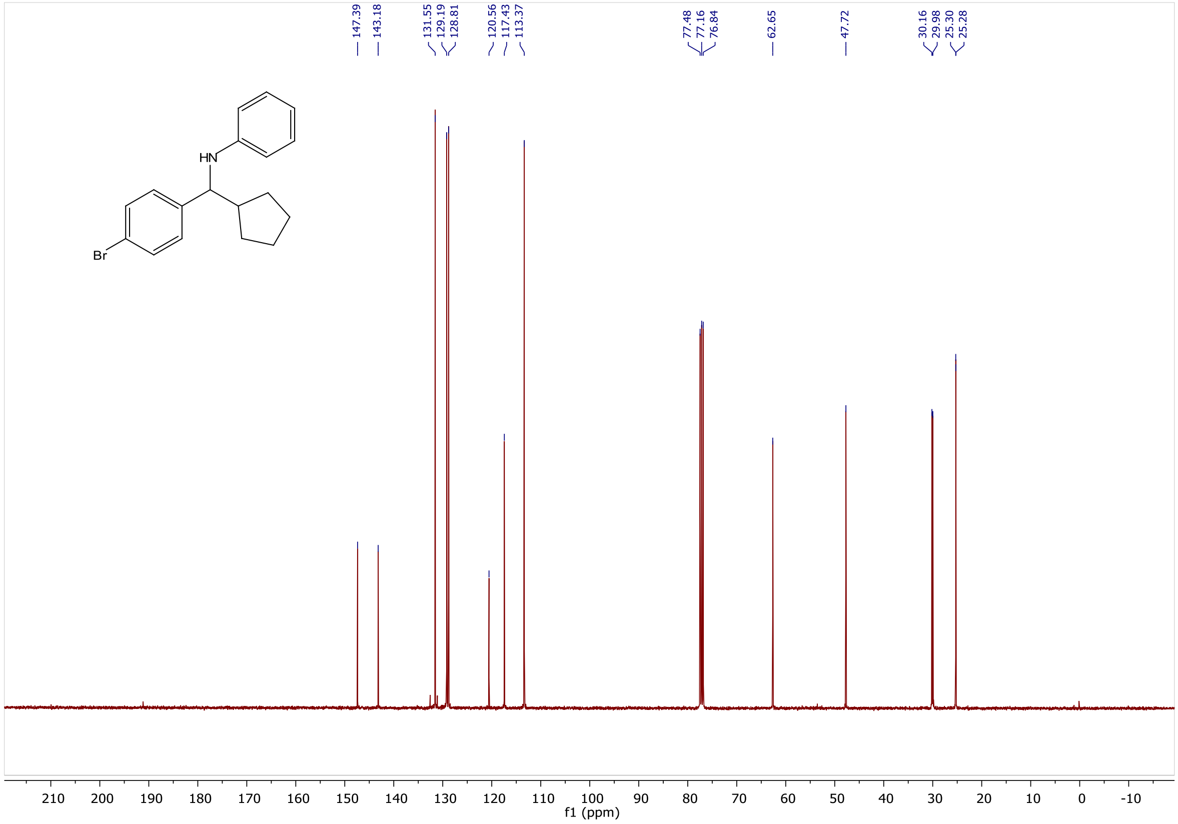

^{1}H NMR spectrum of compound **4c** ($CDCl_3$, 300 MHz).

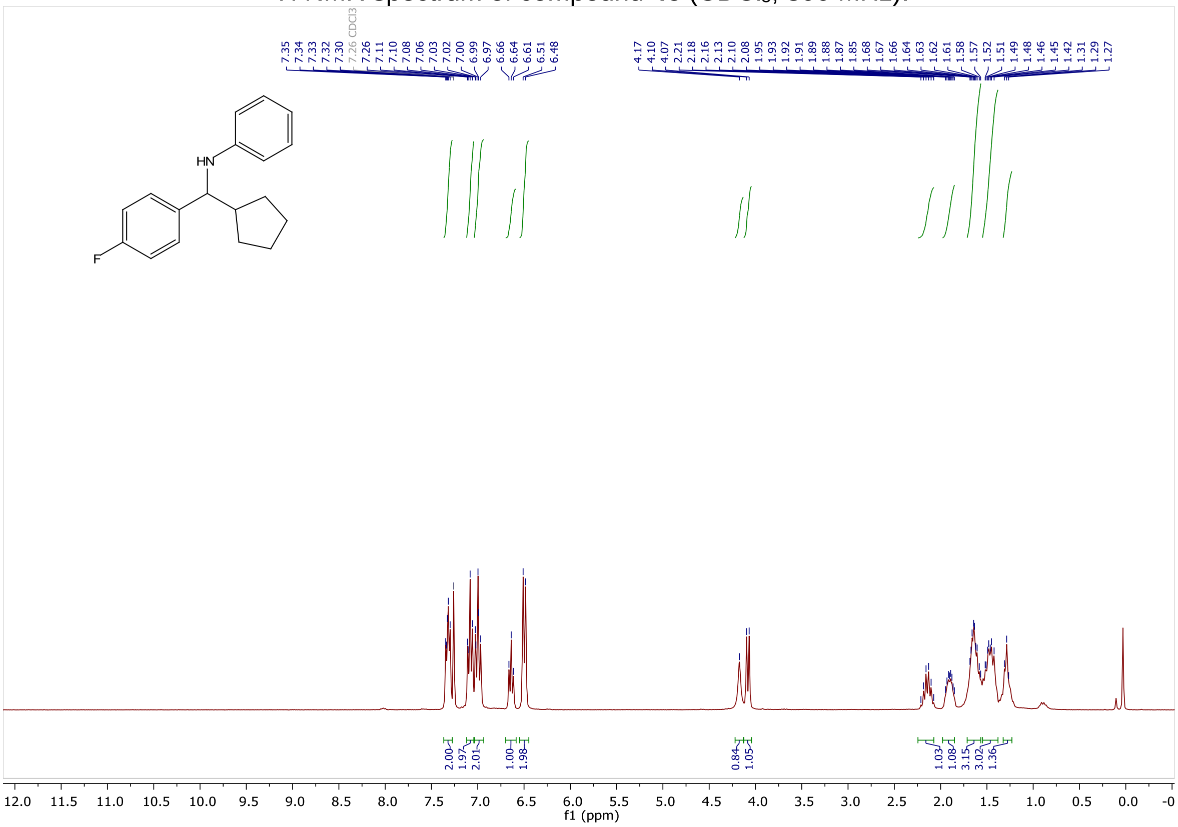

^{13}C NMR spectrum of compound **4c** ($CDCl_3$, 101 MHz).

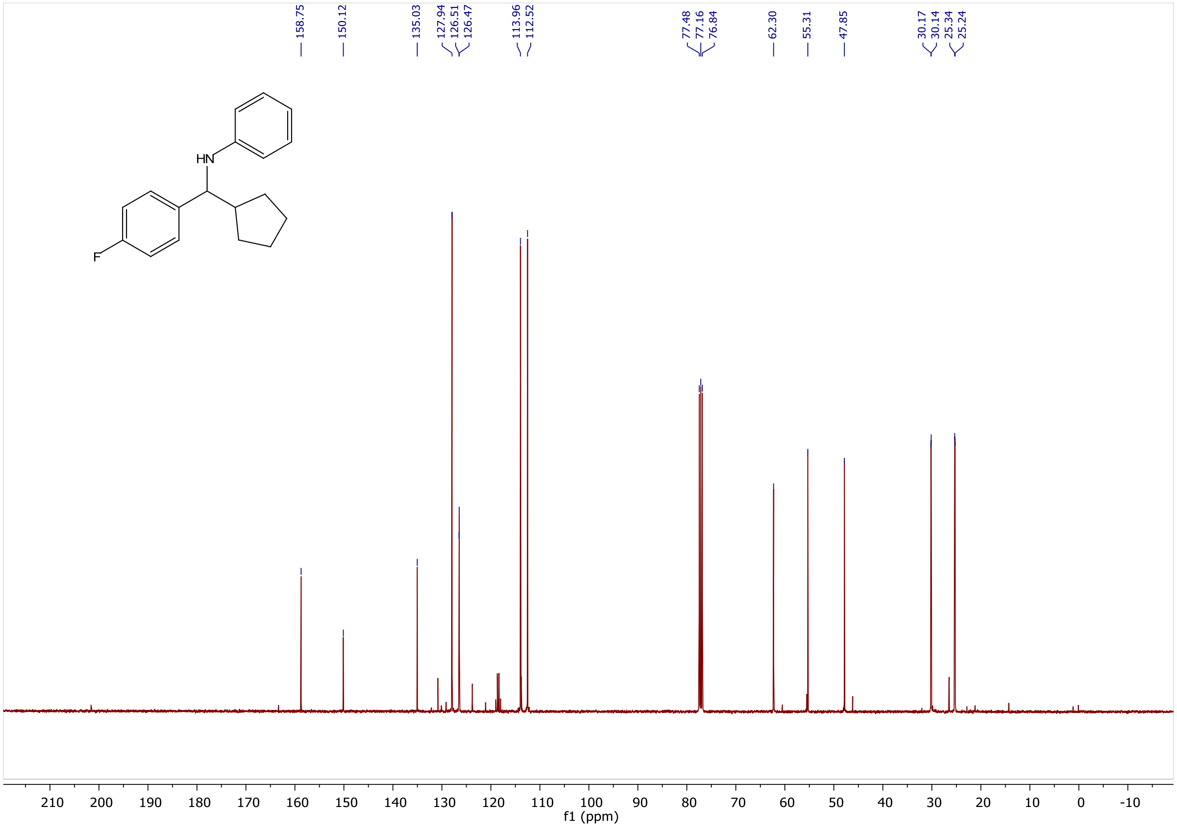

^{1}H NMR spectrum of compound **4d** ($CDCl_3$, 300 MHz).

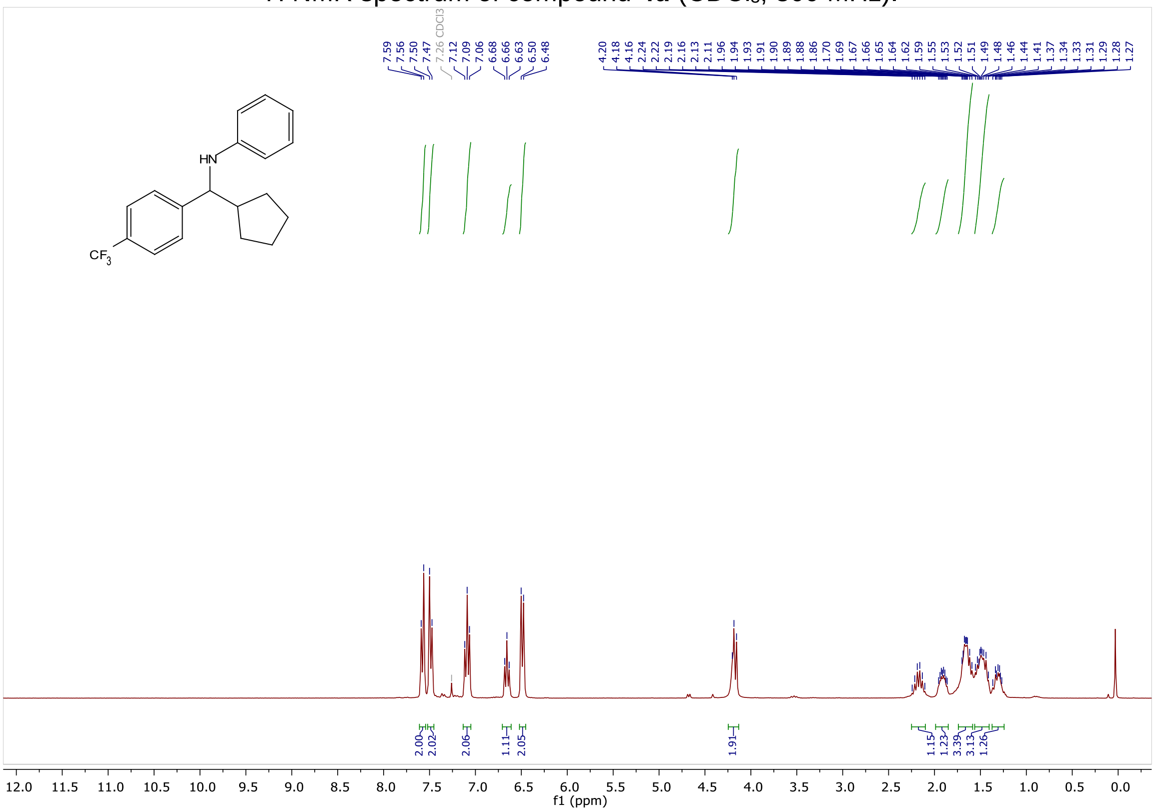

^{13}C NMR spectrum of compound **4d** ($CDCl_3$, 75 MHz).

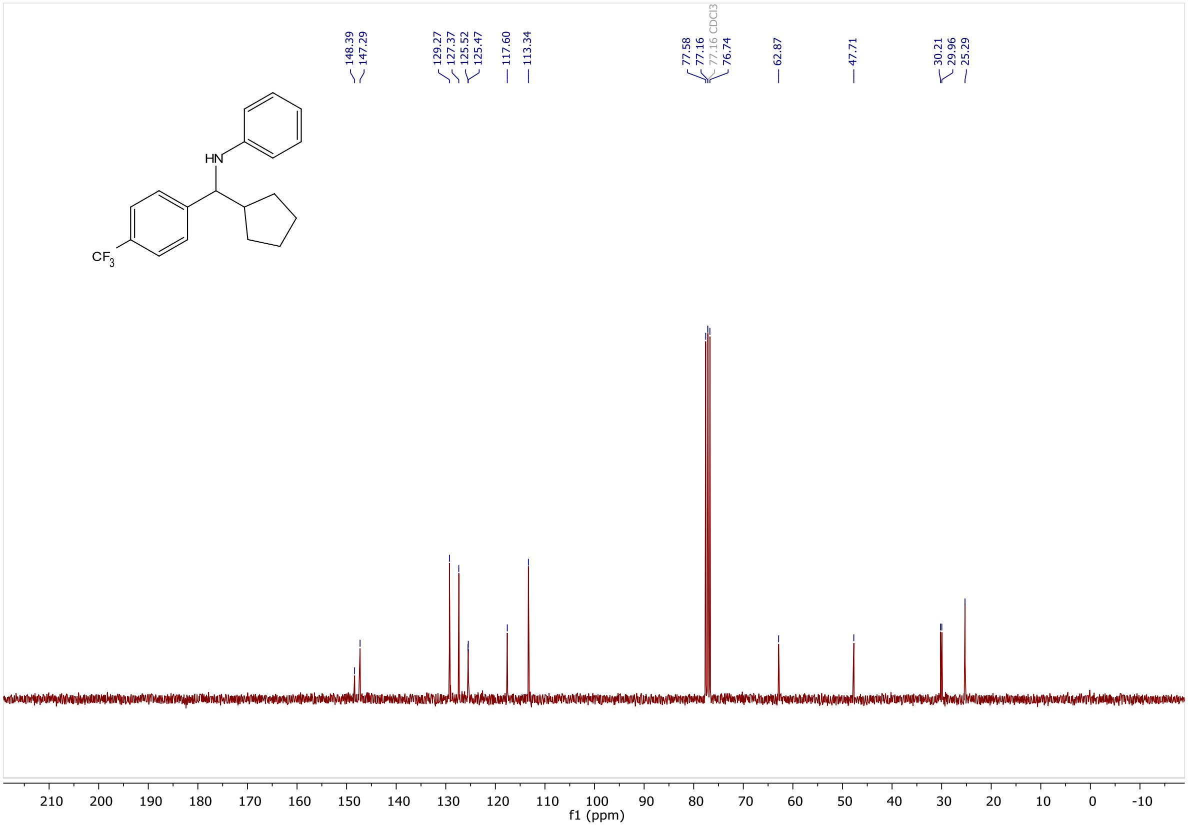

^{1}H NMR spectrum of compound **4e** ($CDCl_3$, 300 MHz).

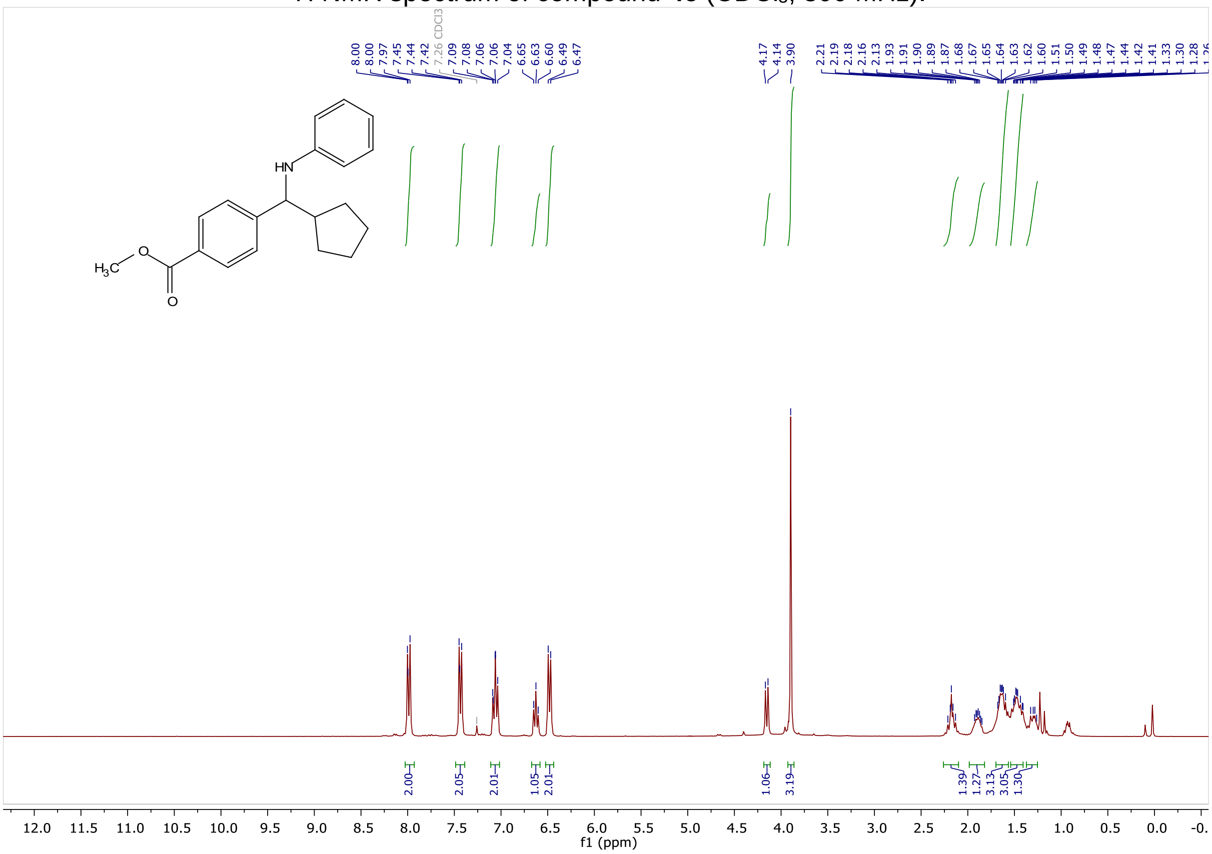

^{13}C NMR spectrum of compound **4e** ($CDCl_3$, 75 MHz).

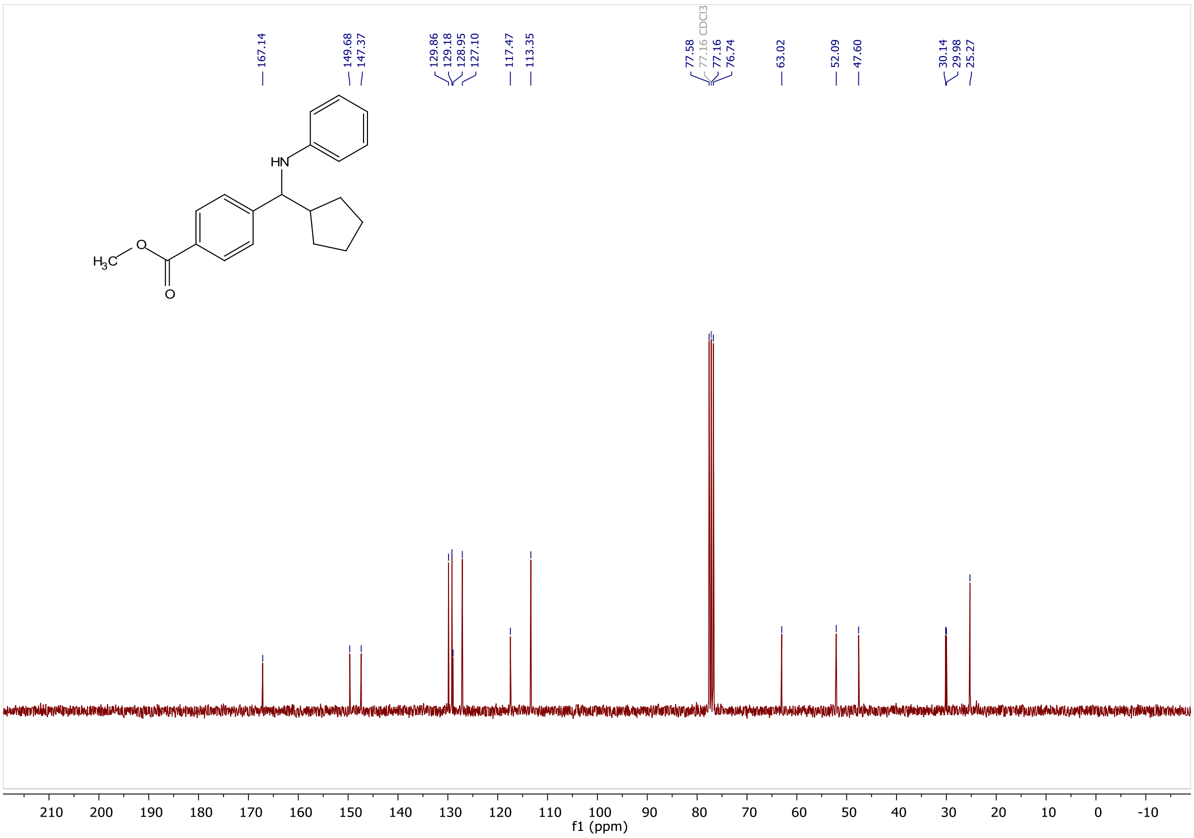

^{1}H NMR spectrum of compound **4f** ($CDCl_3$, 300 MHz).

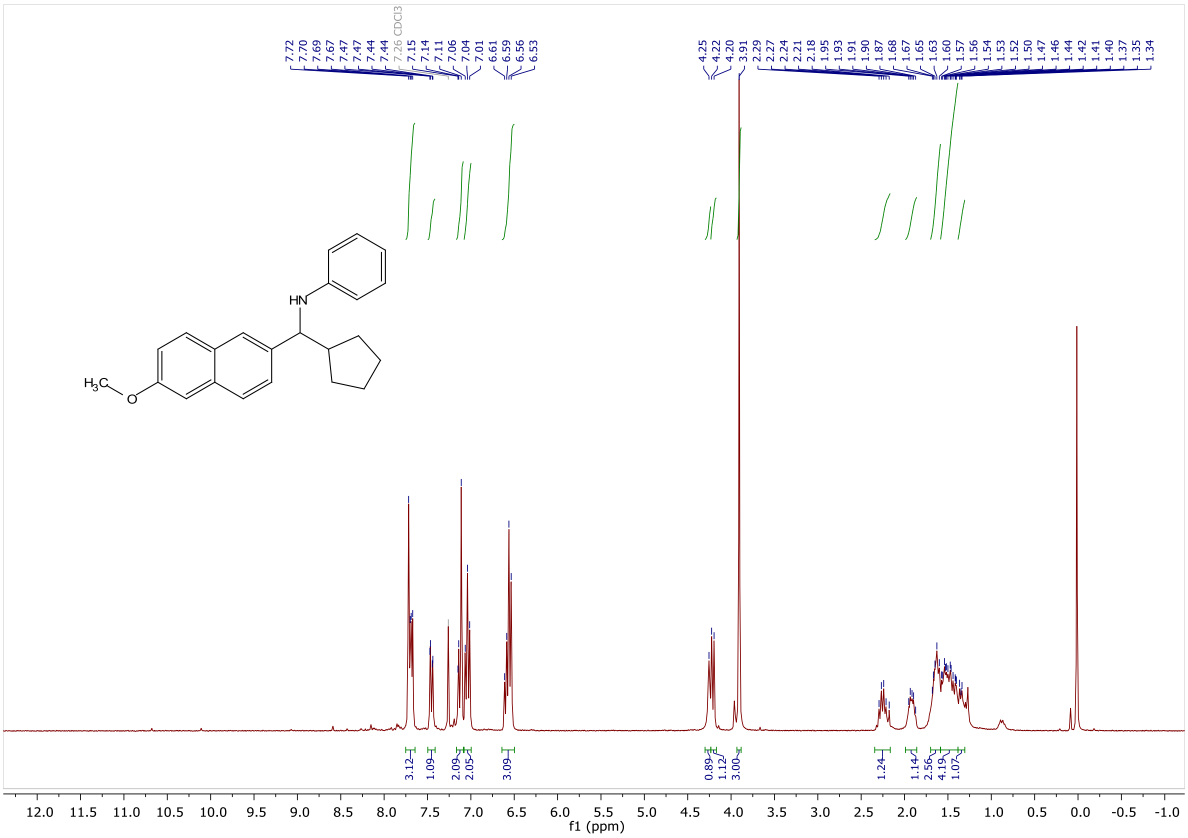

^{13}C NMR spectrum of compound **4f** ($CDCl_3$, 101 MHz).

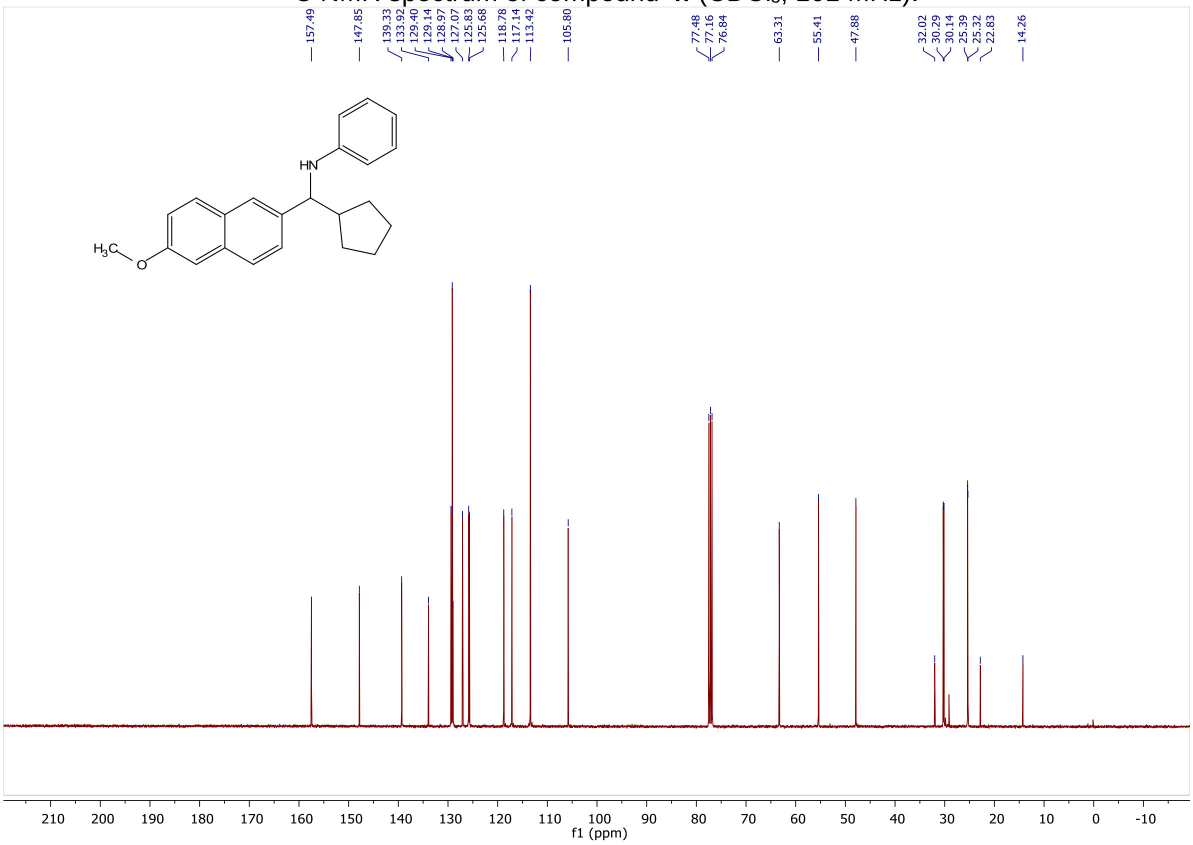

^{1}H NMR spectrum of compound **4g** ($CDCl_3$, 300 MHz).

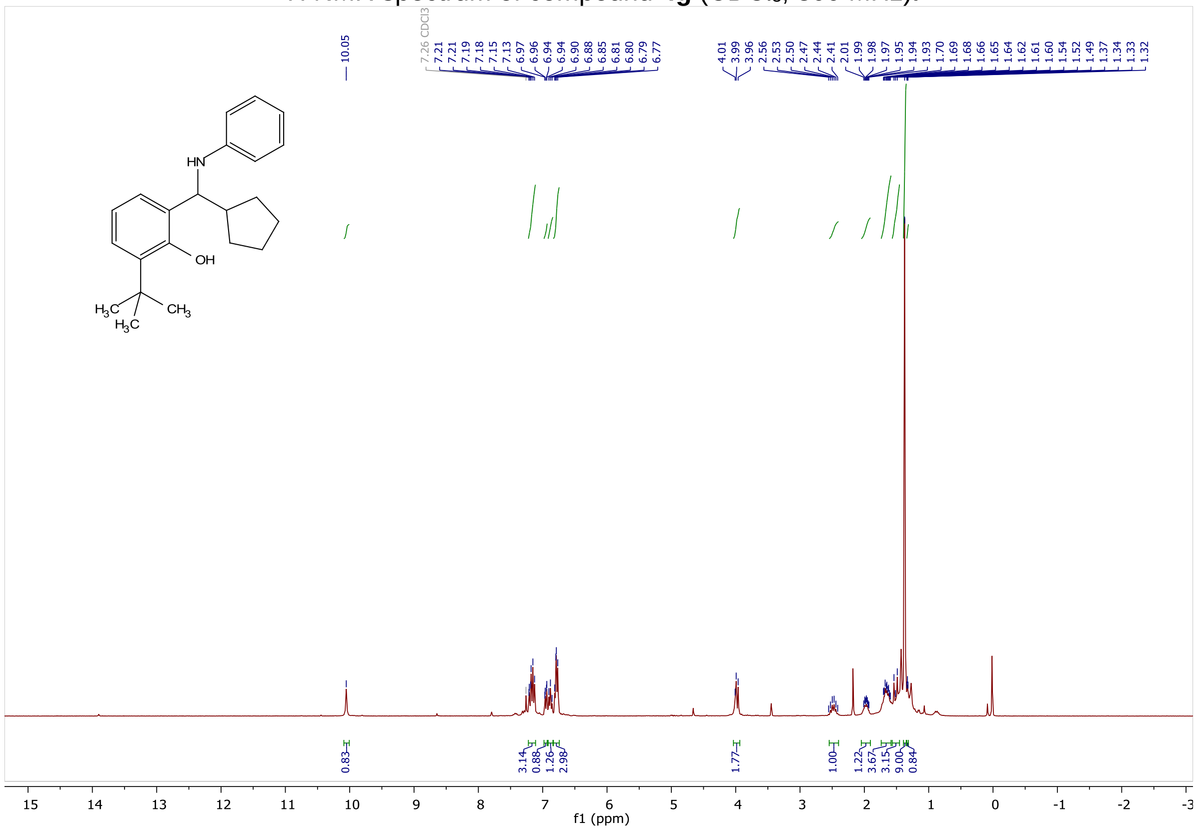

^{13}C NMR spectrum of compound **4g** ($CDCl_3$, 101 MHz).

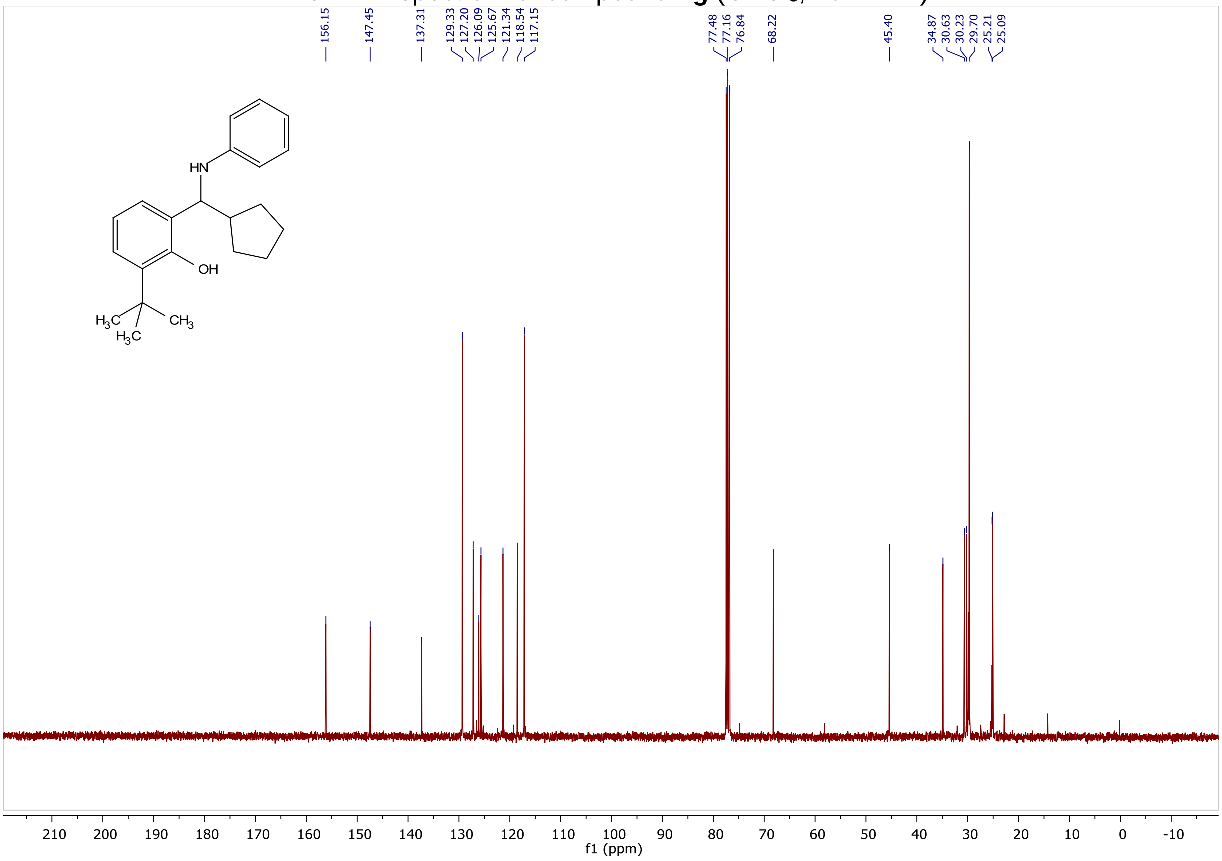

^{1}H NMR spectrum of compound **4h** ($CDCl_3$, 300 MHz).

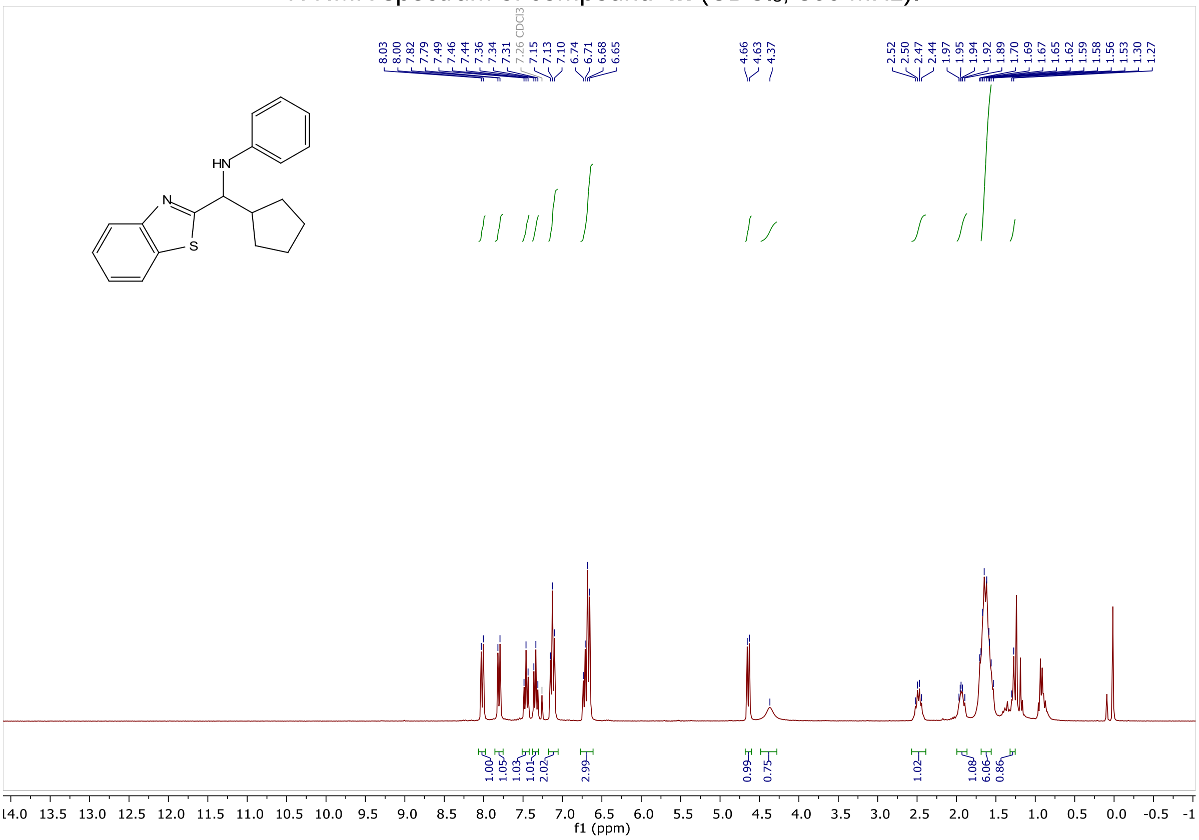

^{13}C NMR spectrum of compound **4h** ($CDCl_3$, 75 MHz).

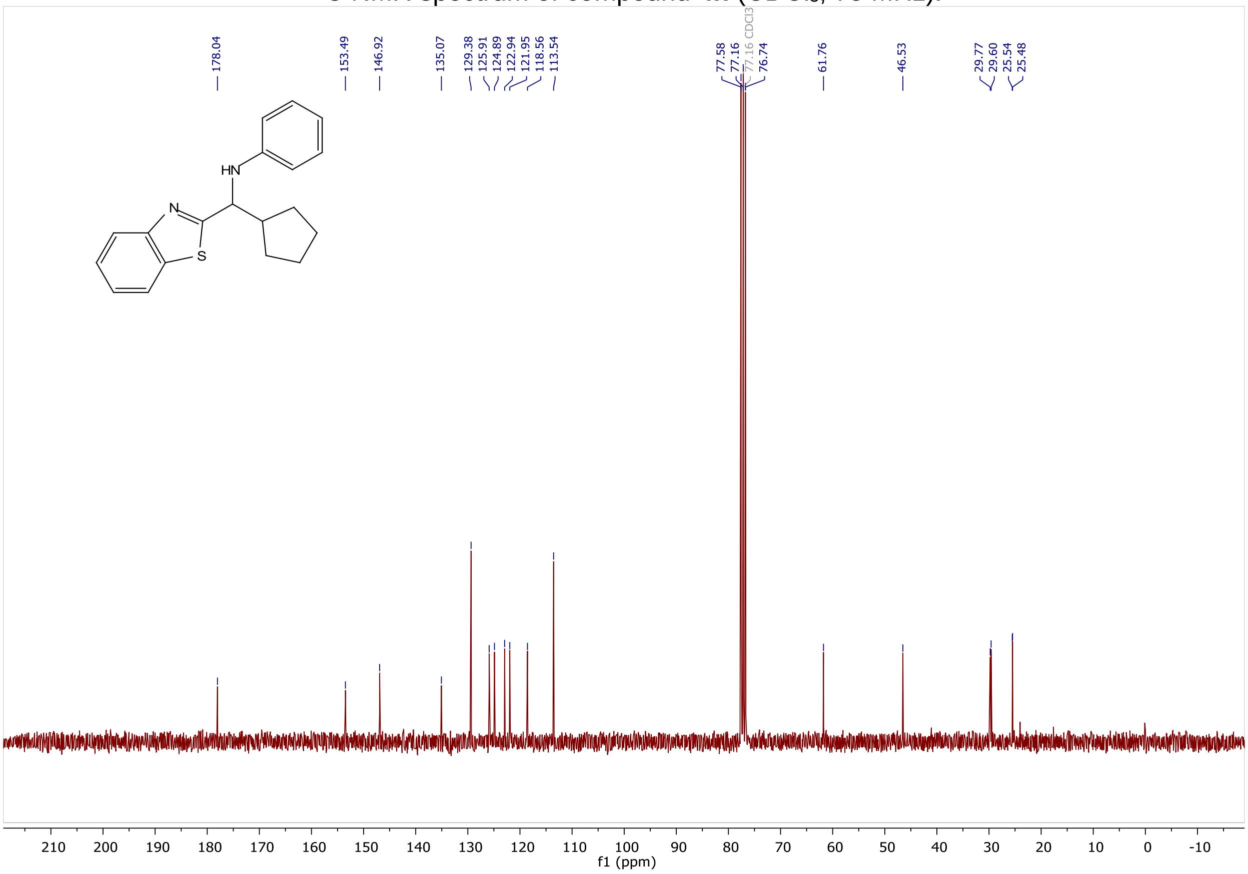

^{1}H NMR spectrum of compound **4i** ($CDCl_3$, 300 MHz).

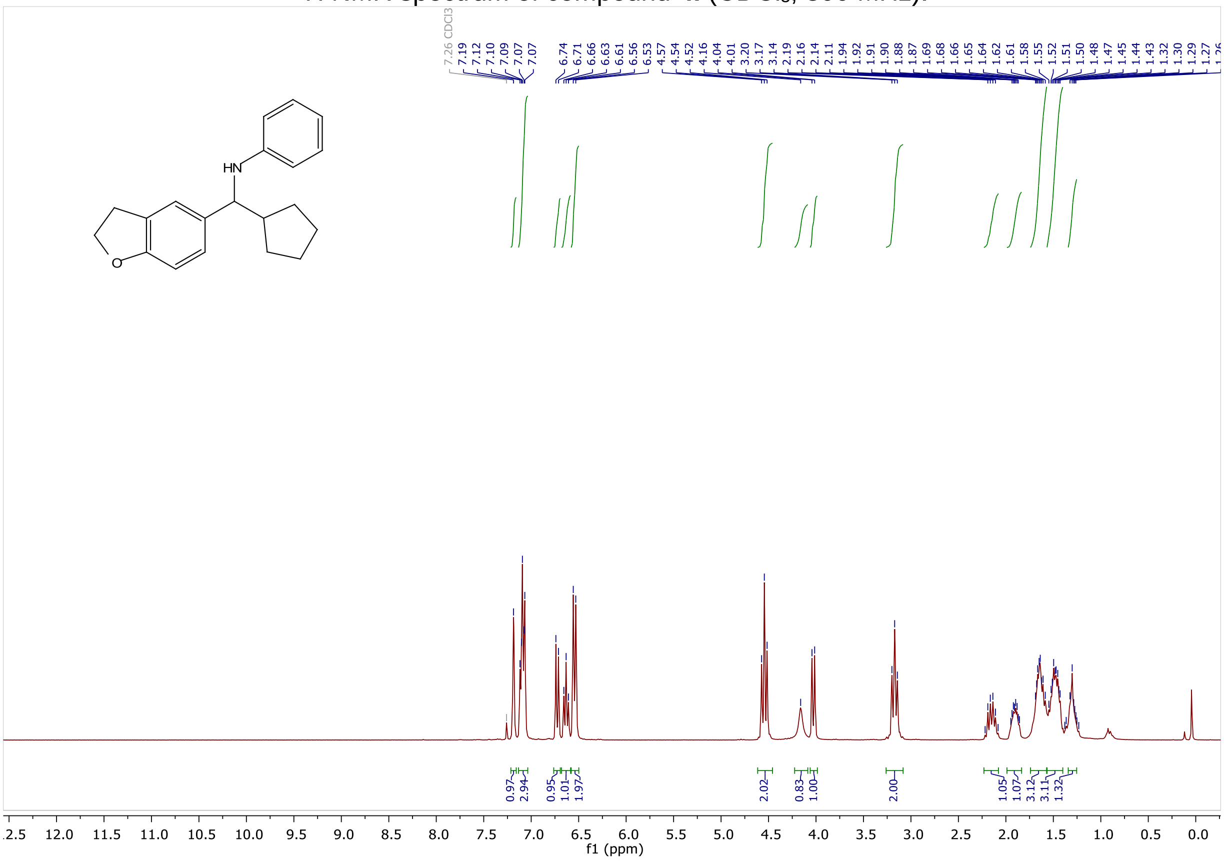

^{13}C NMR spectrum of compound **4i** ($CDCl_3$, 75 MHz).

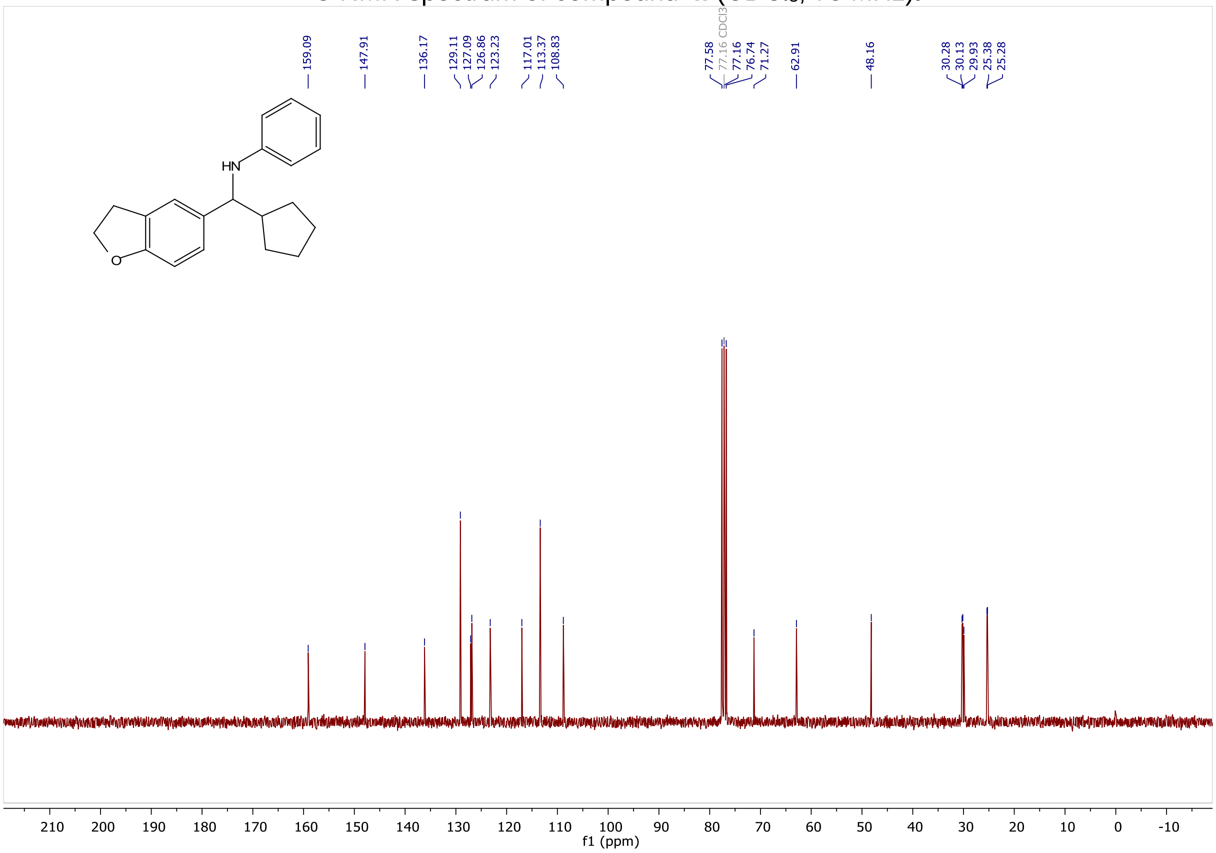

^{1}H NMR spectrum of compound **4j** ($CDCl_3$, 400 MHz).

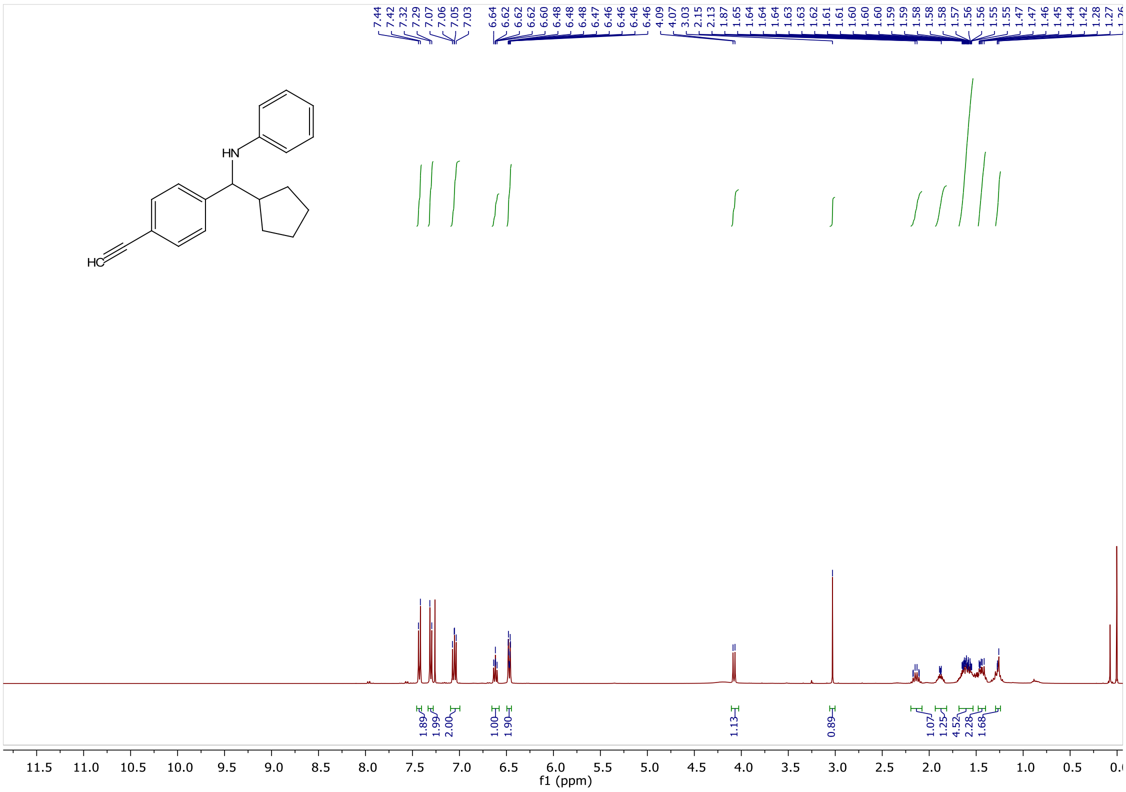

^{13}C NMR spectrum of compound **4j** ($CDCl_3$, 151 MHz).

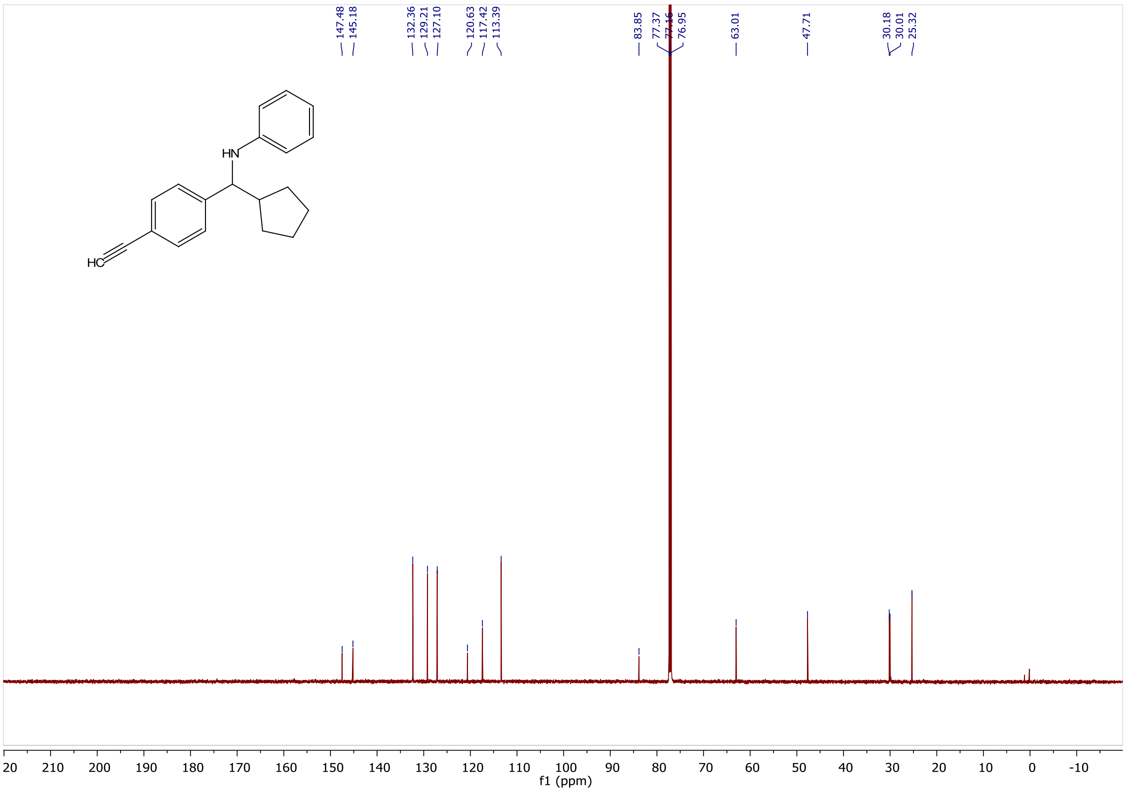

^{1}H NMR spectrum of compound **4k** ($CDCl_3$, 400 MHz).

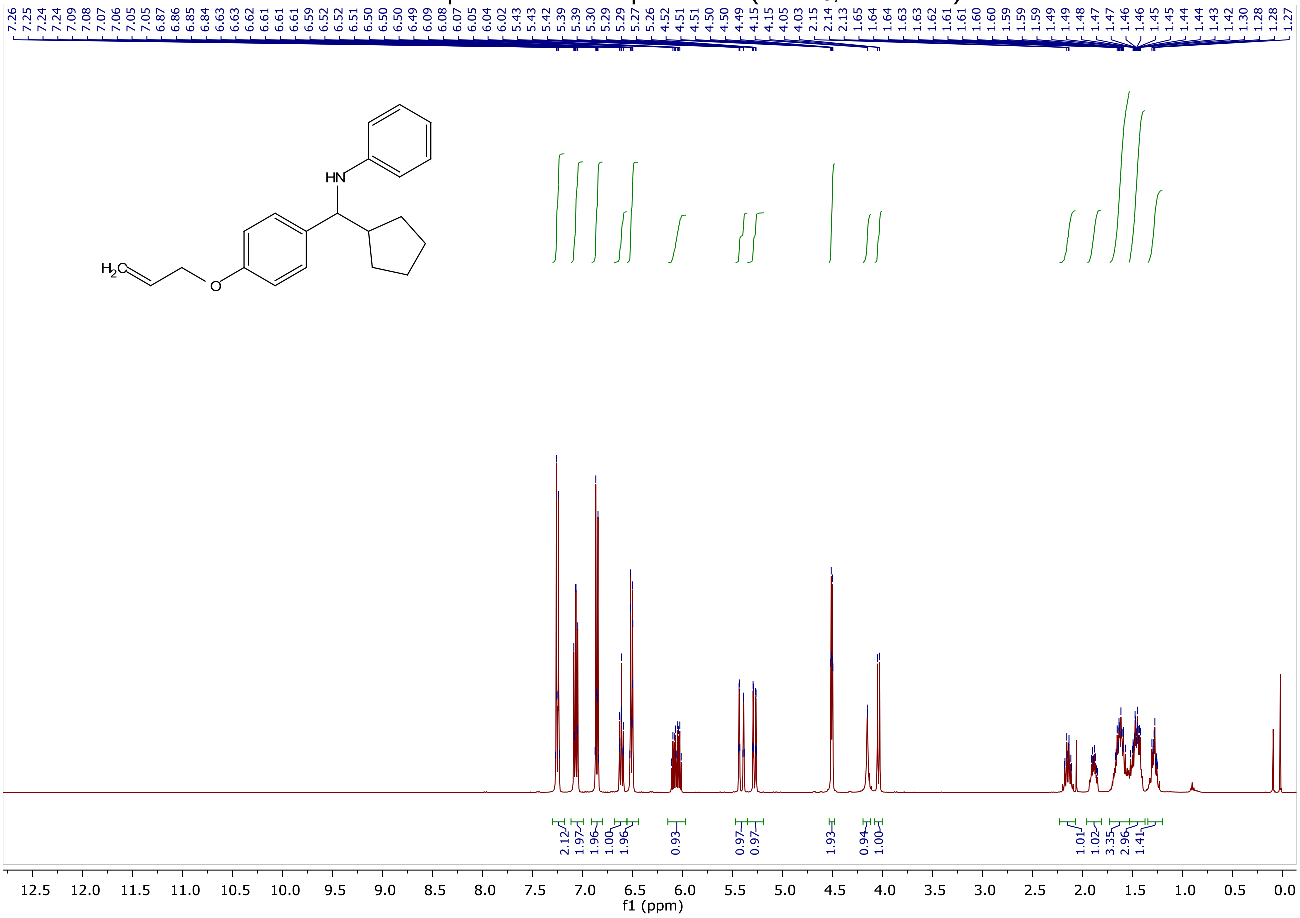

^{13}C NMR spectrum of compound **4k** ($CDCl_3$, 101 MHz).

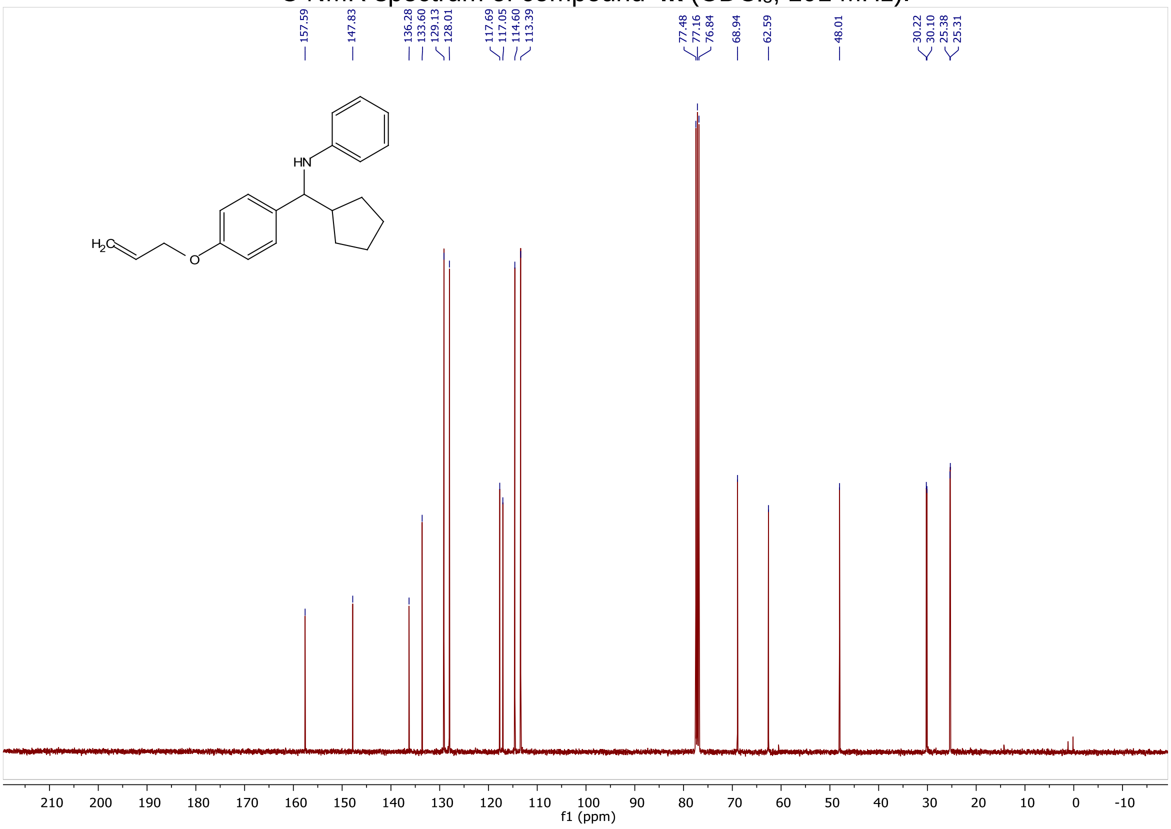

^{1}H NMR spectrum of compound **5a** ($CDCl_3$, 400 MHz).

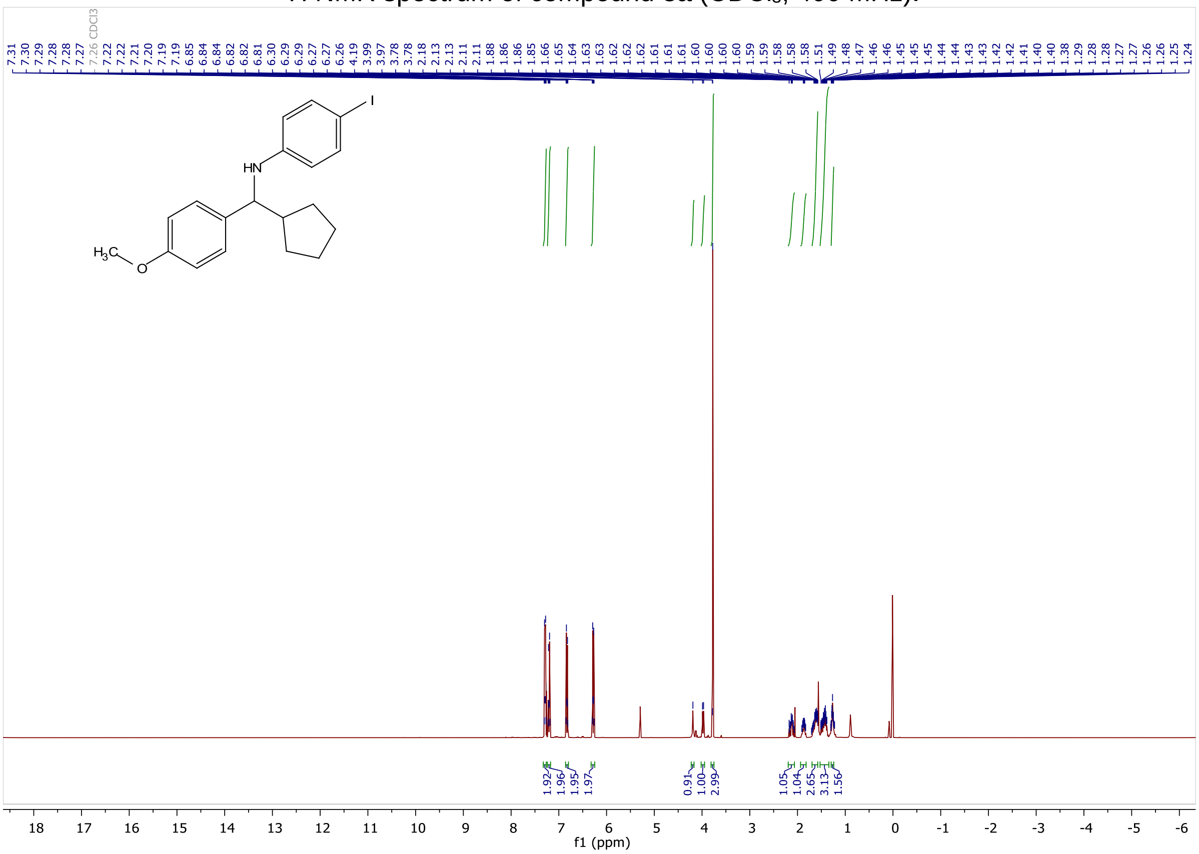

^{13}C NMR spectrum of compound **5a** ($CDCl_3$, 101 MHz).

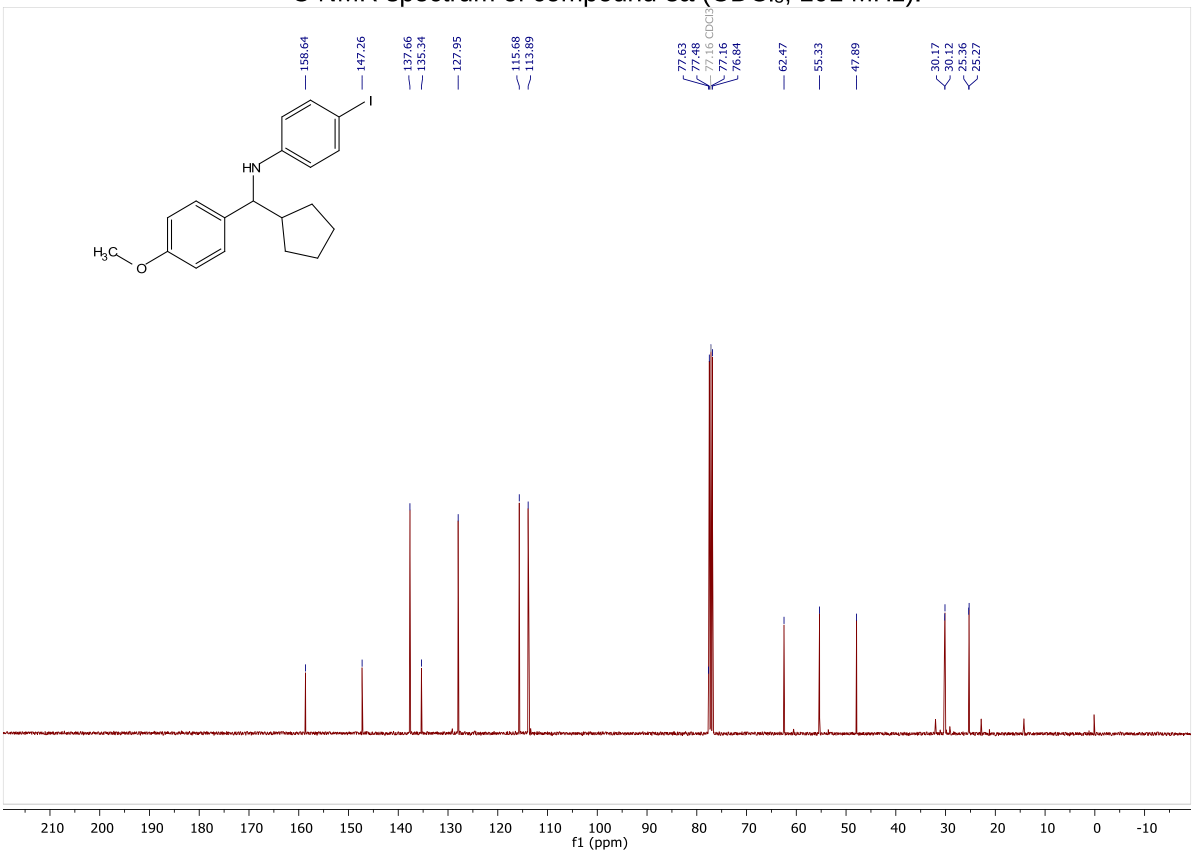

^{1}H NMR spectrum of compound **5b** ($CDCl_3$, 300 MHz).

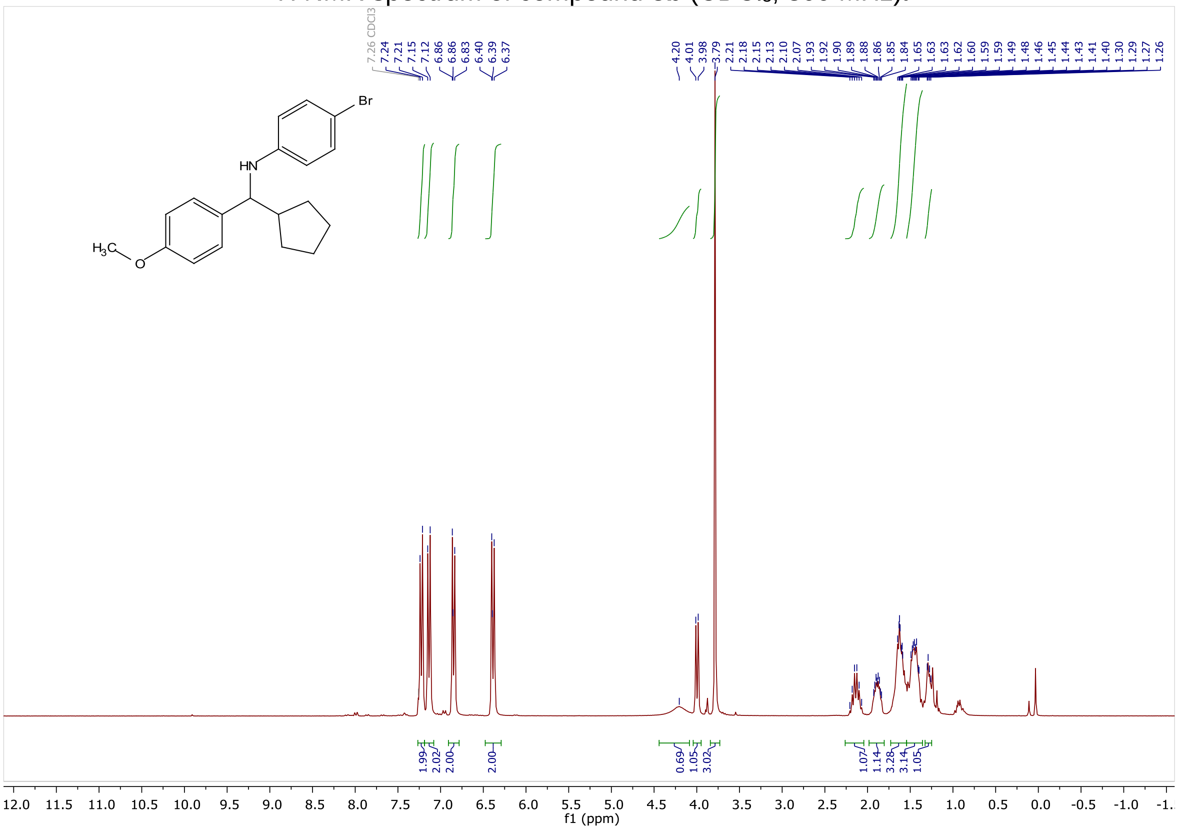

^{13}C NMR spectrum of compound **5b** ($CDCl_3$, 75 MHz).

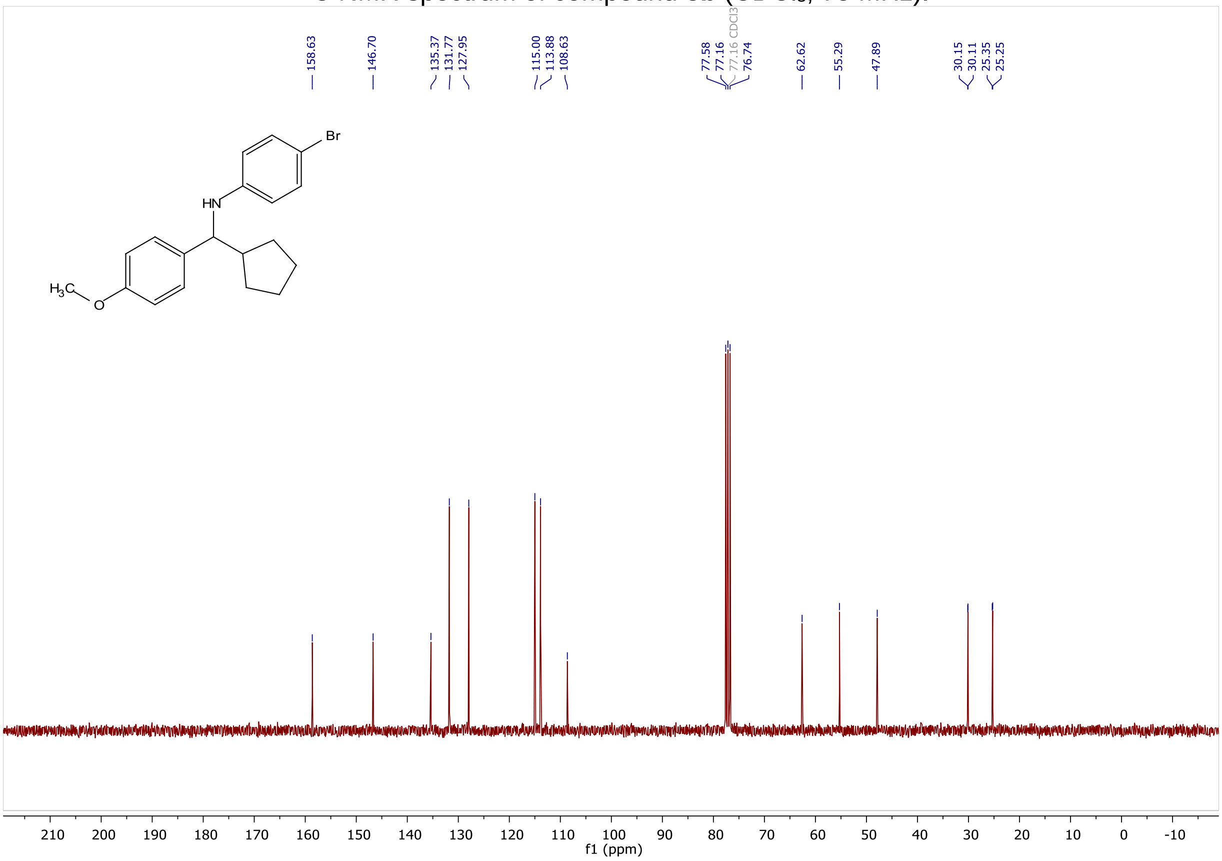

^{1}H NMR spectrum of compound **5c** ($CDCl_3$, 400 MHz).

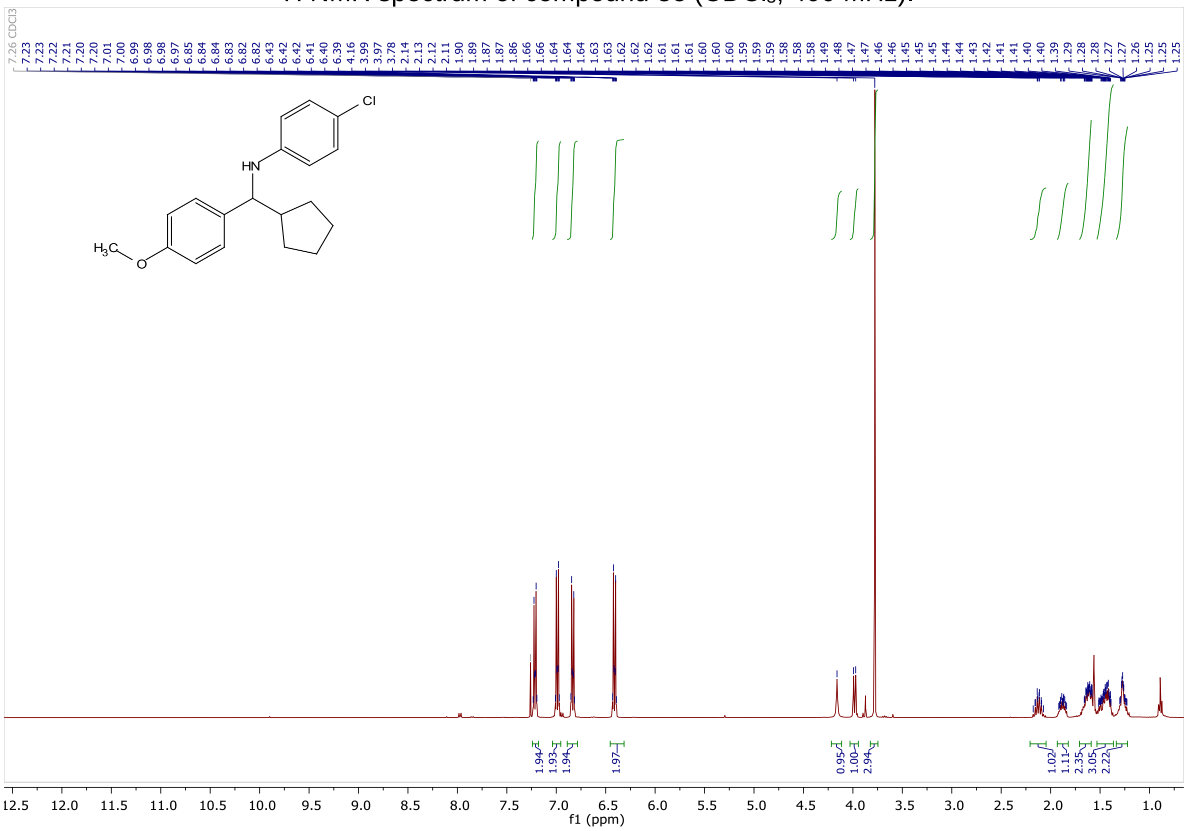

^{13}C NMR spectrum of compound **5c** ($CDCl_3$, 101 MHz).

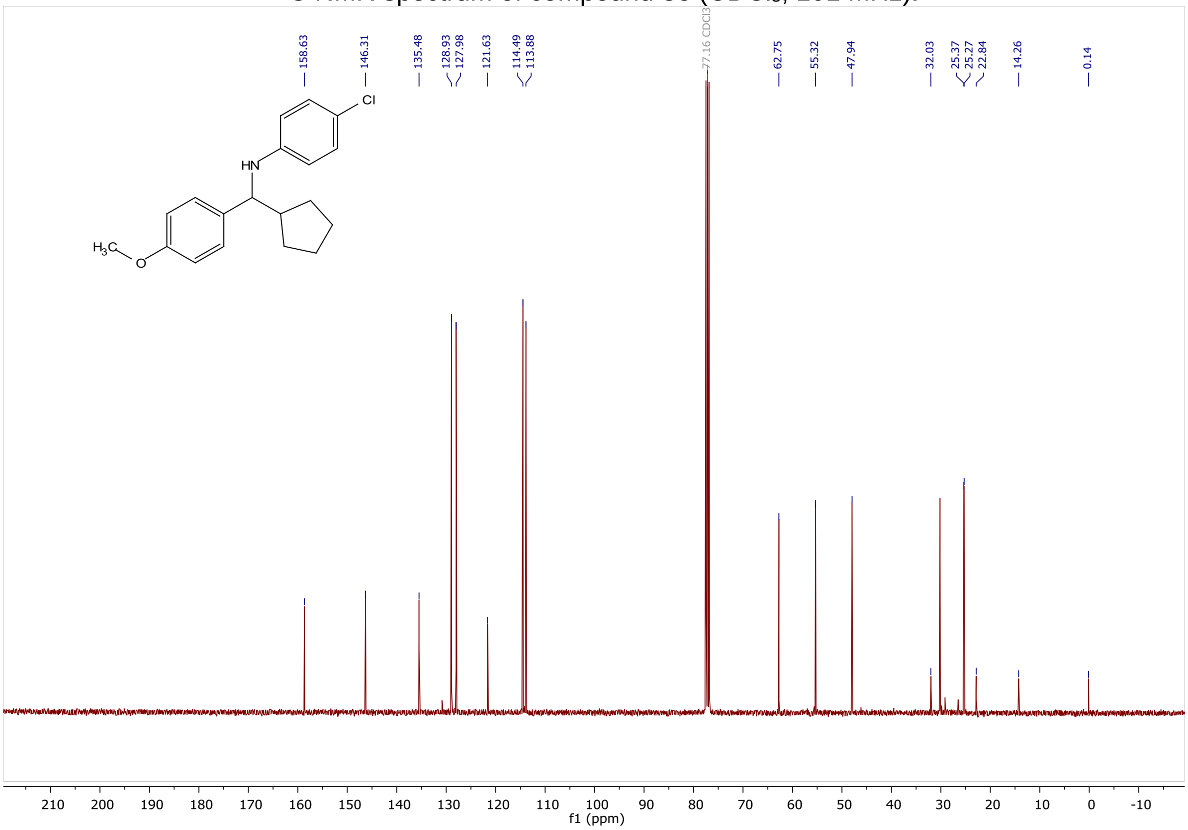

^{1}H NMR spectrum of compound **5d** ($CDCl_3$, 300 MHz).

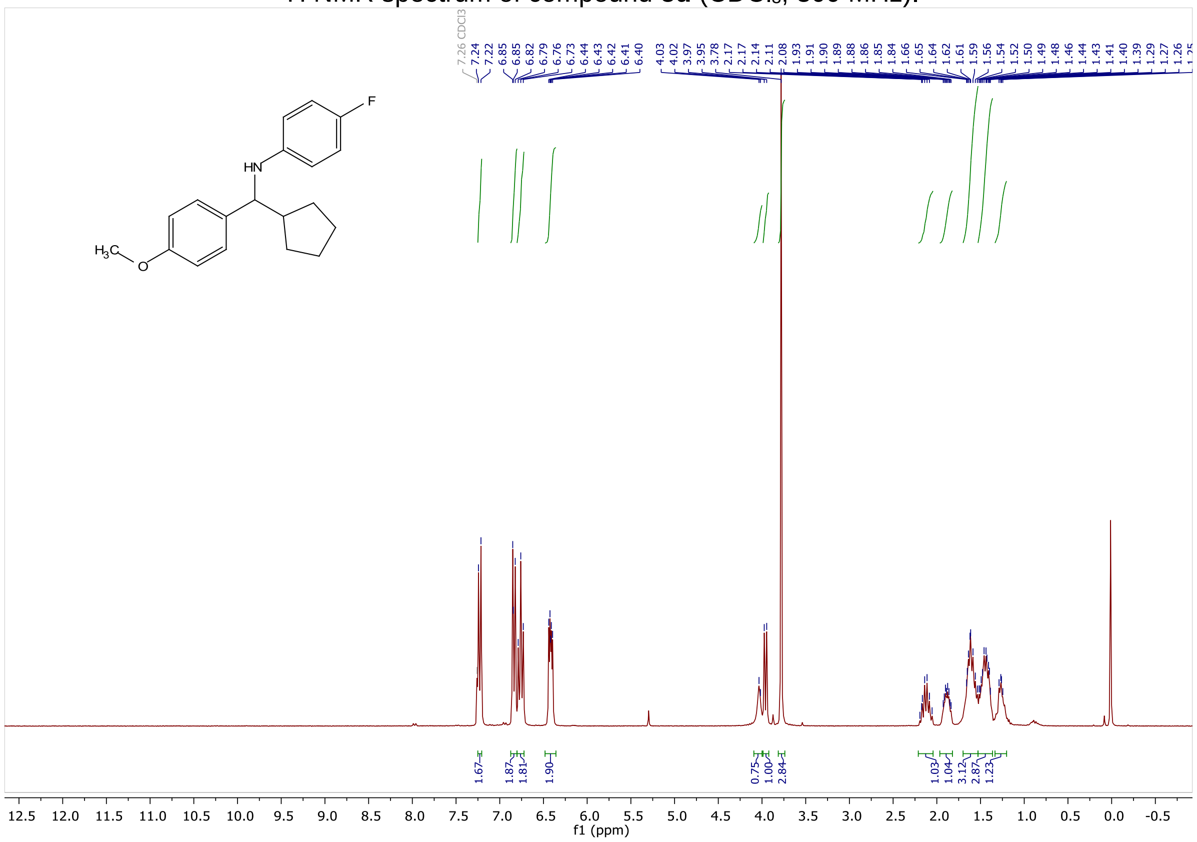

^{13}C NMR spectrum of compound **5d** ($CDCl_3$, 75 MHz).

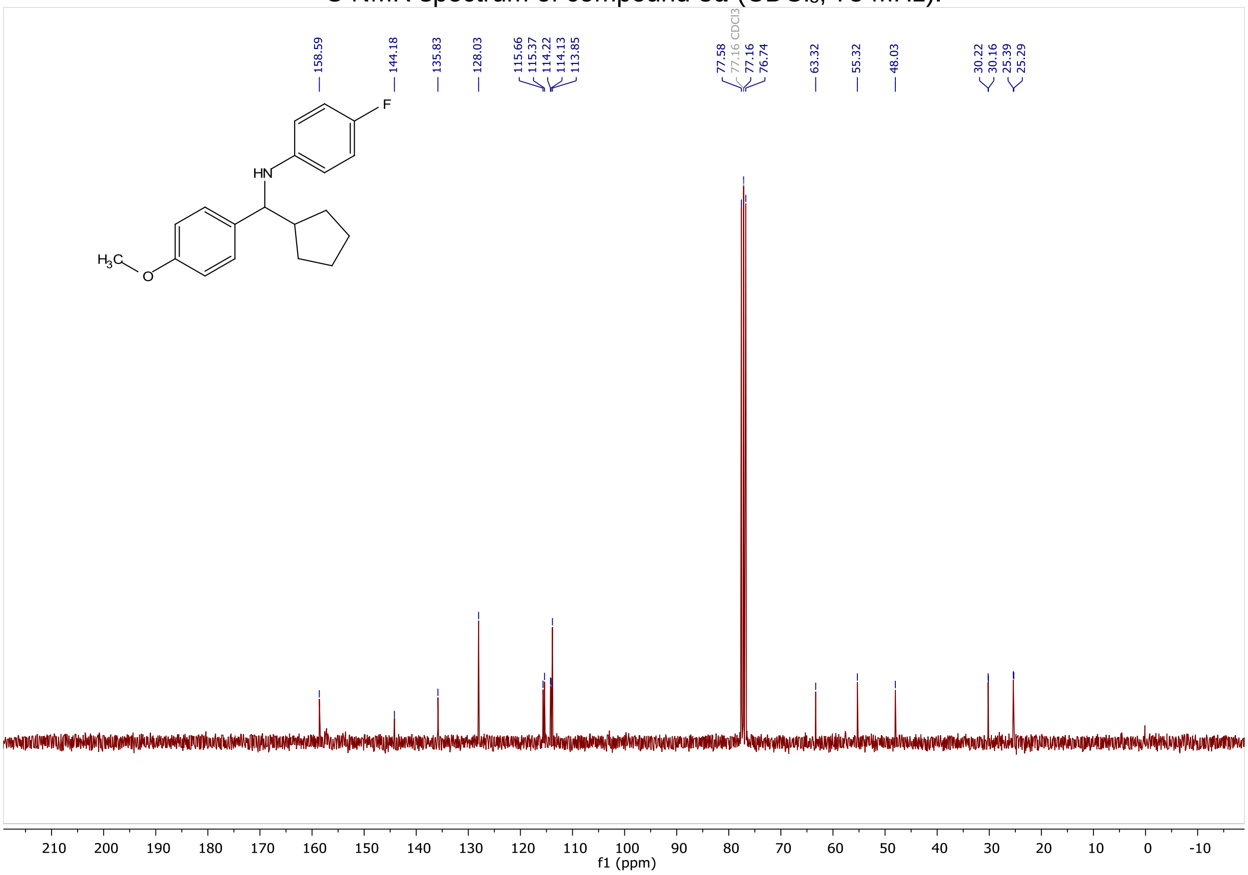

^{1}H NMR spectrum of compound **5e** ($CDCl_3$, 400 MHz).

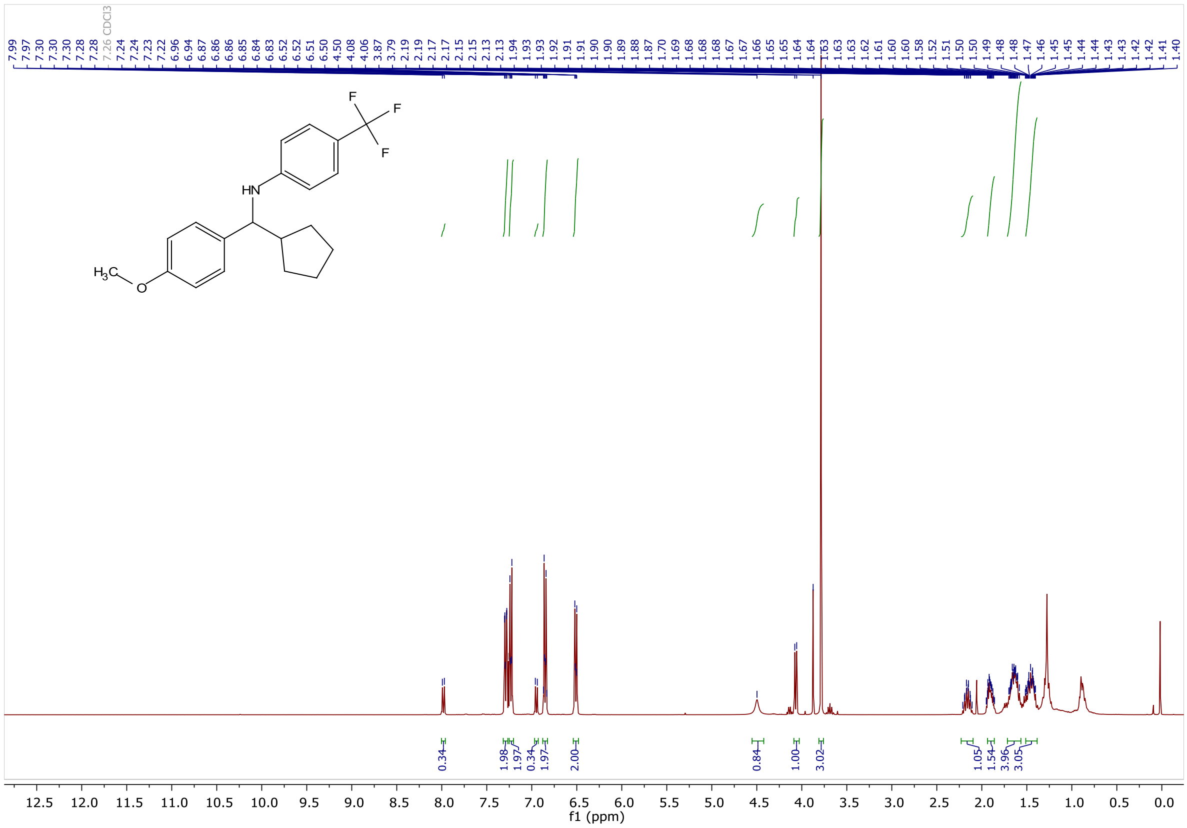

^{13}C NMR spectrum of compound **5e** ($CDCl_3$, 101 MHz).

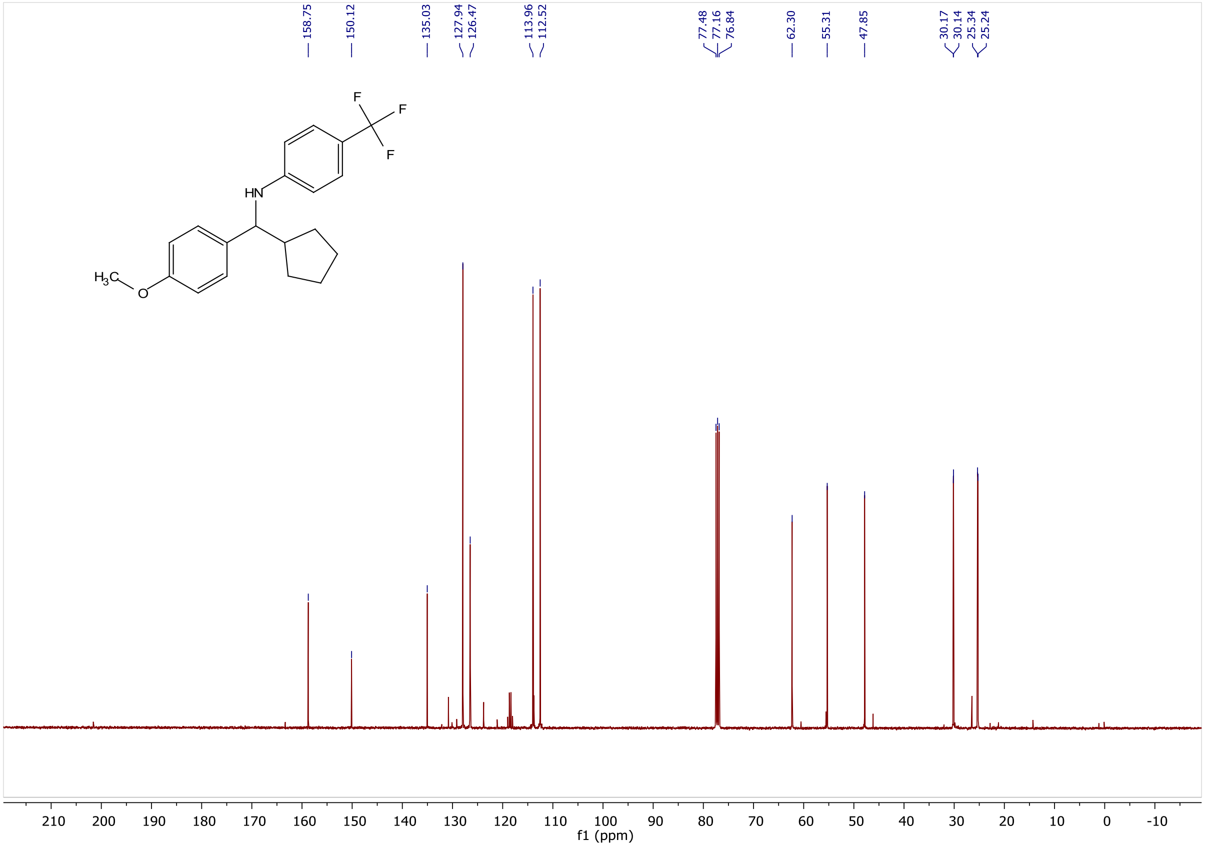

^{1}H NMR spectrum of compound **5f** ($CDCl_3$, 300 MHz).

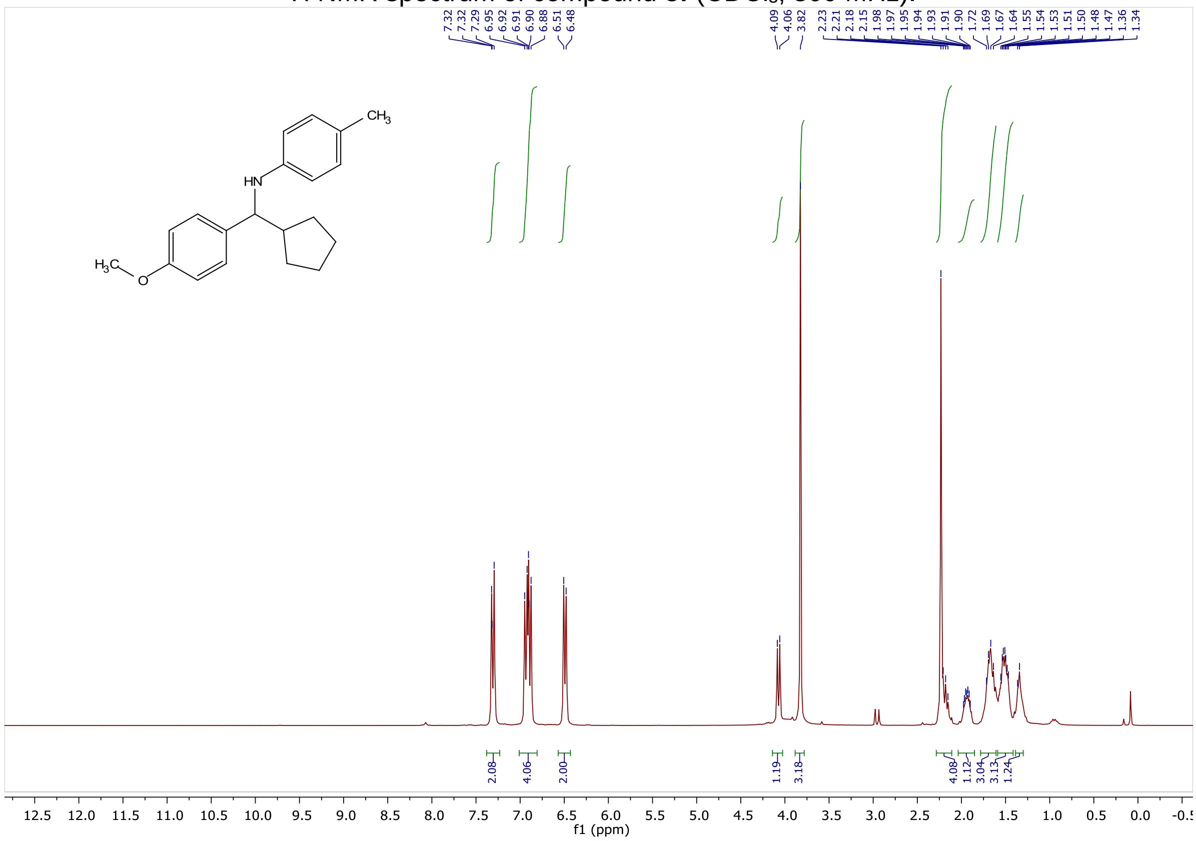

^{13}C NMR spectrum of compound **5f** ($CDCl_3$, 75 MHz).

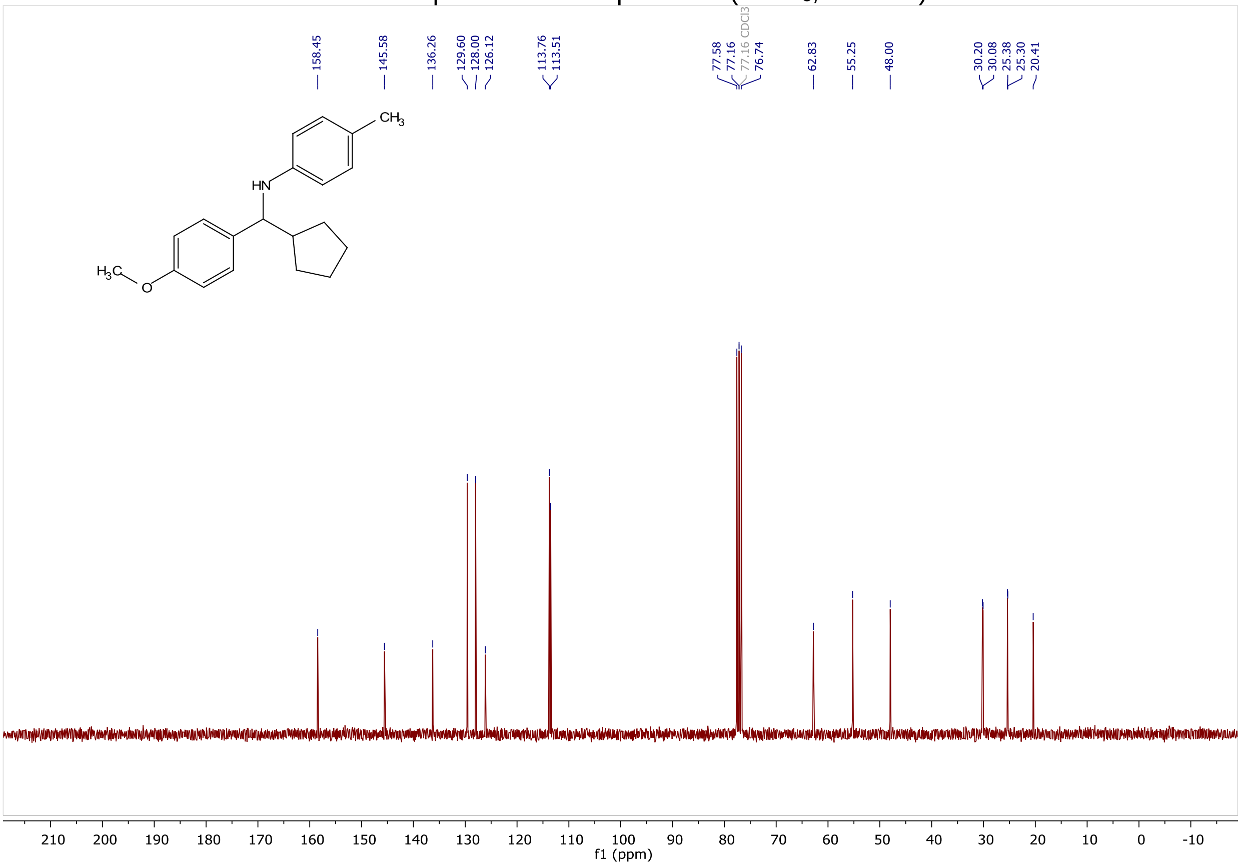

^{1}H NMR spectrum of compound **5g** ($CDCl_3$, 300 MHz).

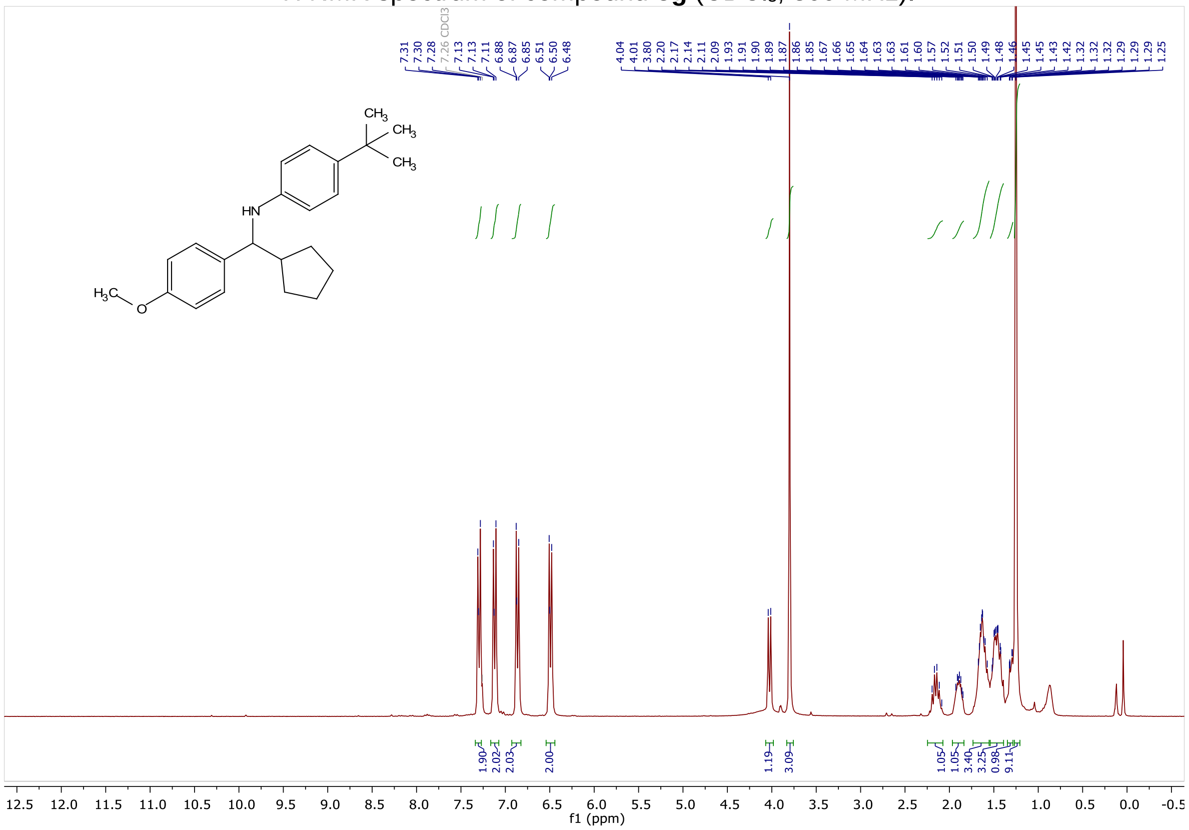

^{13}C NMR spectrum of compound **5g** ($CDCl_3$, 75 MHz).

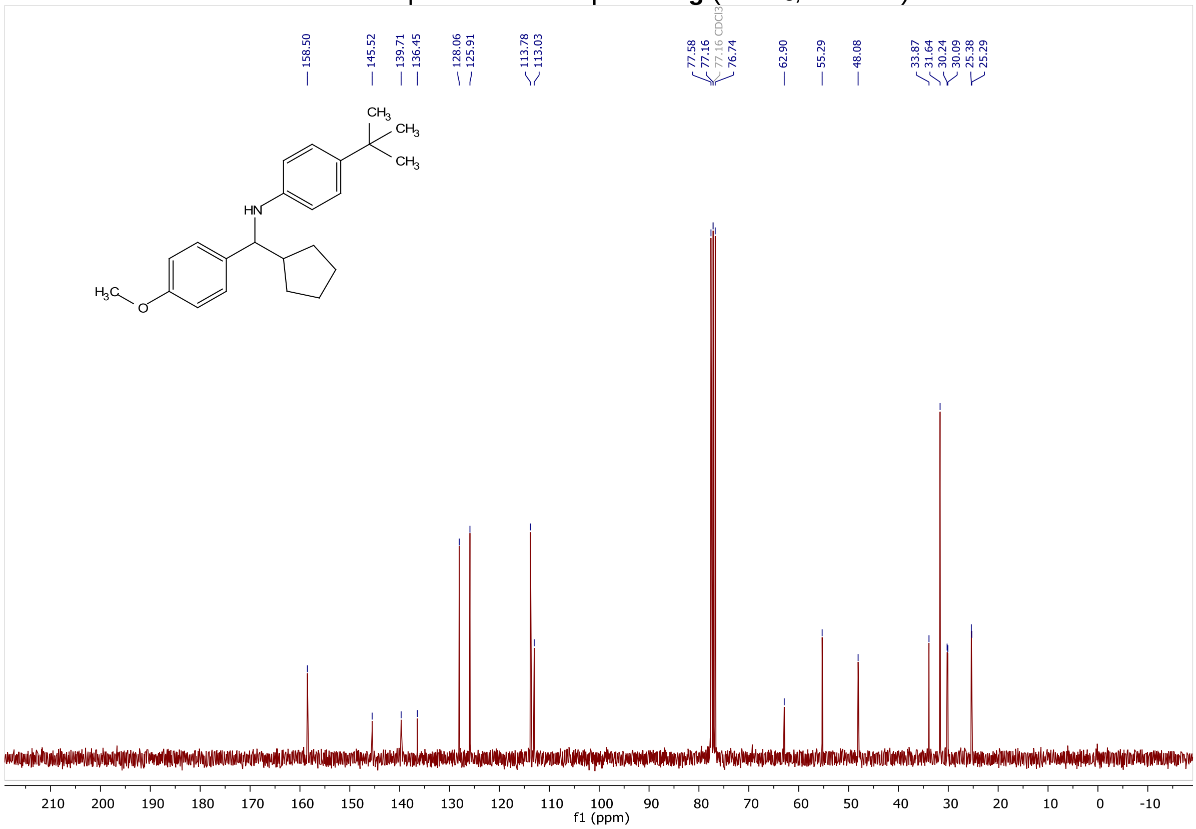

^{1}H NMR spectrum of compound **5h** ($CDCl_3$, 300 MHz).

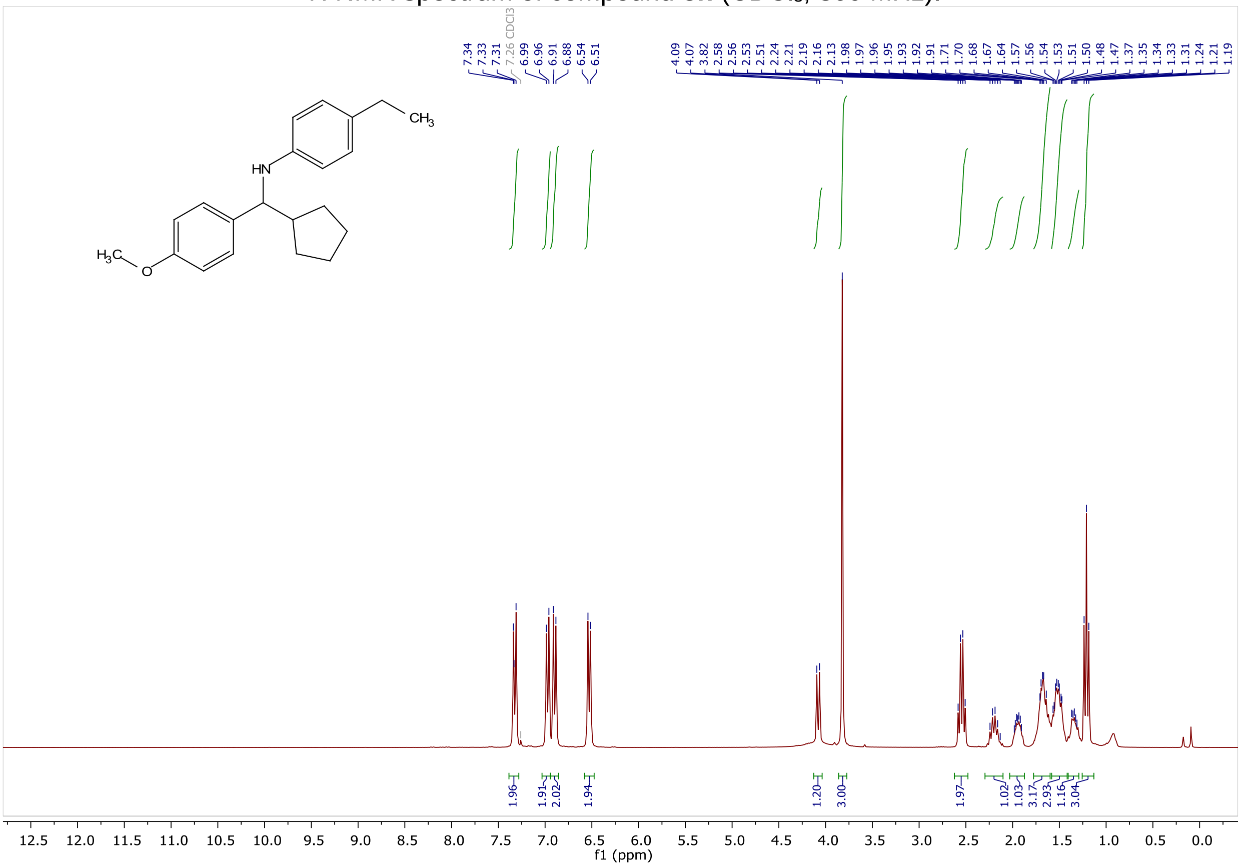

^{13}C NMR spectrum of compound **5h** ($CDCl_3$, 75 MHz).

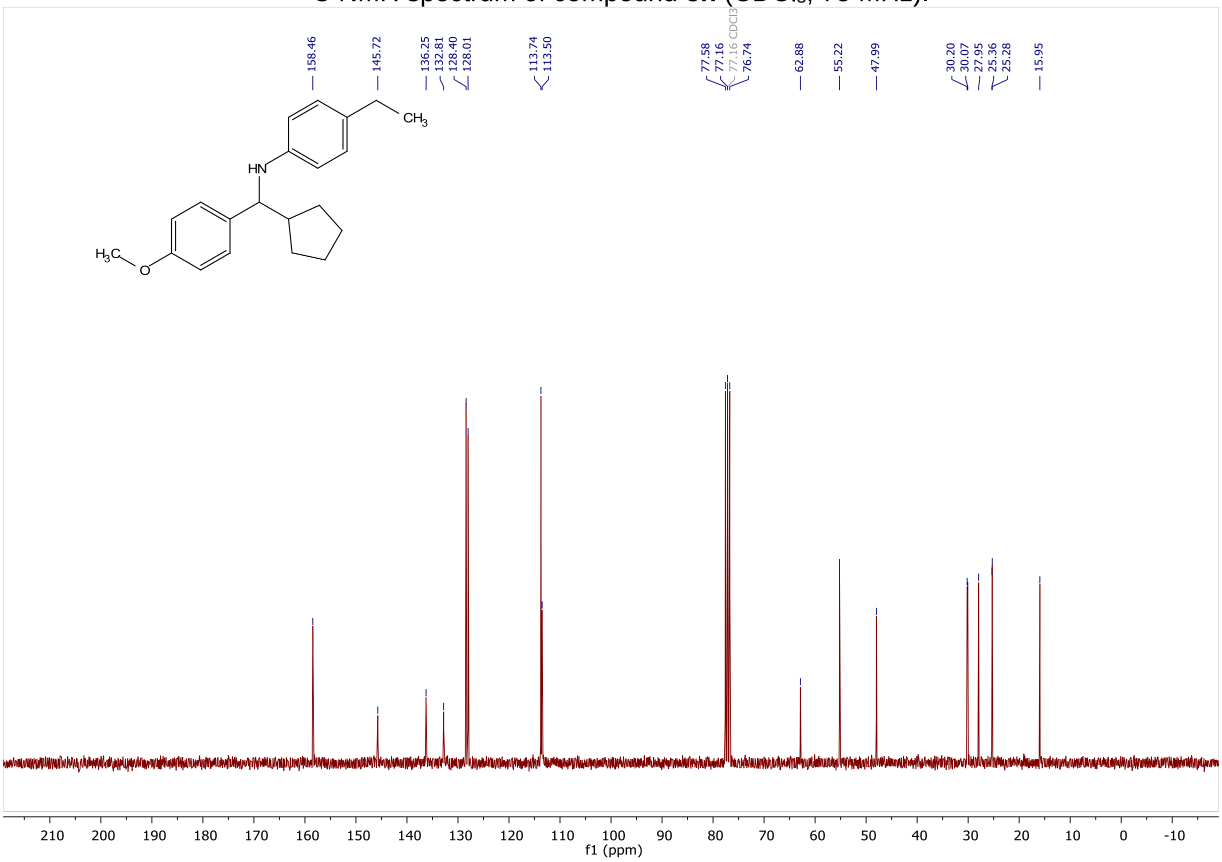

^{1}H NMR spectrum of compound **5i** ($CDCl_3$, 300 MHz).

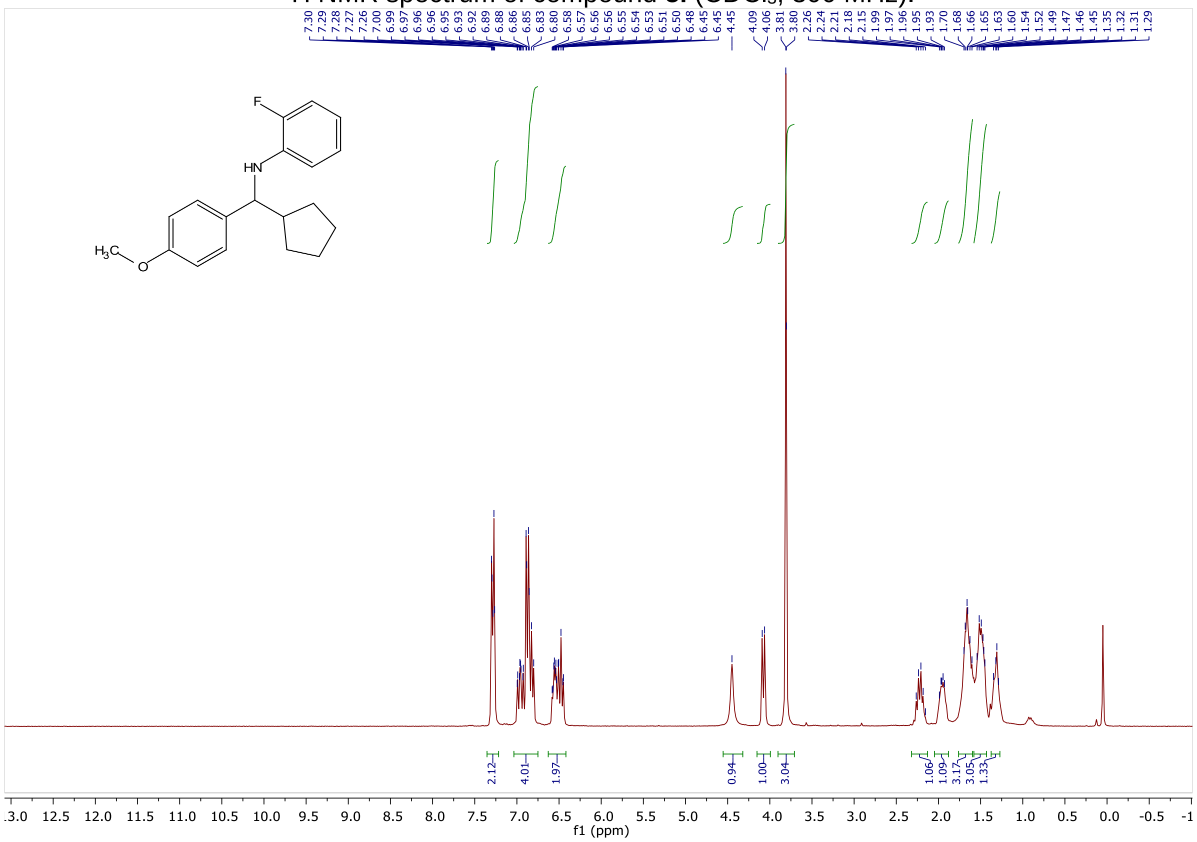

^{13}C NMR spectrum of compound **5i** ($CDCl_3$, 75 MHz).

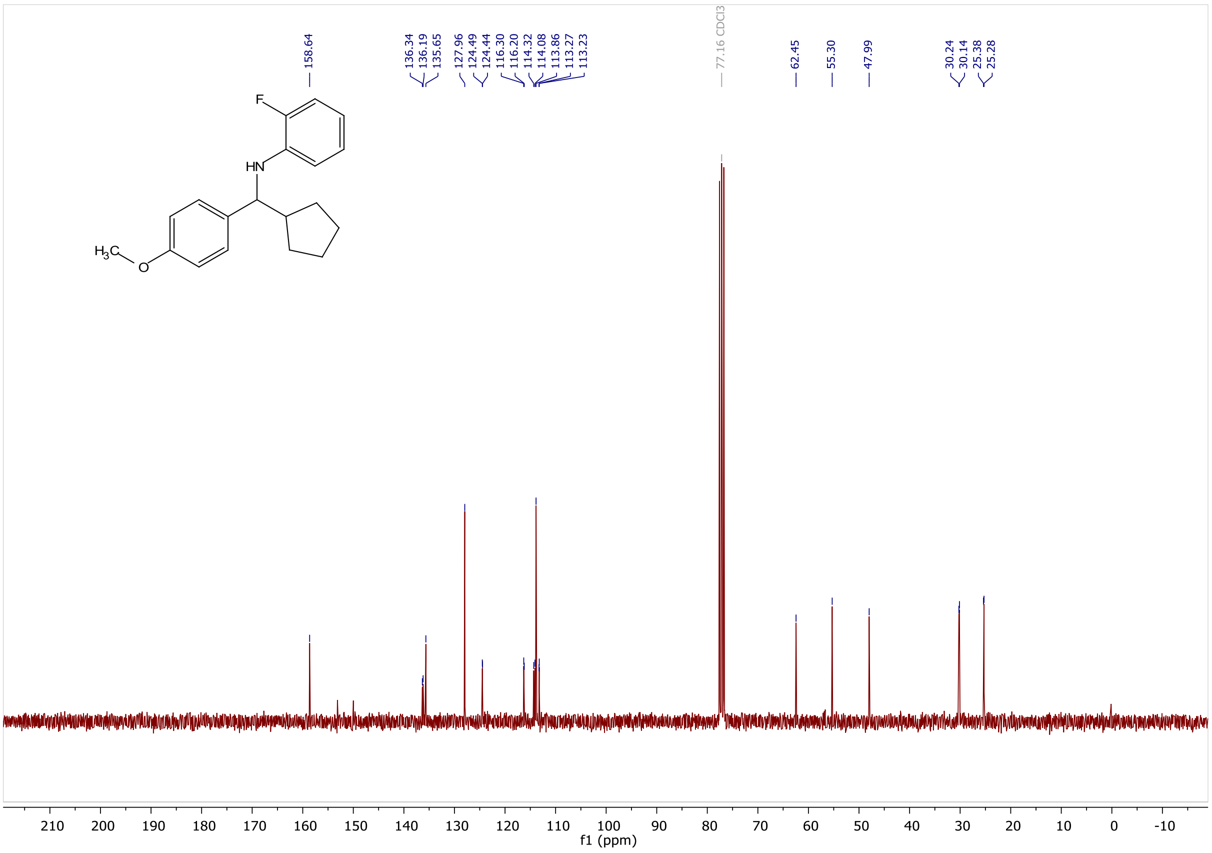

^{1}H NMR spectrum of compound **5j** ($CDCl_3$, 300 MHz).

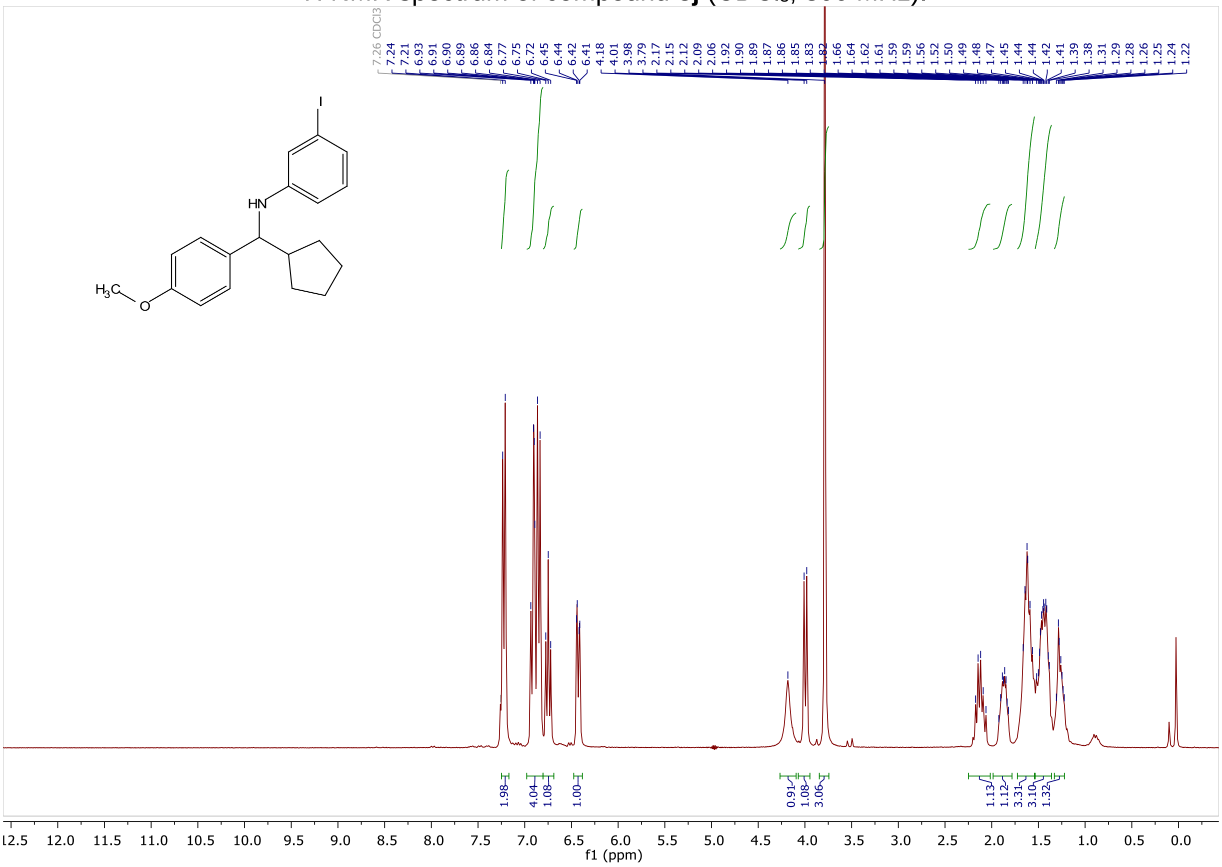

^{13}C NMR spectrum of compound **5j** ($CDCl_3$, 75 MHz).

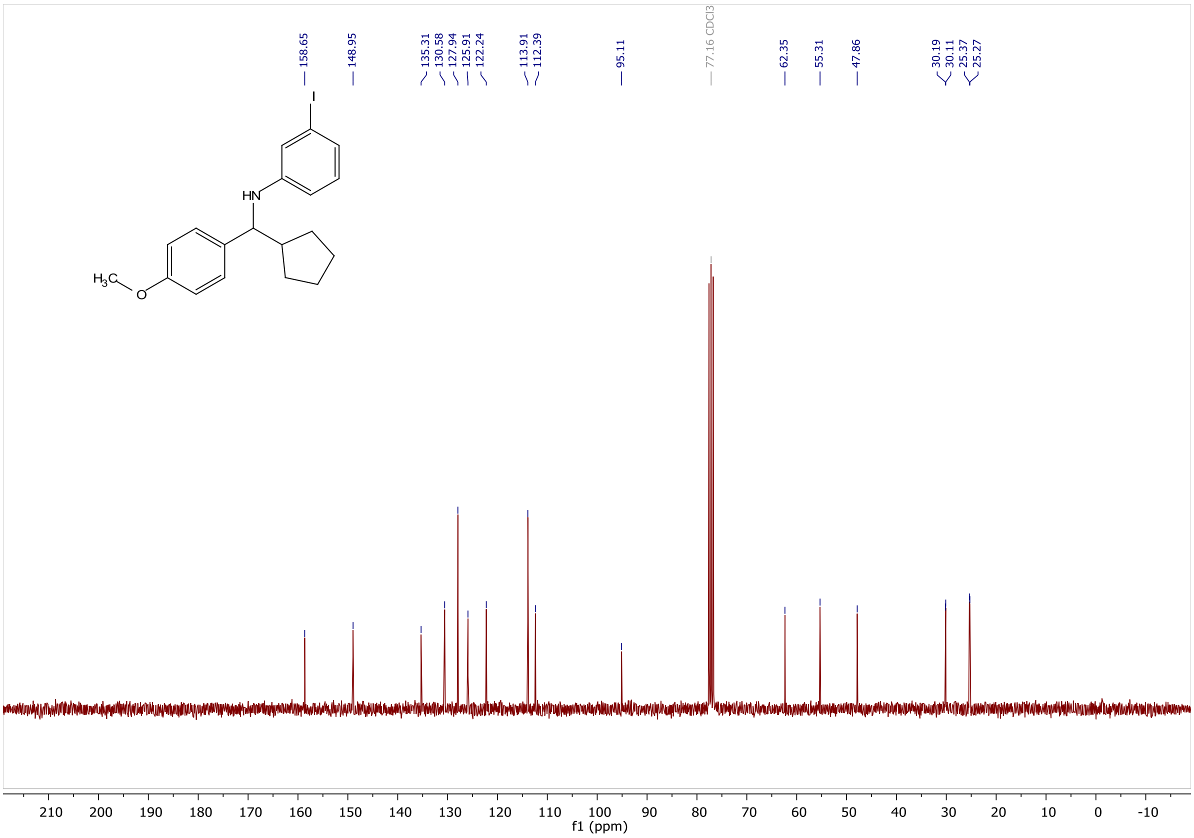

^{1}H NMR spectrum of compound **5k** ($CDCl_3$, 400 MHz).

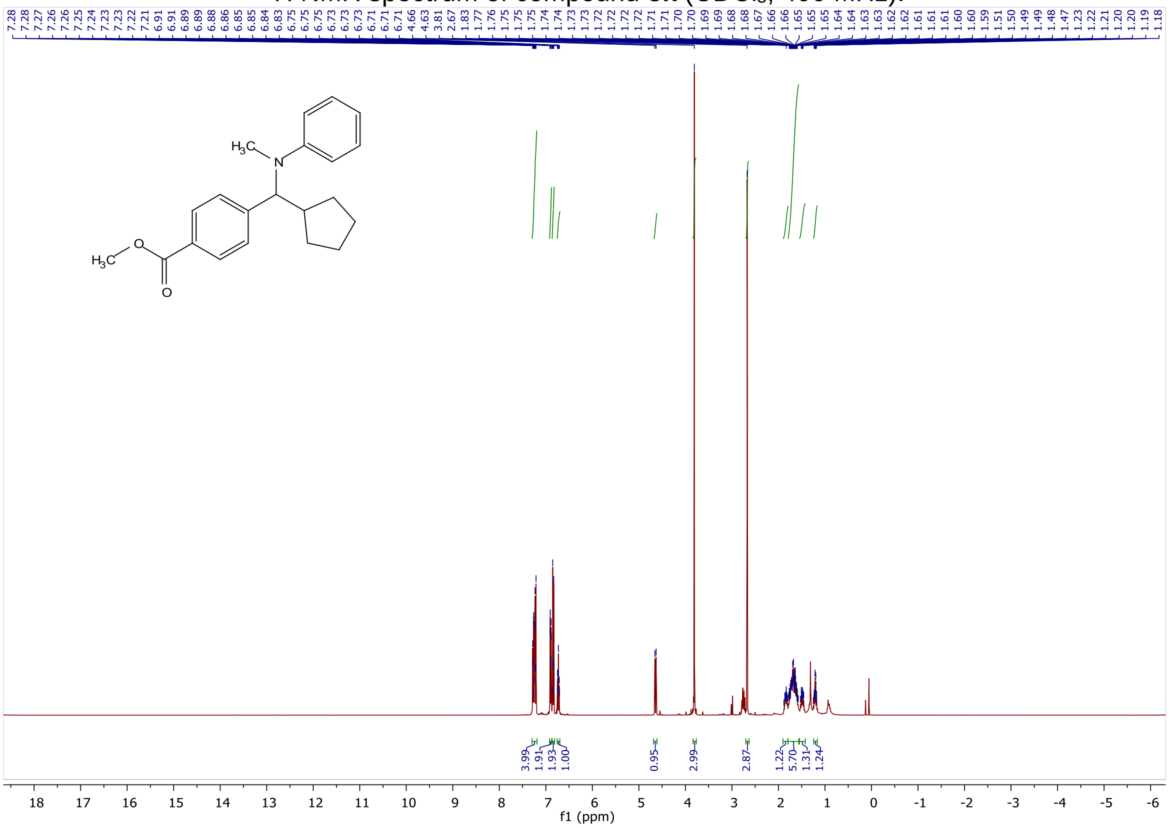

^{13}C NMR spectrum of compound **5k** ($CDCl_3$, 101 MHz).

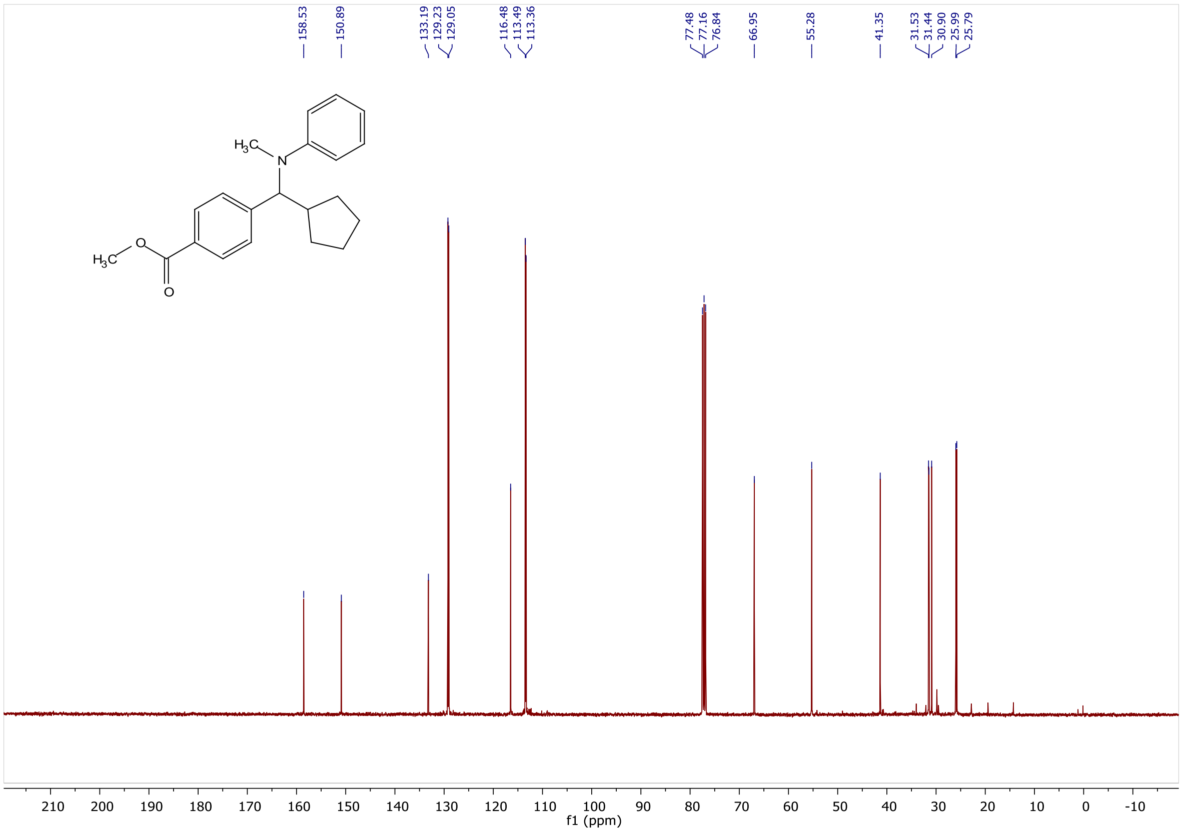

^1H NMR spectrum of compound **5l** ($CDCl_3$, 300 MHz).

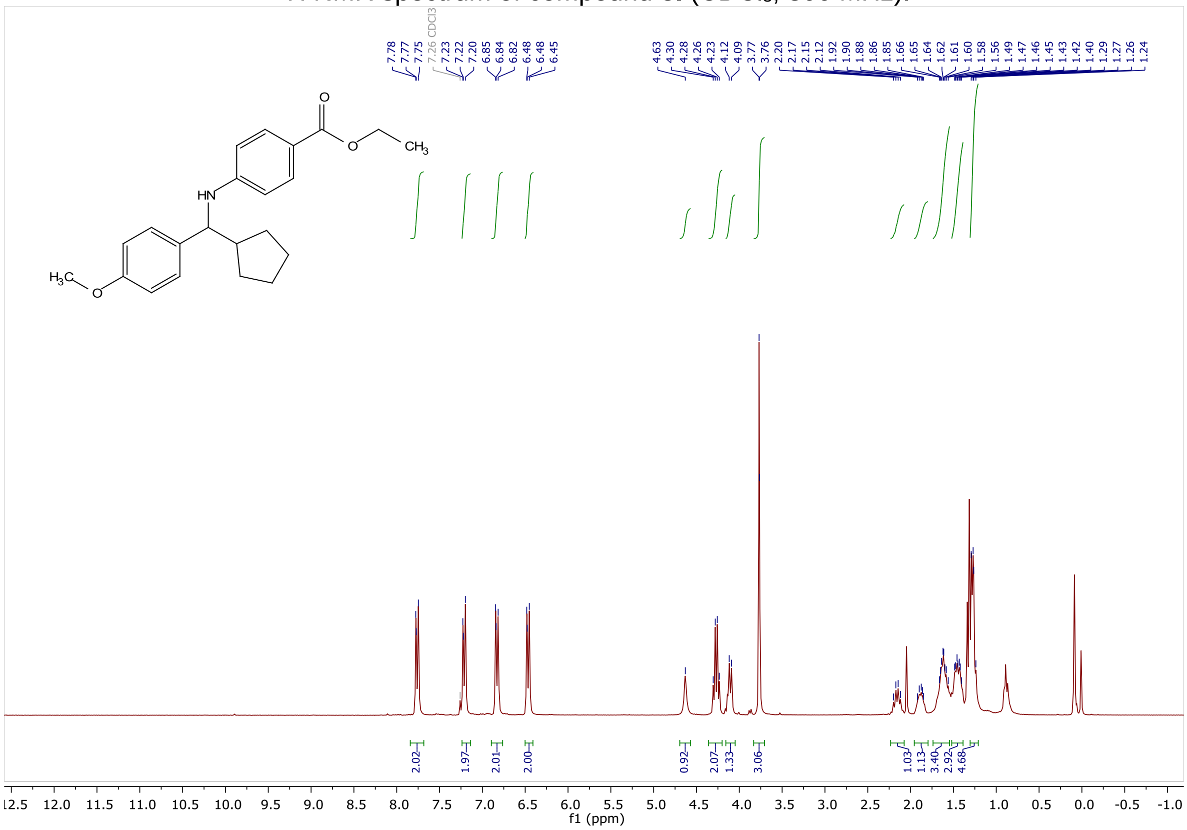

^{13}C NMR spectrum of compound **5l** ($CDCl_3$, 75 MHz).

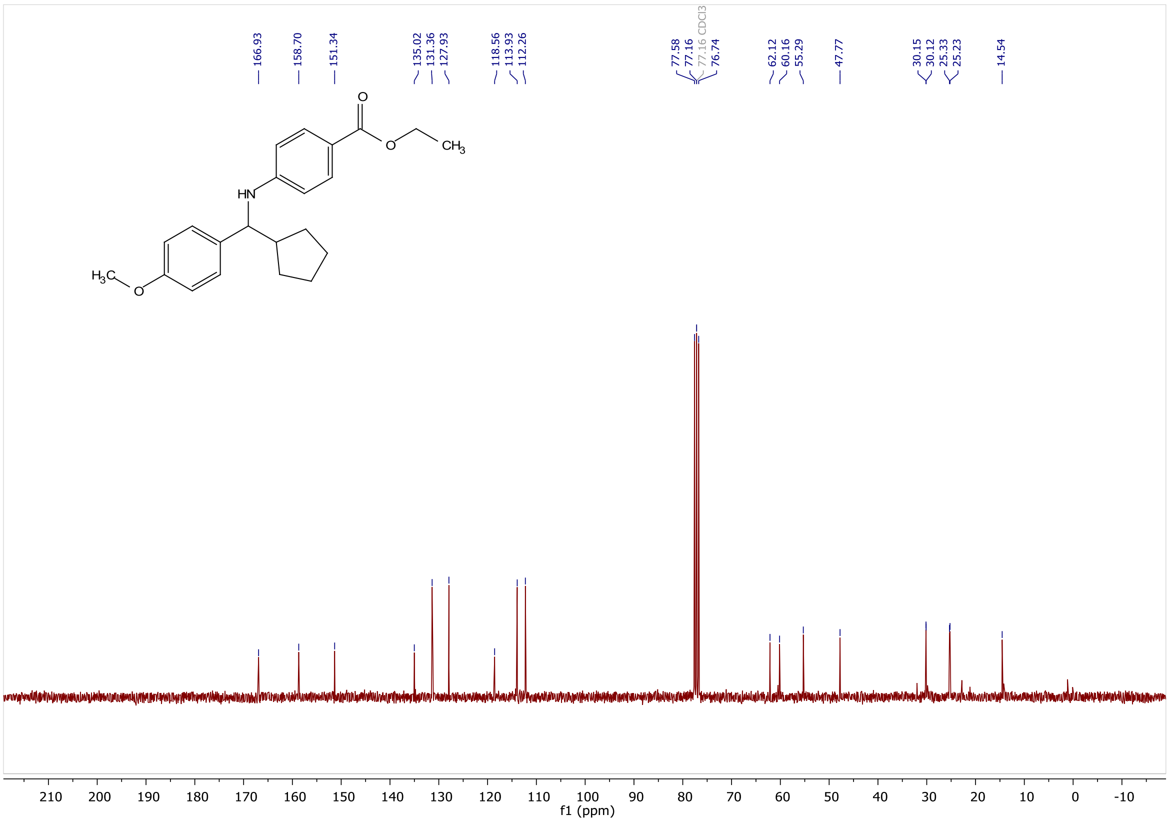

^{1}H NMR spectrum of compound **5m** ($CDCl_3$, 300 MHz).

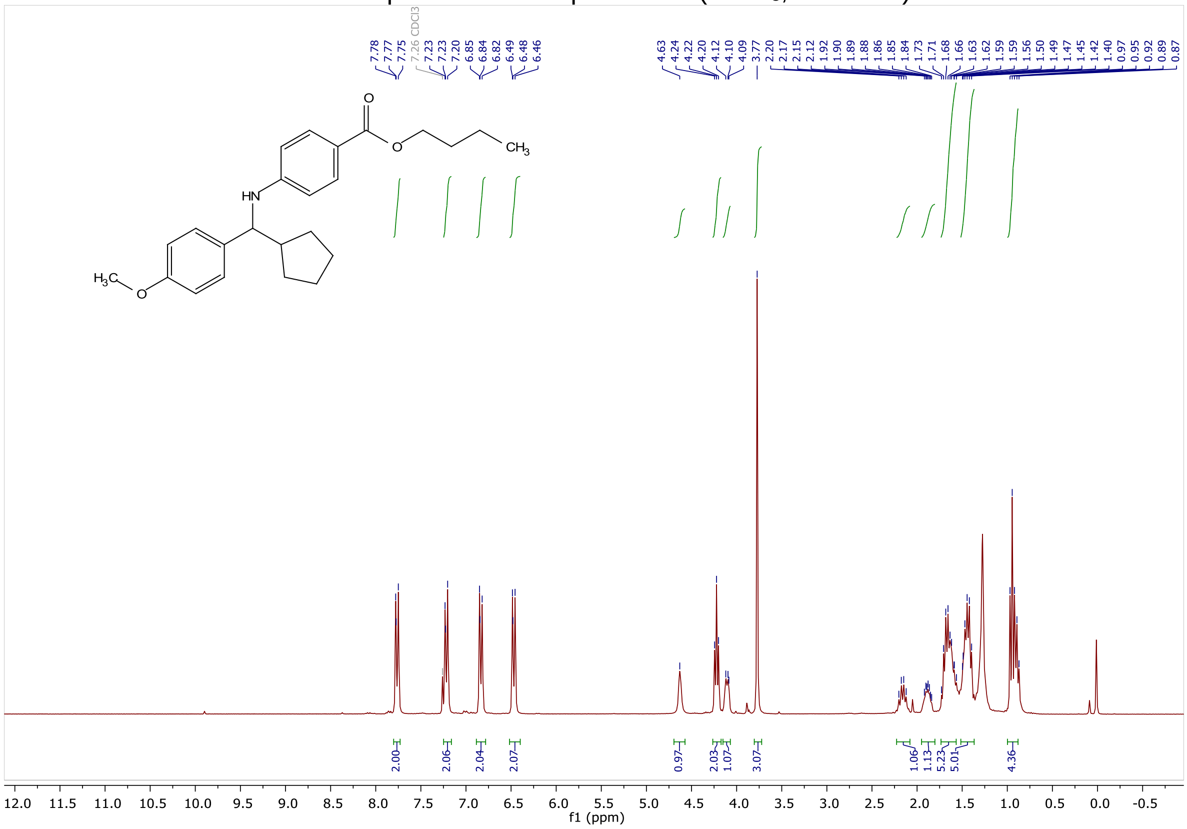

^{13}C NMR spectrum of compound **5m** ($CDCl_3$, 75 MHz).

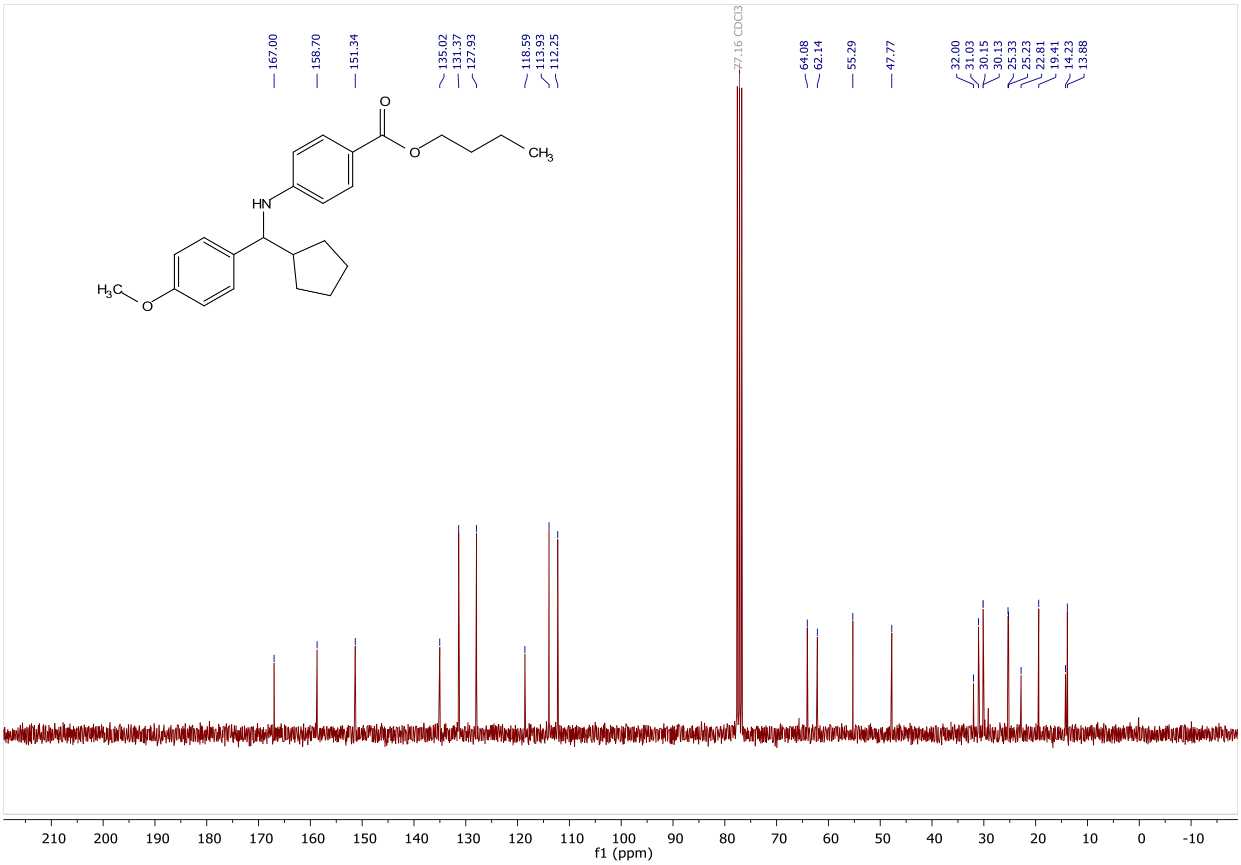

^1H NMR spectrum of compound **6a** ($CDCl_3$, 400 MHz).

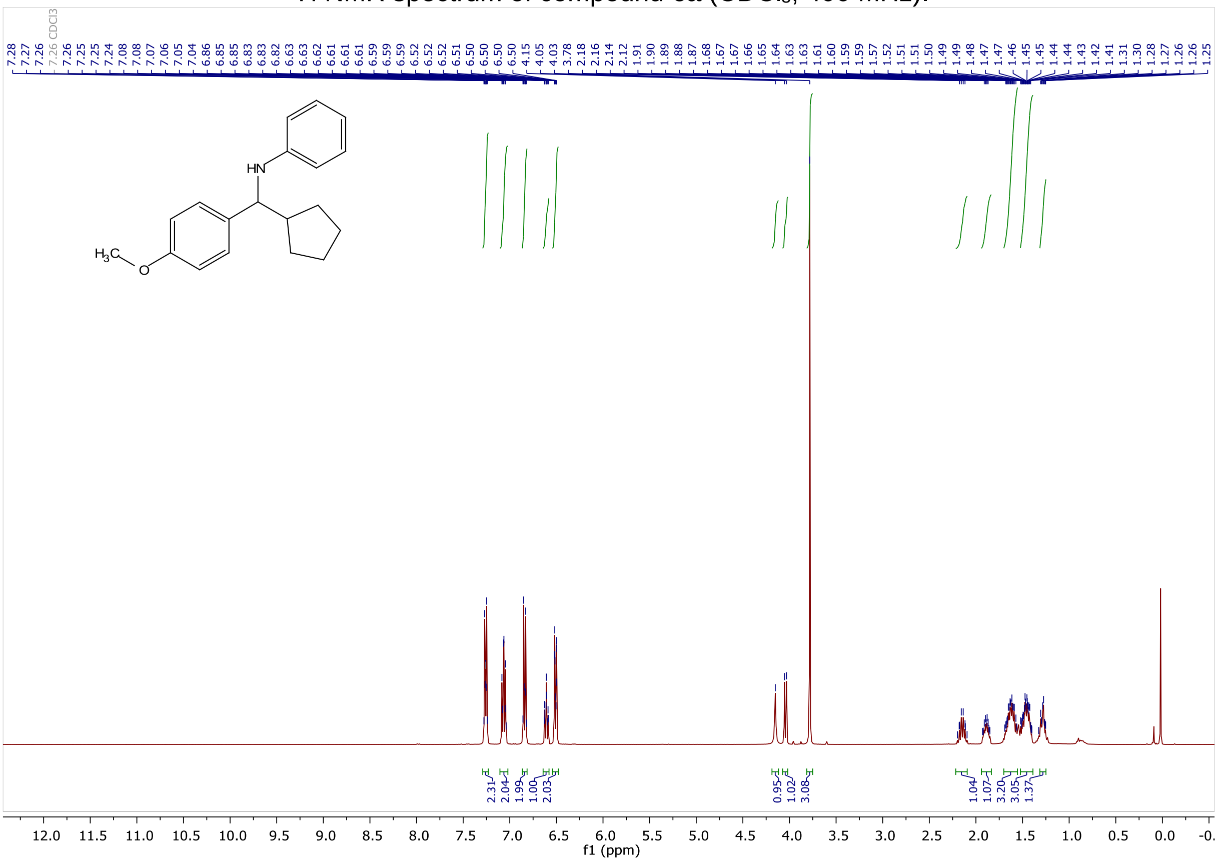

^{13}C NMR spectrum of compound **6a** ($CDCl_3$, 101 MHz).

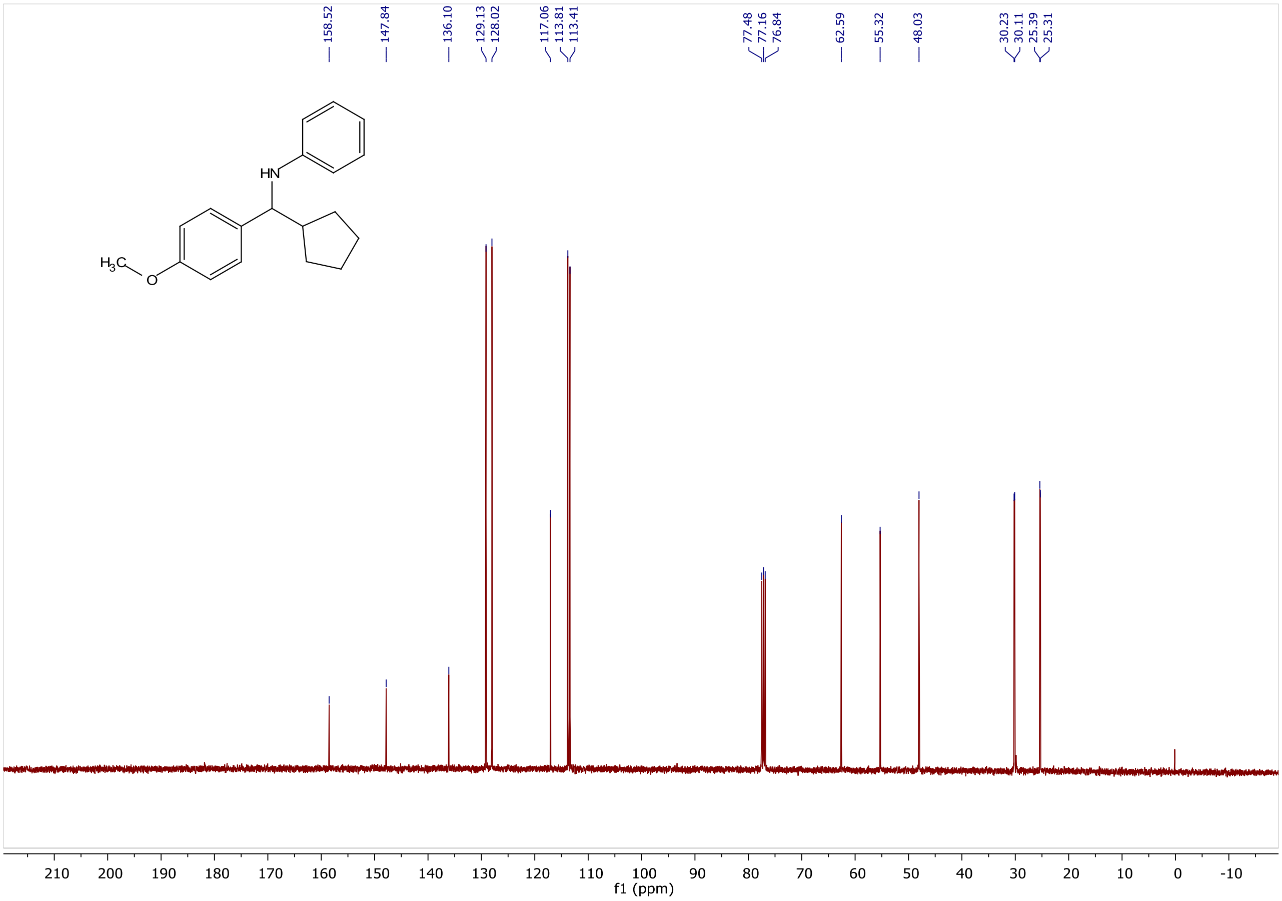

^{1}H NMR spectrum of compound **6b** ($CDCl_3$, 300 MHz).

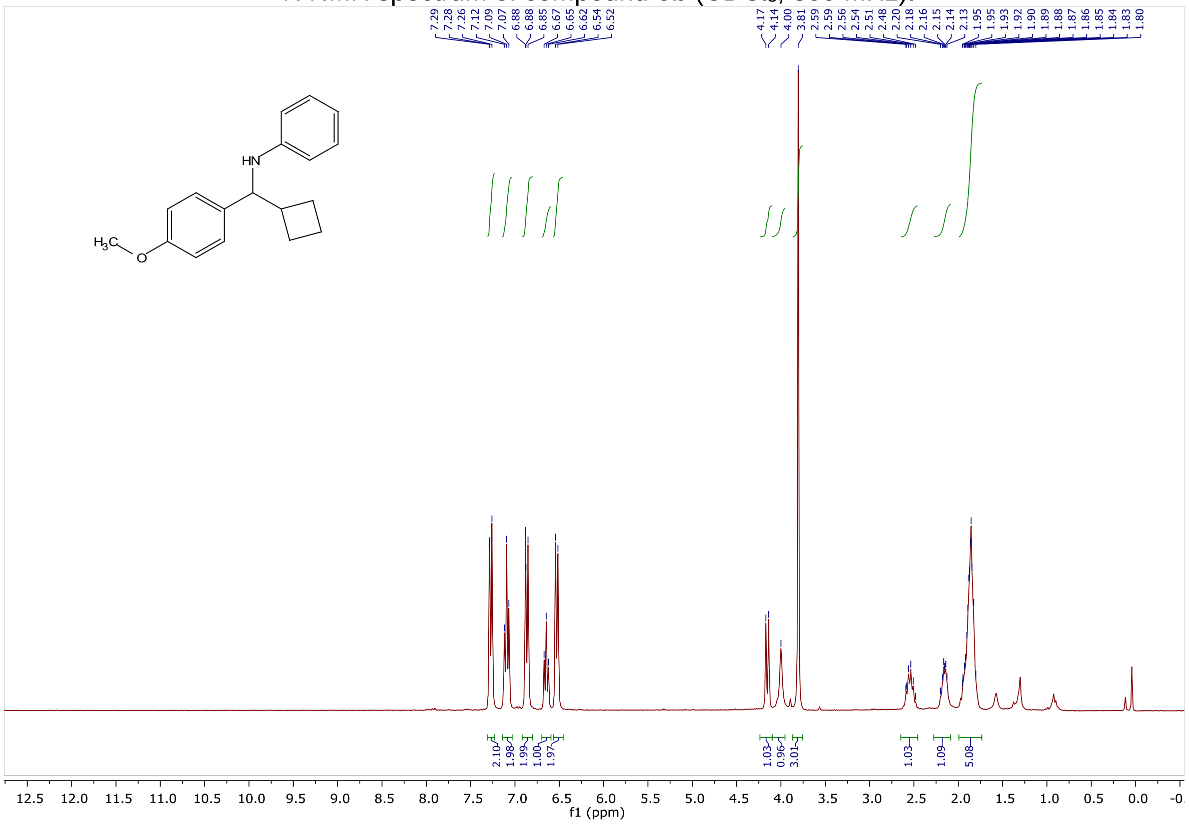

^{13}C NMR spectrum of compound **6b** ($CDCl_3$, 75 MHz).

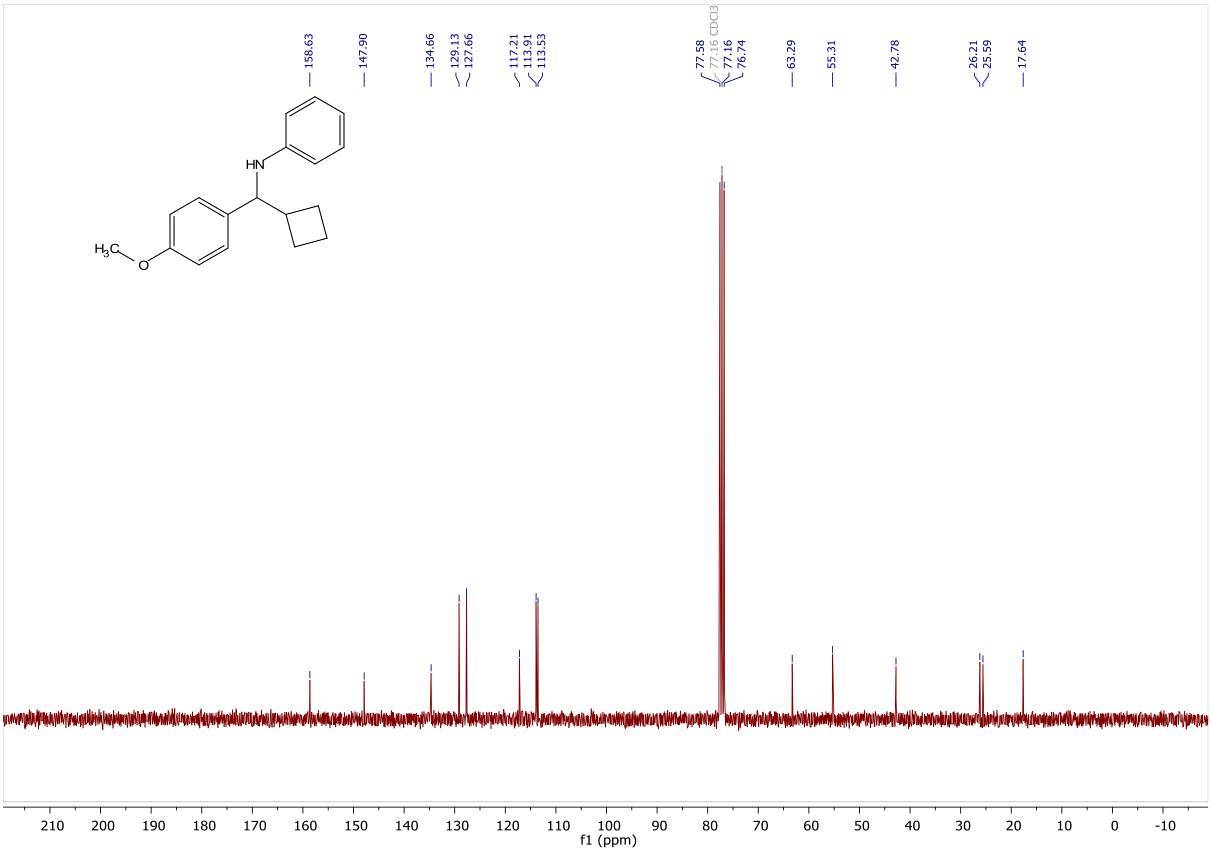

^{1}H NMR spectrum of compound **6c** ($CDCl_3$, 400 MHz).

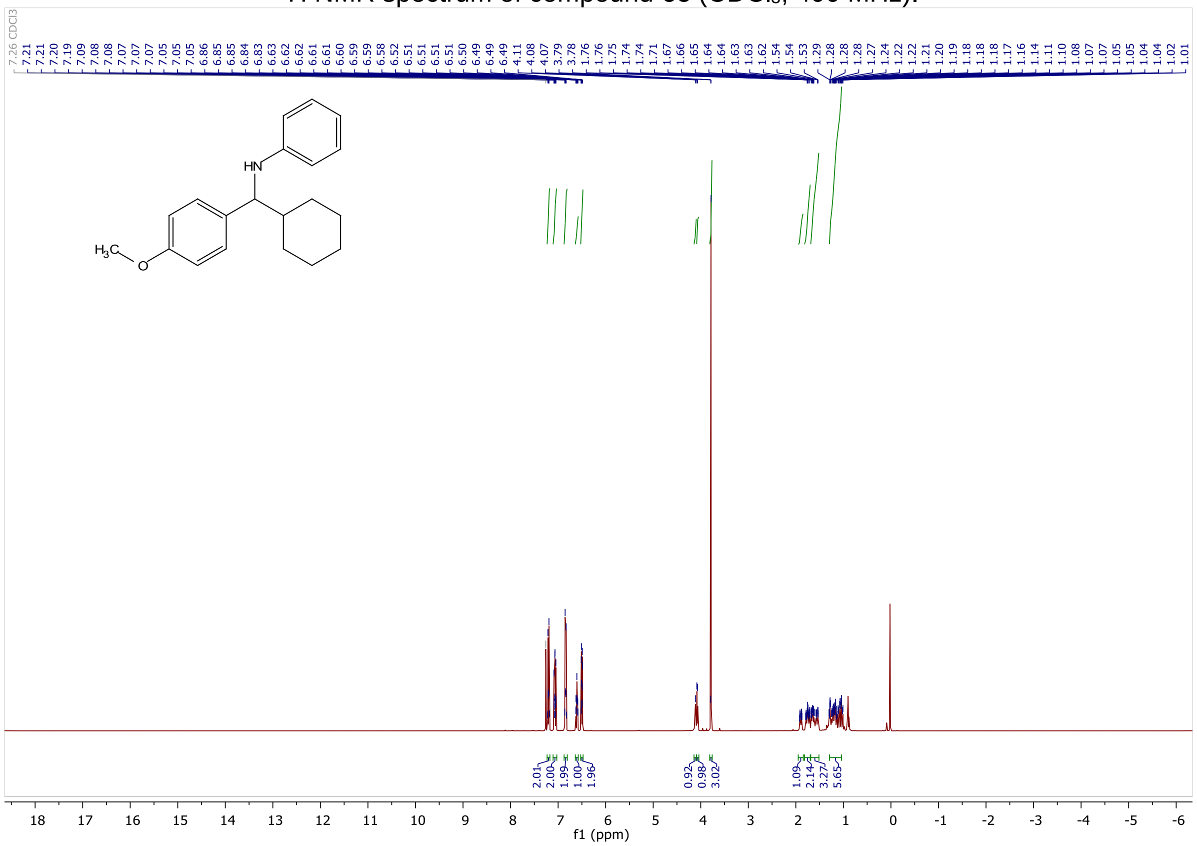

^{13}C NMR spectrum of compound **6c** ($CDCl_3$, 101 MHz).

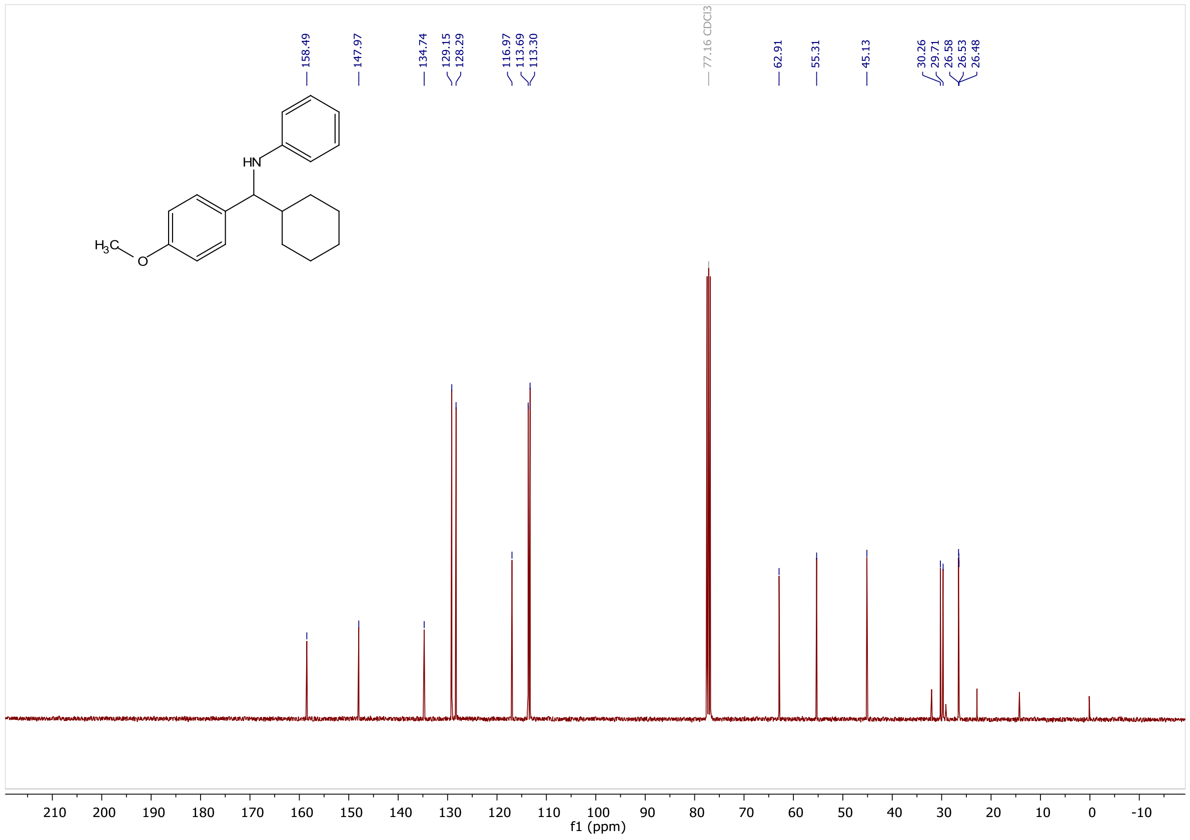

^{1}H NMR spectrum of compound **6d** ($CDCl_3$, 400 MHz).

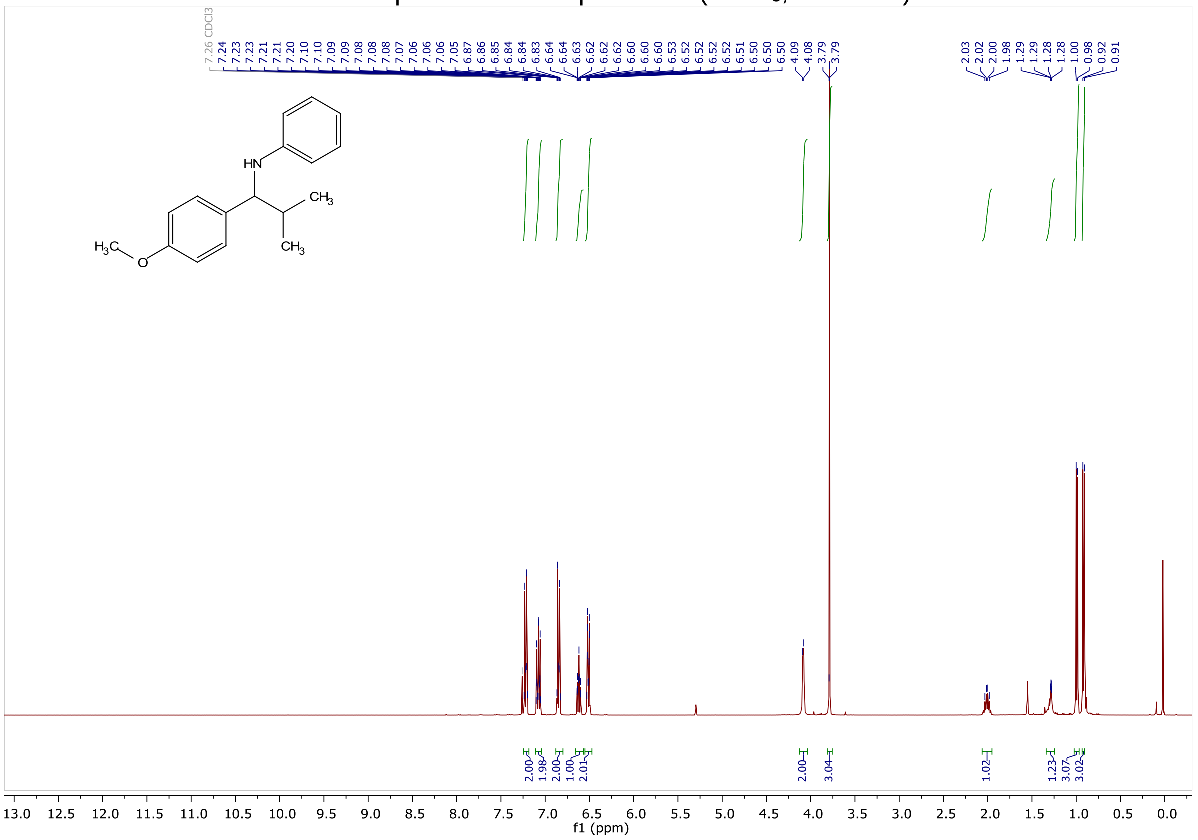

^{13}C NMR spectrum of compound **6d** ($CDCl_3$, 101 MHz).

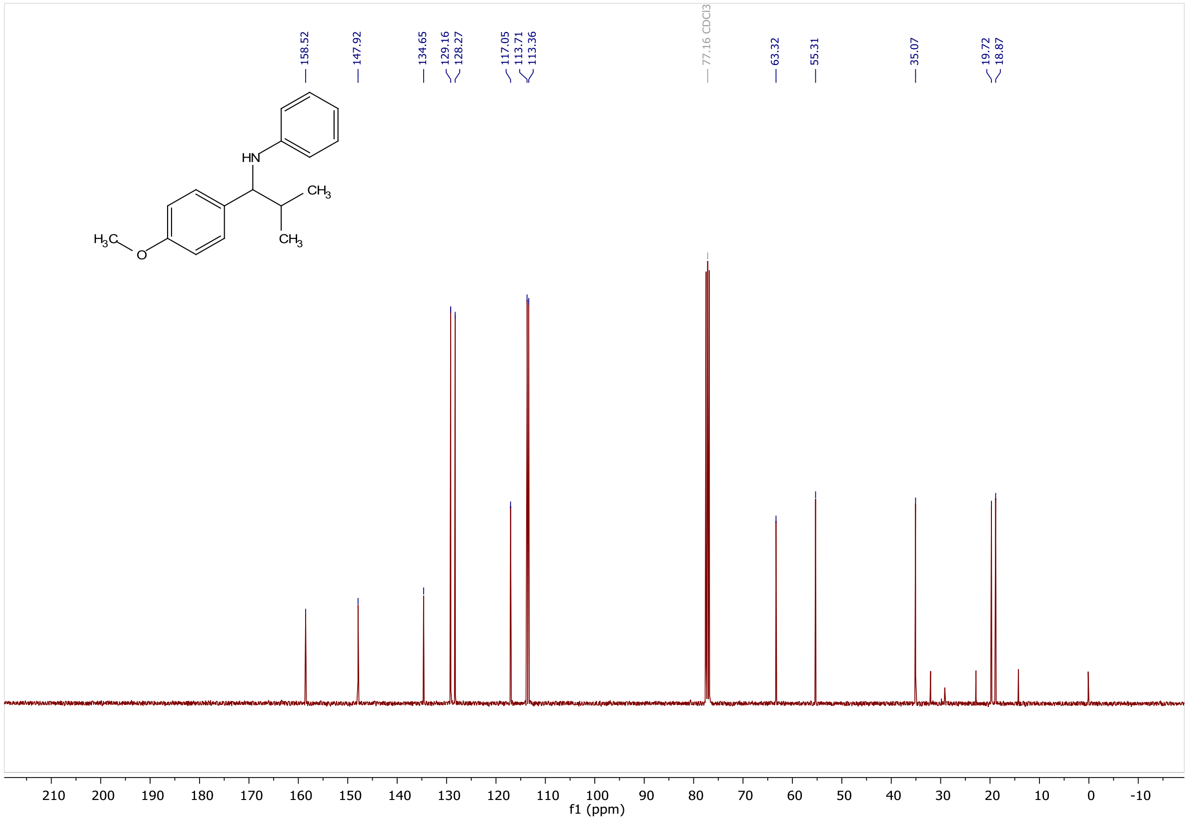

^{1}H NMR spectrum of compound **6e** ($CDCl_3$, 400 MHz).

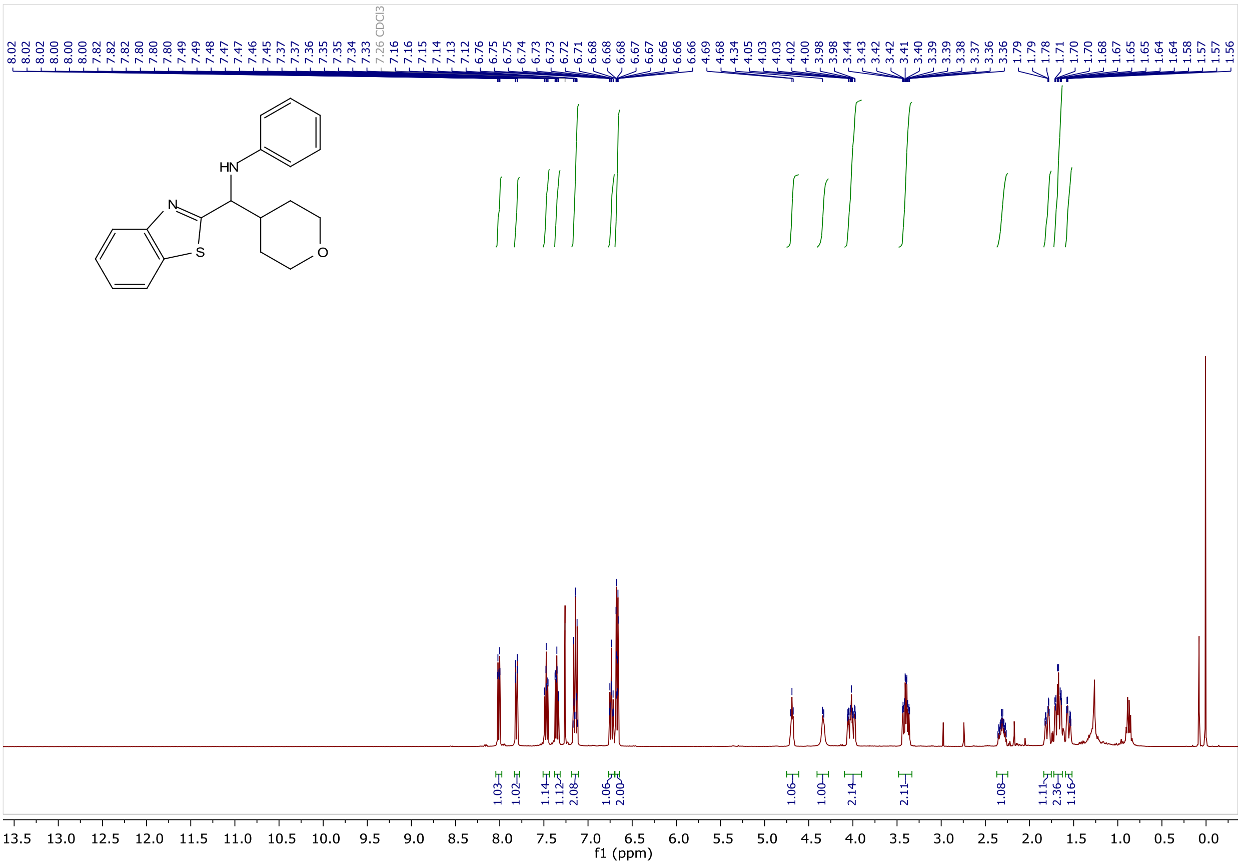

^{13}C NMR spectrum of compound **6e** ($CDCl_3$, 101 MHz).

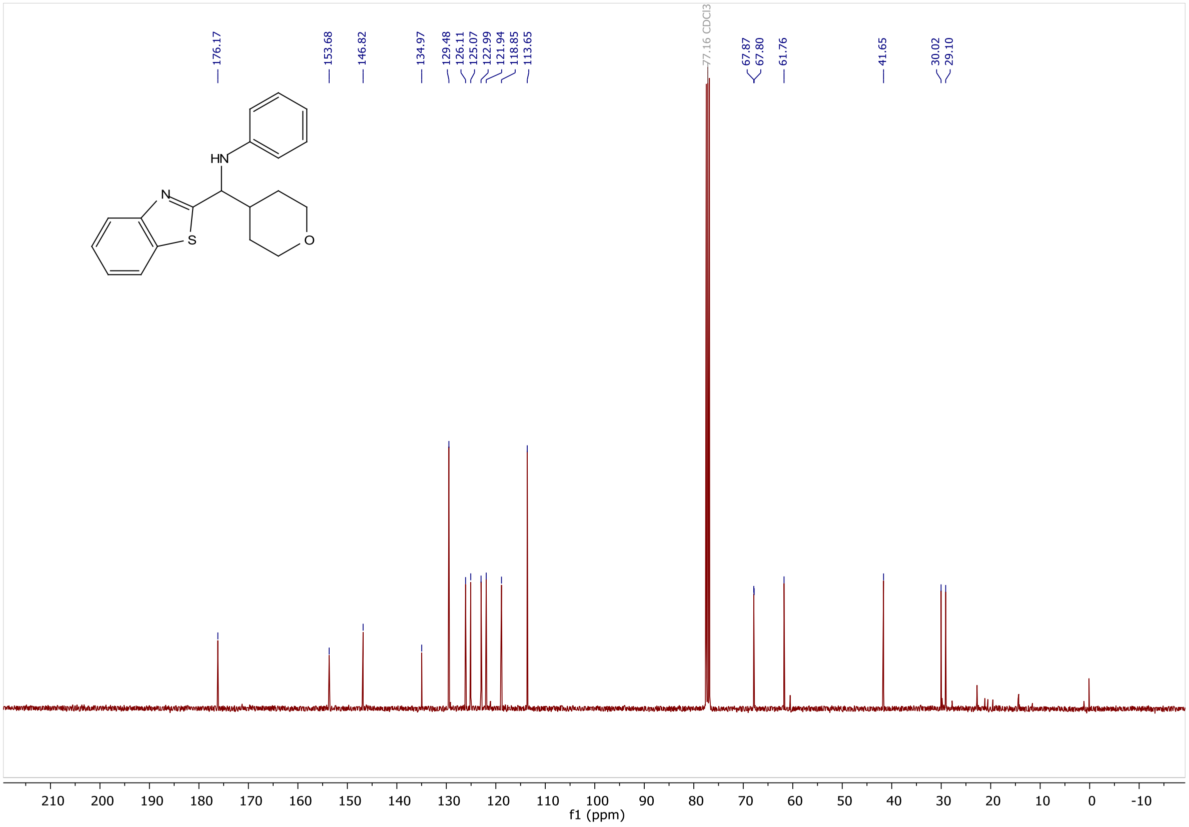

^{1}H NMR spectrum of compound **6f** ($CDCl_3$, 300 MHz).

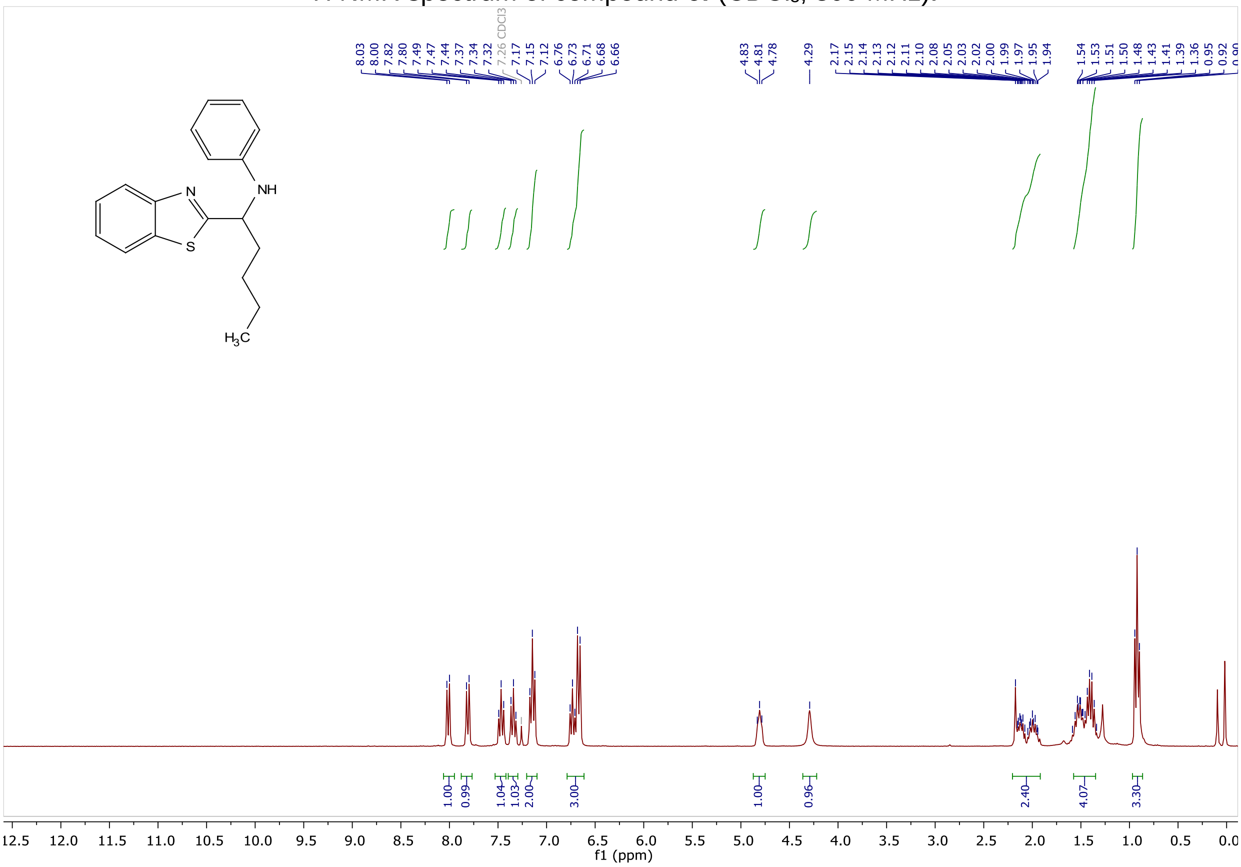

^{13}C NMR spectrum of compound **6f** ($CDCl_3$, 75 MHz).

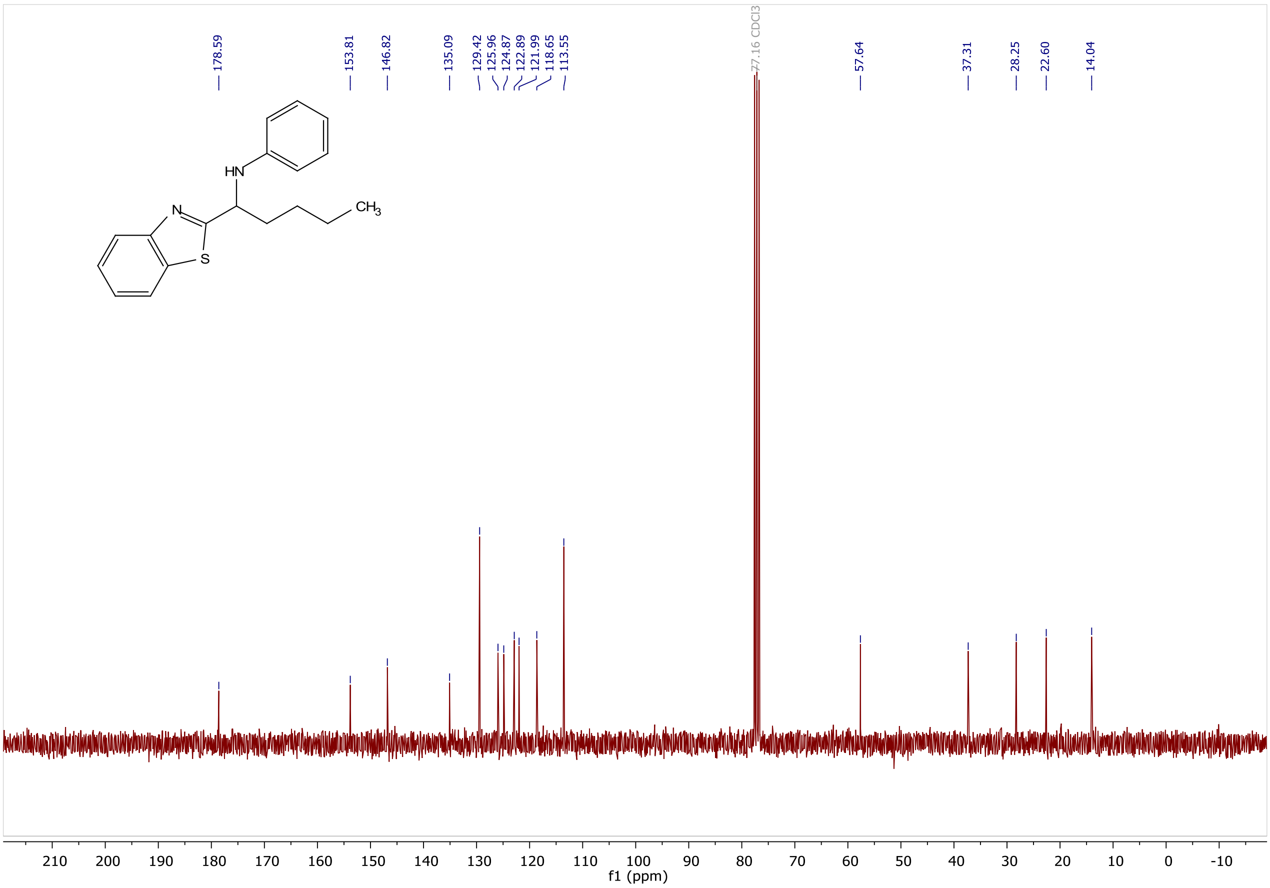

^{1}H NMR spectrum of compound **6g** ($CDCl_3$, 300 MHz).

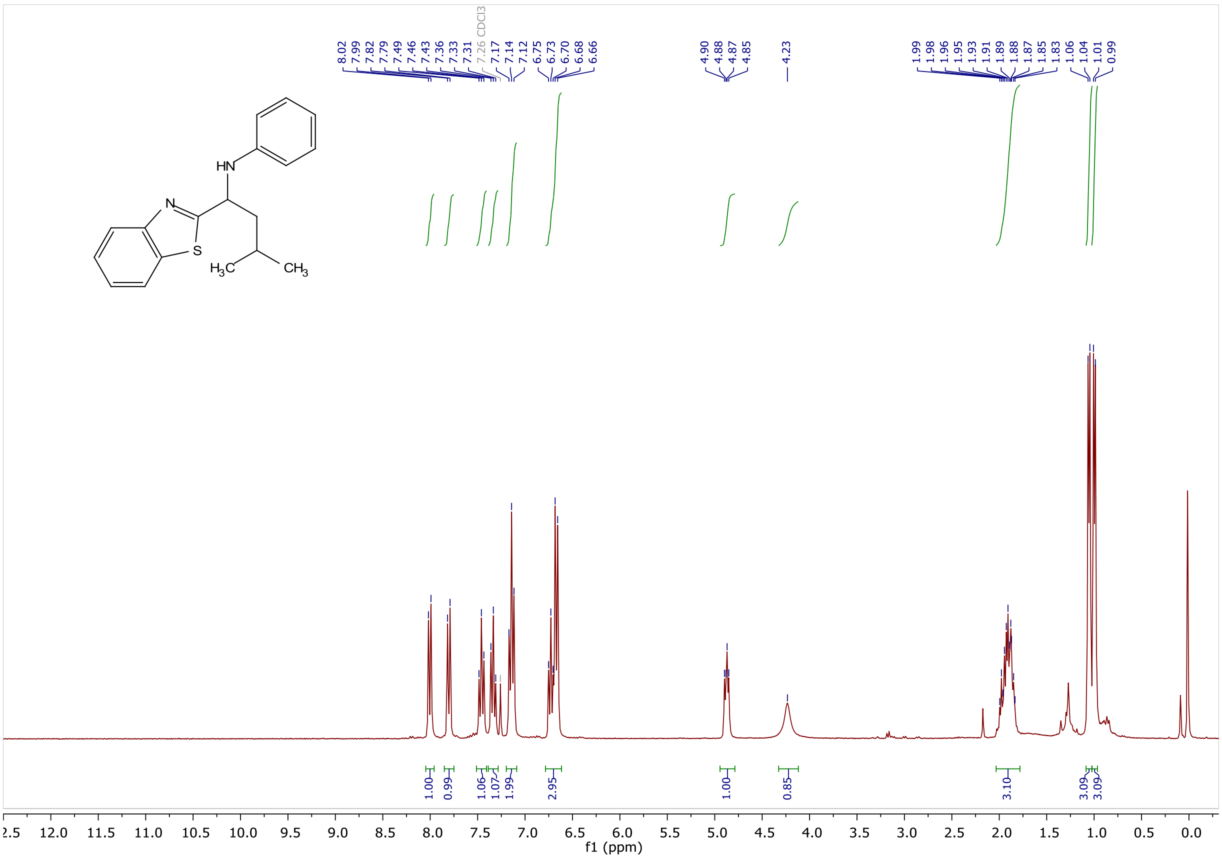

^{13}C NMR spectrum of compound **6g** ($CDCl_3$, 75 MHz).

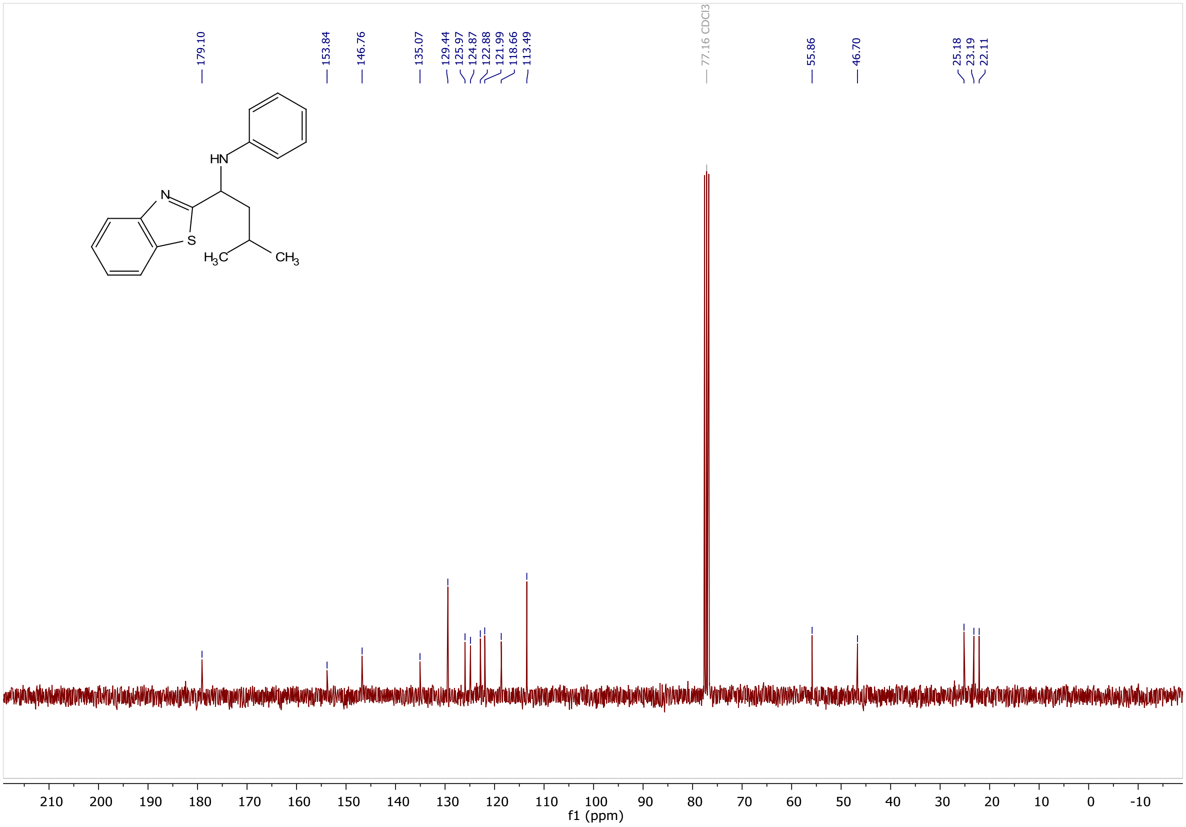

^{1}H NMR spectrum of compound **6h** ($CDCl_3$, 400 MHz).

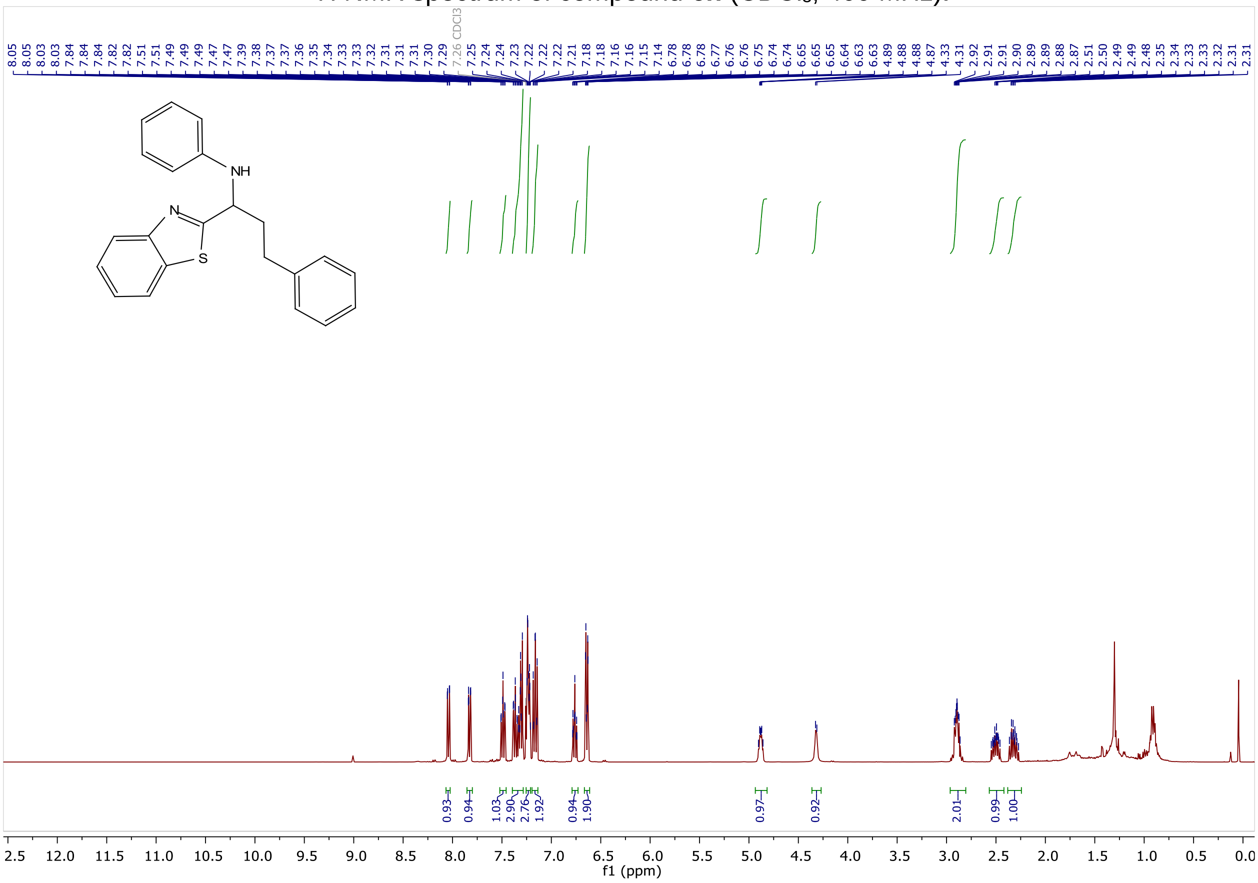

^{13}C NMR spectrum of compound **6h** ($CDCl_3$, 75 MHz).

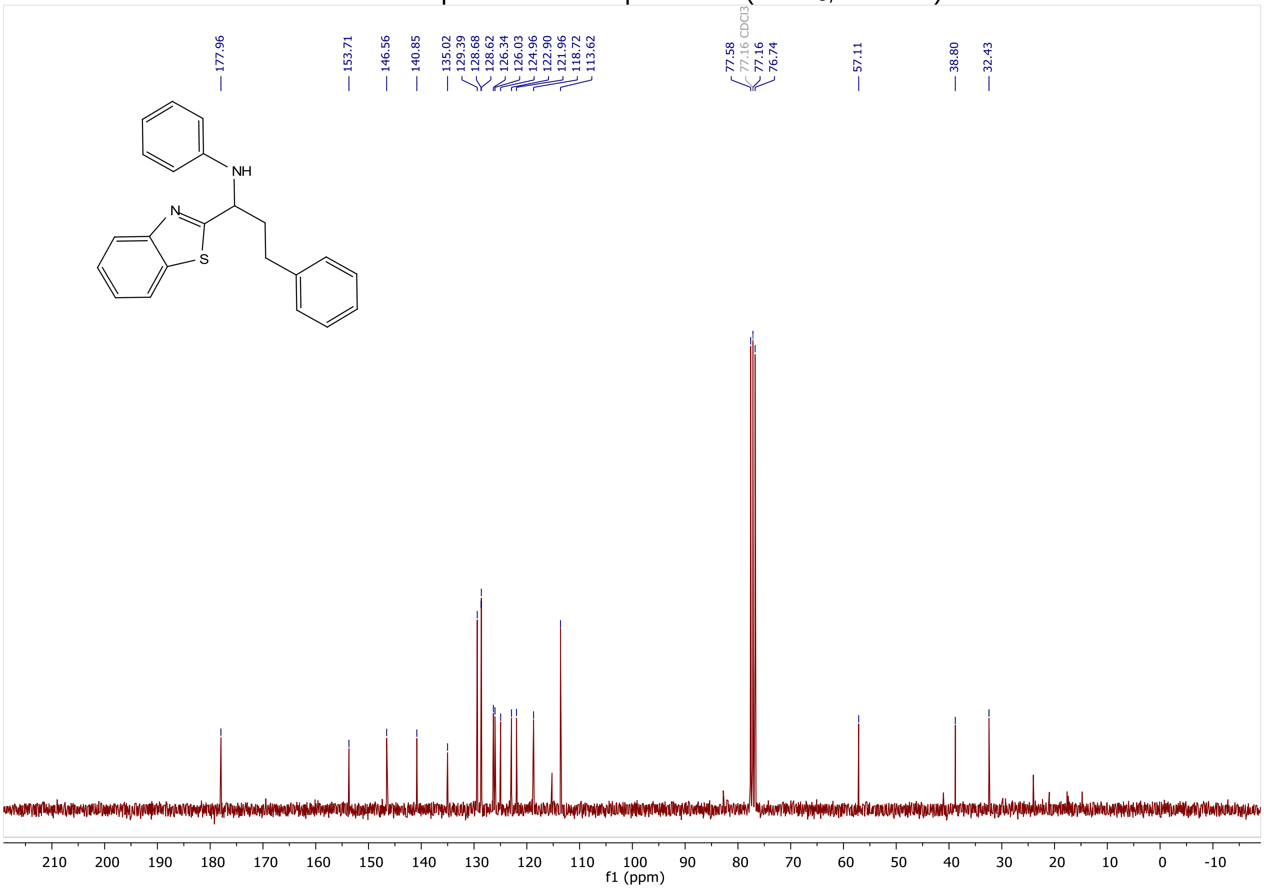

^{1}H NMR spectrum of compound **6i** ($CDCl_3$, 300 MHz).

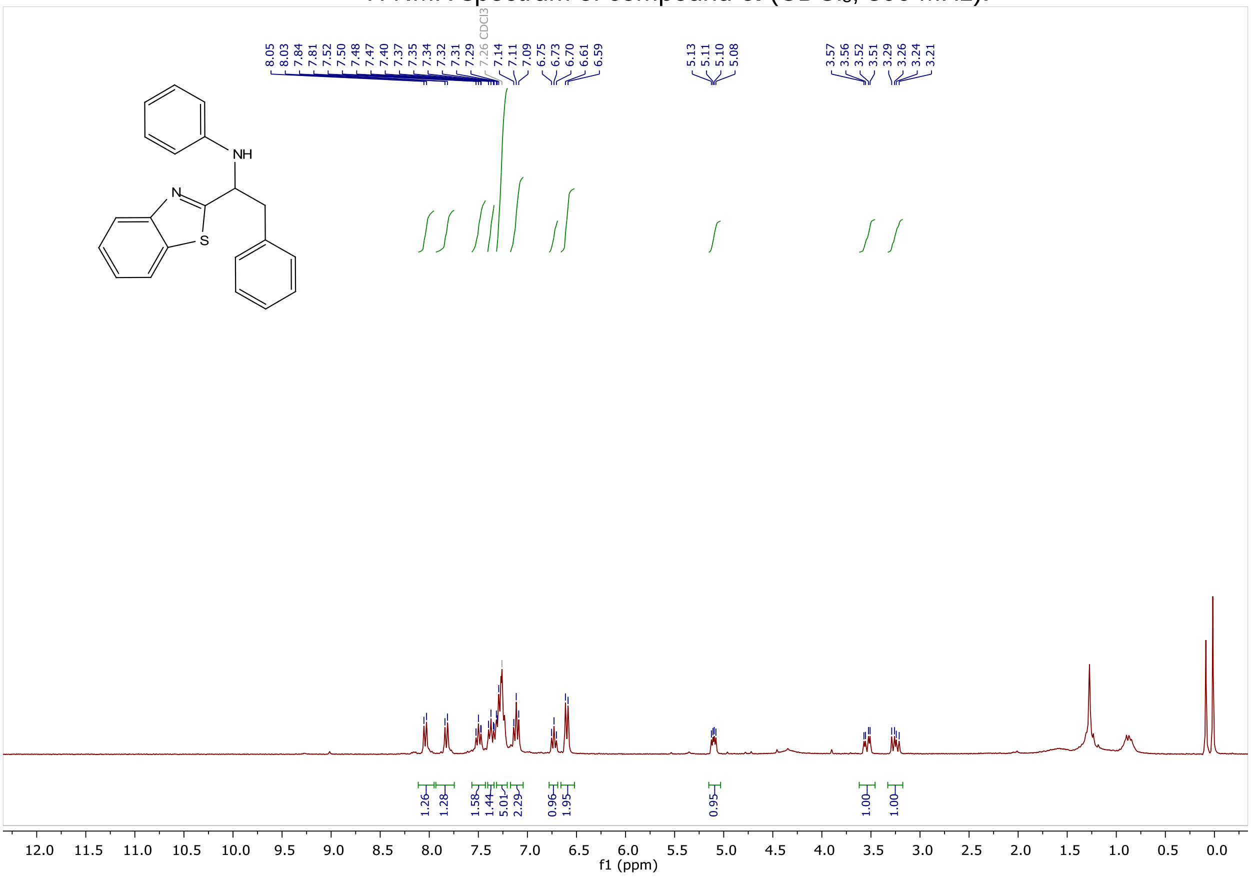

^{13}C NMR spectrum of compound **6i** ($CDCl_3$, 101 MHz).

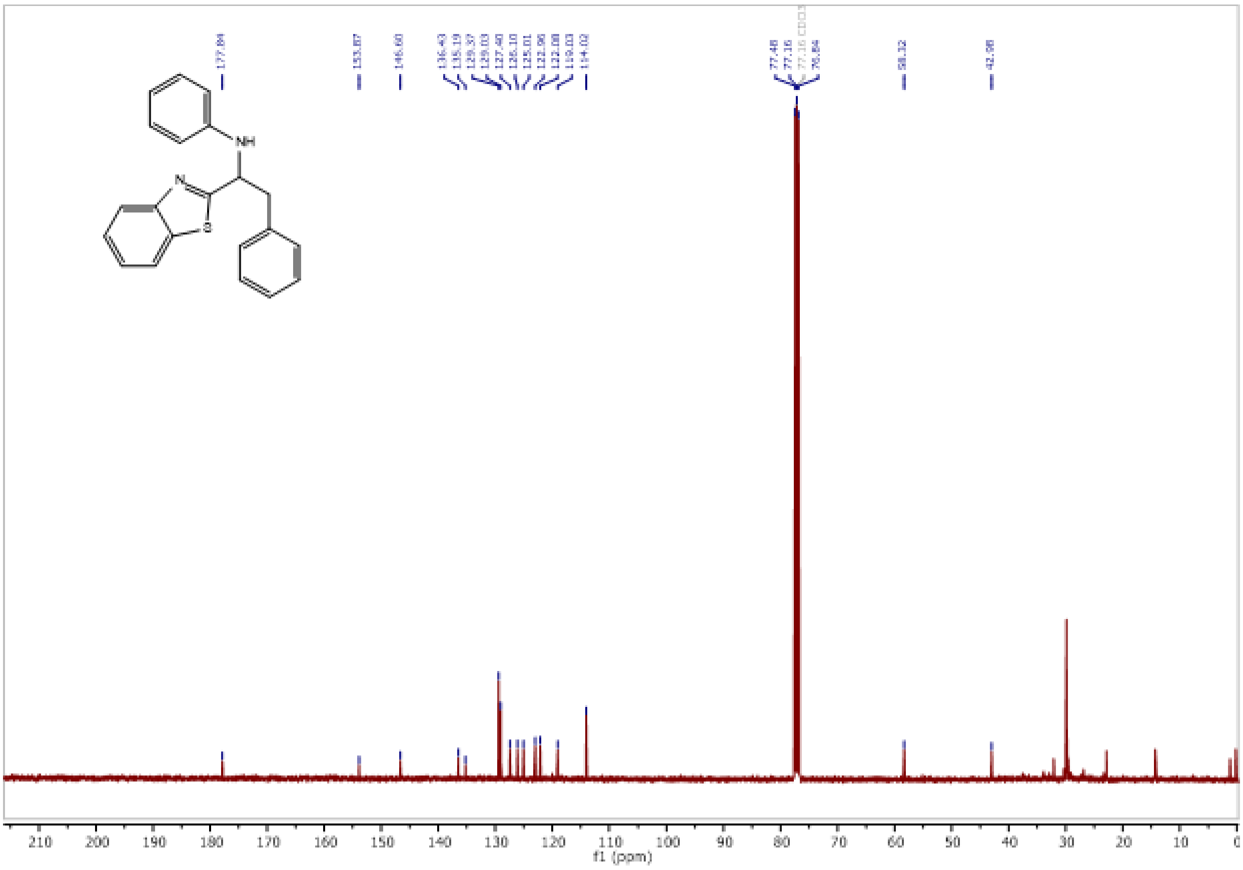

^{1}H NMR spectrum of compound **6j** ($CDCl_3$, 300 MHz).

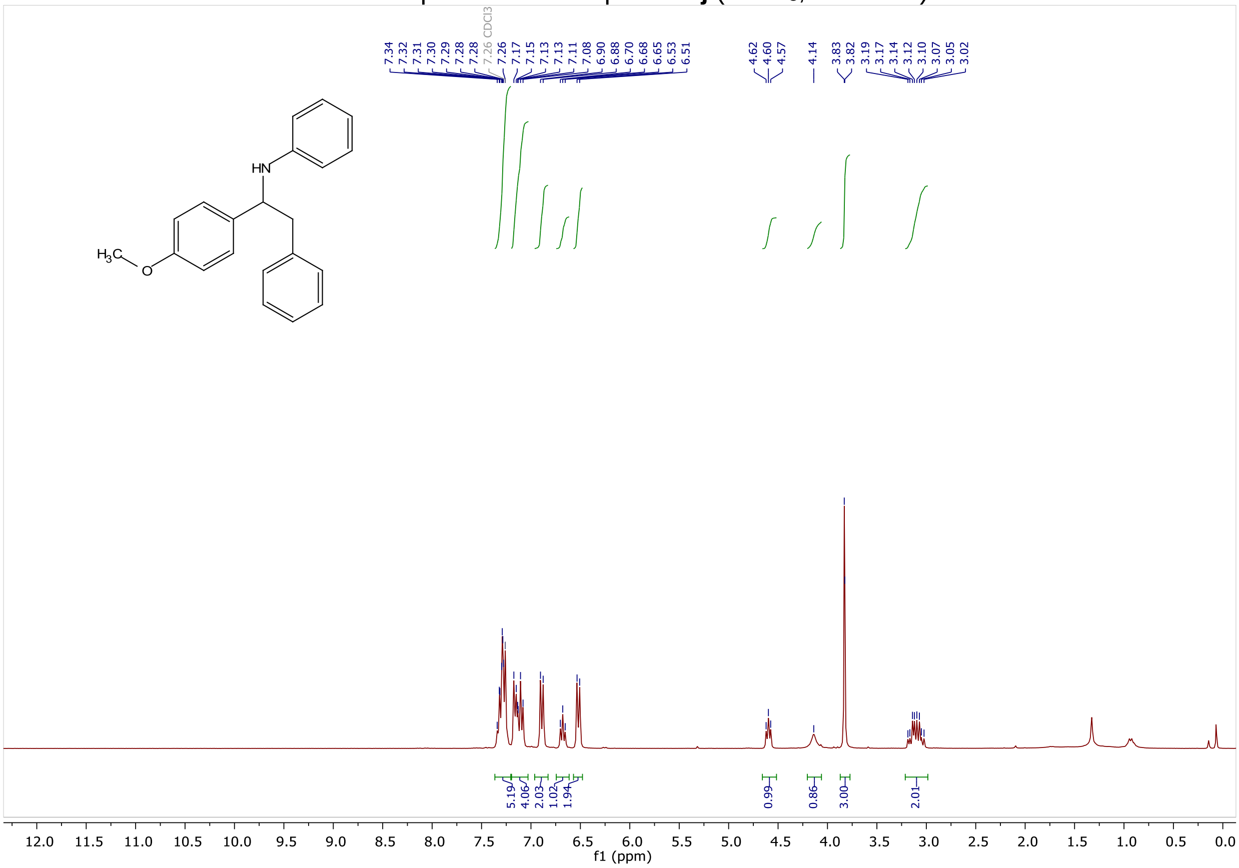

^{13}C NMR spectrum of compound **6j** ($CDCl_3$, 101 MHz).

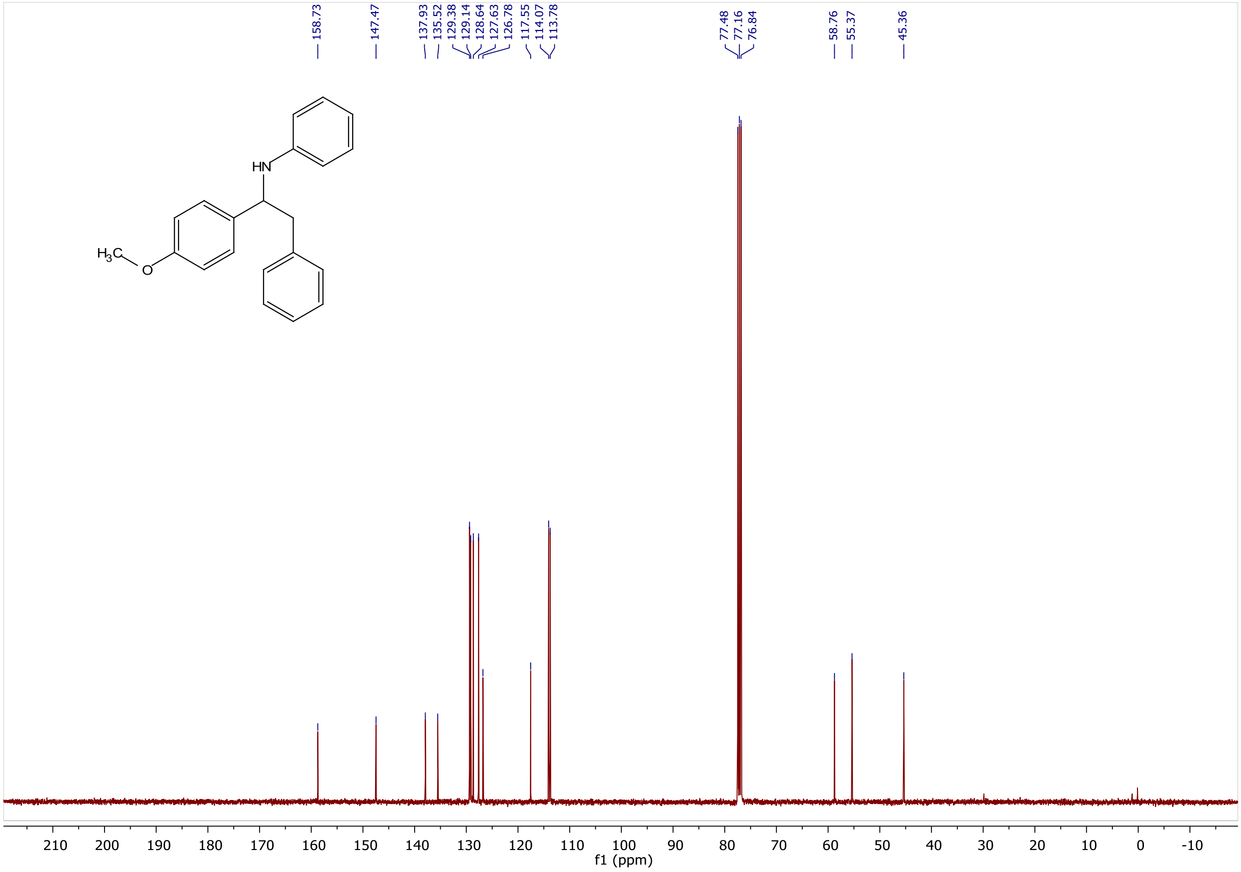

^{1}H NMR spectrum of compound **6k** ($CDCl_3$, 300 MHz).

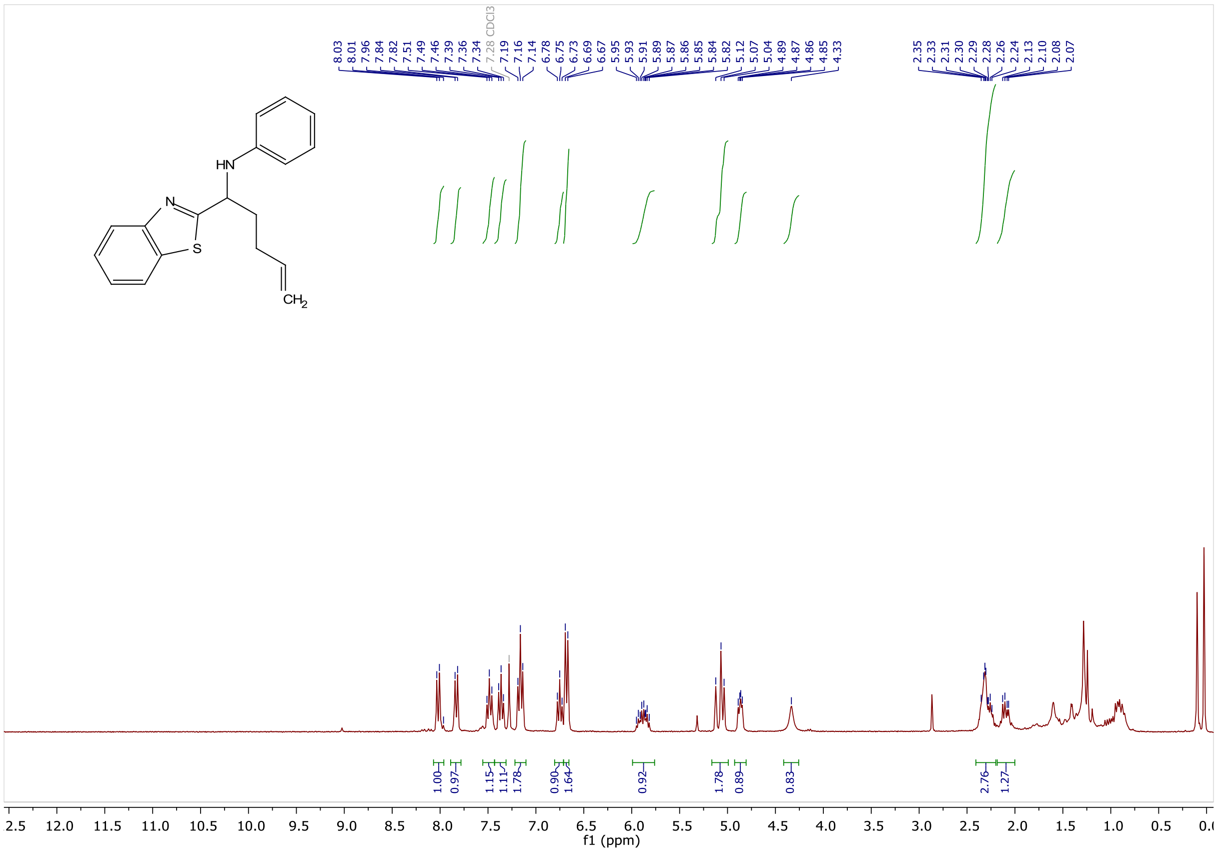

^{13}C NMR spectrum of compound **6k** ($CDCl_3$, 101 MHz).

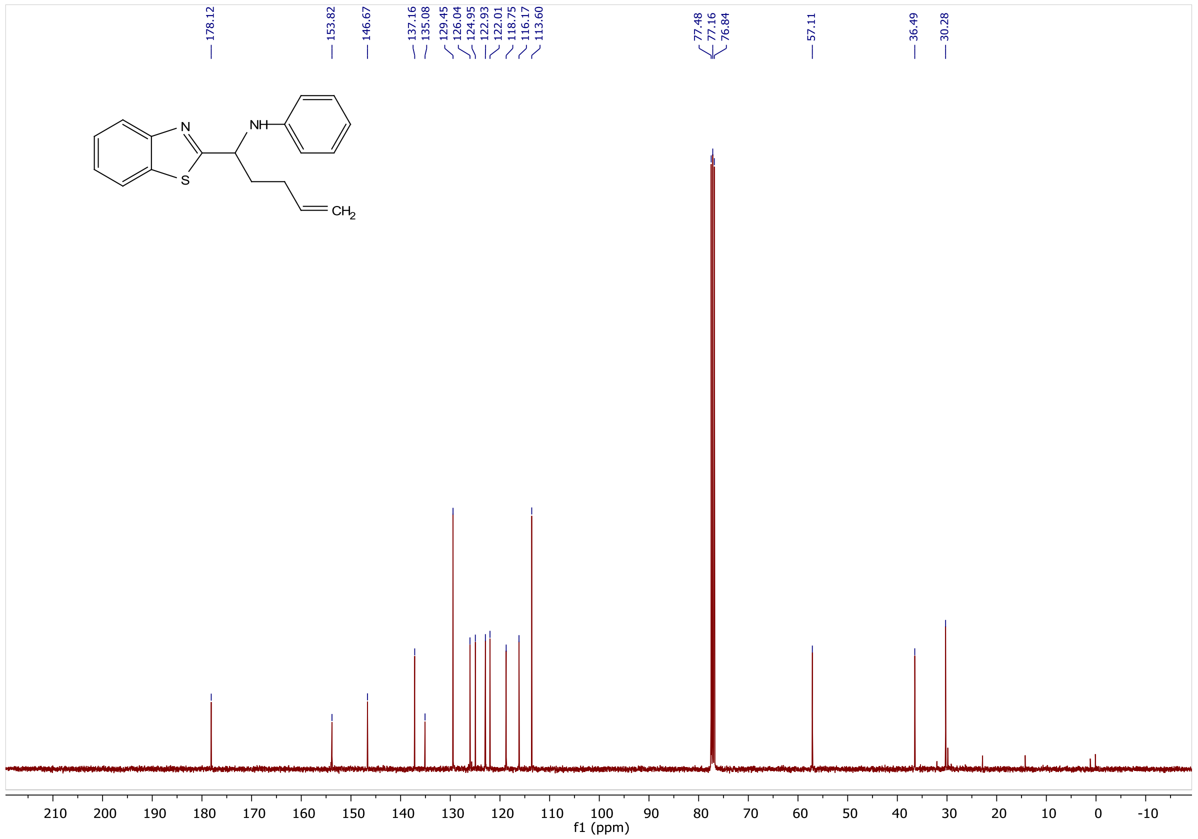

Supplement: Document S1. Figures S1–S3 and Scheme S1 [file mmc1.pdf]
